# New Thiazolyl-Pyrazoline Derivatives as Potential Dual EGFR/HER2 Inhibitors: Design, Synthesis, Anticancer Activity Evaluation and In Silico Study

**DOI:** 10.3390/molecules28217455

**Published:** 2023-11-06

**Authors:** Mariam M. Fakhry, Amr A. Mattar, Marwa Alsulaimany, Ebtesam M. Al-Olayan, Sara T. Al-Rashood, Hatem A. Abdel-Aziz

**Affiliations:** 1 Department of Pharmaceutical Chemistry, Faculty of Pharmacy, Egyptian Russian University, Badr 11829, Egypt; amr-a-mattar@eru.edu.eg; 2 Department of Pharmacognosy & Pharmaceutical Chemistry, College of Pharmacy, Taibah University, Medina 42353, Saudi Arabia; msulaimany@taibahu.edu.sa; 3 Department of Zoology, College of Science, King Saud University, Riyadh 11495, Saudi Arabia; eolayan@ksu.edu.sa; 4 Department of Pharmaceutical Chemistry, College of Pharmacy, King Saud University, Riyadh 11451, Saudi Arabia; salrashood@ksu.edu.sa; 5 Applied Organic Chemistry Department, National Research Center, Cairo 12622, Egypt

**Keywords:** thiazolyl-pyrazoline, anti-cancer, cell cycle, apoptosis, EGFR, HER2

## Abstract

**A** new series of thiazolyl-pyrazoline derivatives (**4a**–**d**, **5a**–**d 6a**, **b**, **7a**–**d**, **8a**, **b**, and **10a**, **b**) have been designed and synthesized through the combination of thiazole and pyrazoline moieties, starting from the key building blocks pyrazoline carbothioamides (**1a**–**b**). These eighteen derivatives have been designed as anticipated EGFR/HER2 dual inhibitors. The efficacy of the developed compounds in inhibiting cell proliferation was assessed using the breast cancer MCF-7 cell line. Among the new synthesized thiazolyl-pyrazolines, compounds **6a**, **6b**, **10a**, and **10b** displayed potent anticancer activity toward MCF-7 with IC_50_ = 4.08, 5.64, 3.37, and 3.54 µM, respectively, when compared with lapatinib (IC_50_ = 5.88 µM). In addition, enzymatic assays were also run for the most cytotoxic compounds (**6a** and **6b**) toward EGFR and HER2 to demonstrate their dual inhibitory activity. They revealed promising inhibition potency against EGFR with IC_50_ = 0.024, and 0.005 µM, respectively, whereas their IC_50_ = 0.047 and 0.022 µM toward HER2, respectively, compared with lapatinib (IC_50_ = 0.007 and 0.018 µM). Both compounds **6a** and **10a** induced apoptosis by arresting the cell cycle of the MCF-7 cell line at the G1 and G1/S phases, respectively. Molecular modeling studies for the promising candidates **6a** and **10a** showed that they formed the essential binding with the crucial amino acids for EGFR and HER2 inhibition, supporting the in vitro assay results. Furthermore, ADMET study predictions were carried out for the compounds in the study.

## 1. Introduction

Even with the massive global efforts made by scientists, cancer continues to be the most serious life-threatening disease. Approximately 10 million individuals succumb to cancer annually [1]. Cancer is a pathophysiological condition characterized by aberrations in the intrinsic mechanism of cell division [2]. There are numerous cancer treatment medications on the market and in the preclinical/clinical stages. Nonetheless, several issues such as drug resistance, selectivity, distribution, and/or penetration frequently restrict their efficacy [3]. As a result, tremendous progress has been made in the conception of the cellular, molecular environment, and systemic mechanisms governing the onset, progression, and metastatic spread of cancer [4,5,6,7].

Protein kinases (PKs) constitute a substantial and most significant protein family, implicated in a wide range of disorders. Any alteration to the PK-mediated pathways has the potential to cause diabetes, cancer, or inflammation. Pks are among the most desirable targets for drugs since they control a variety of biological processes, including the cell cycle, metabolism, DNA damage/repair, proliferation, apoptosis, and survival [8]. The EGFR (Epidermal Growth Factor Receptor) is known as the transmembrane tyrosine kinase receptor, which is subdivided into four distinct transmembrane receptors, namely EGFR (HER1), HER2, HER3, and HER4. They are essential for the regulation of cellular growth, proliferation, and apoptosis by transmitting extracellular stimuli to intracellular signal transduction pathways [9]. The upregulation of EGFR has been implicated in various malignancies, including breast, ovarian, and colon cancer [10,11,12]. The overexpression of EGFR has been observed in a majority of individuals diagnosed with triple-negative breast cancer (TNBC), exceeding 50% of cases. Therefore, the use of inhibitors that specifically target the EGFR may enhance the prognosis and overall results for patients diagnosed with TNBC [13]. Moreover, HER2 was demonstrated in 30% of early breast cancer cases [14,15].

Dual-target inhibitors of EGFR and HER2 have been observed to have superior therapeutic efficacy in comparison to single-target inhibitors. By simultaneously inhibiting both receptors, these inhibitors can effectively suppress the downstream signaling pathways that facilitate the proliferation and viability of tumors and can effectively mitigate resistance development [14,16]. Lapatinib, an EGFR/HER2 dual inhibitor, was approved for clinical use by the FDA in 2007, signifying its significant importance in the field [17,18,19].

**Figure 1:**
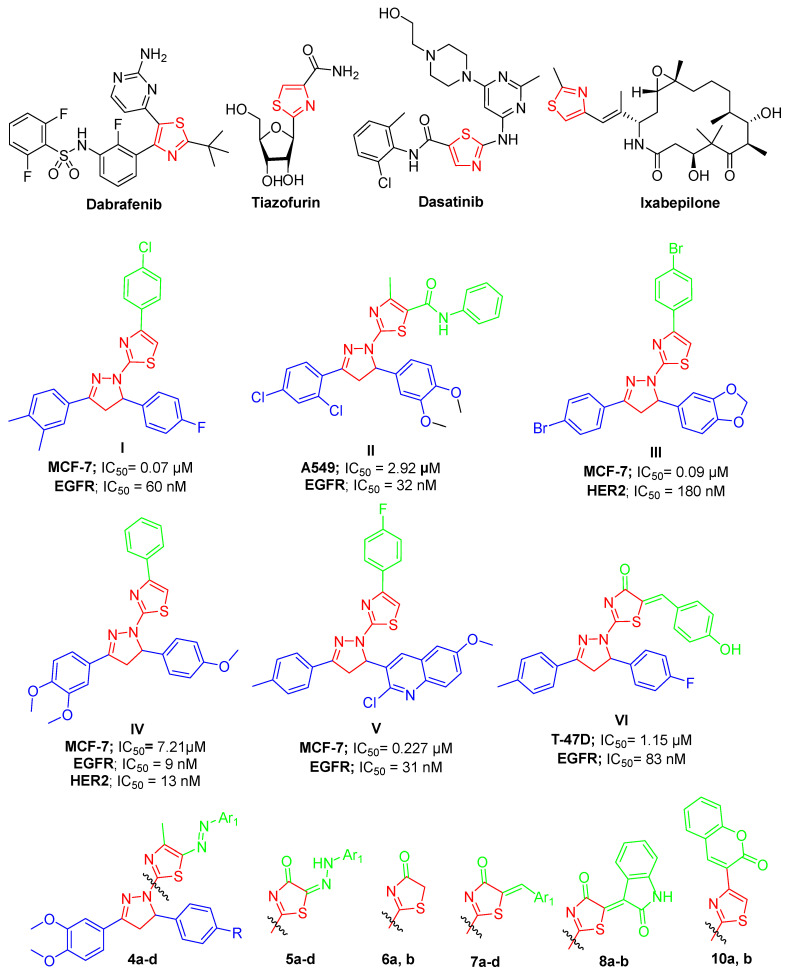
The structure of thiazole-based anticancer drugs, the reported potent anticancer thiazolyl-pyrazolines that act as EGFR and/or HER2 inhibitors (**I**–**VI**), and the targeted compounds **4a**–**d**, **5a**–**d**, **6a**, **b**, **7a**–**d**, **8a**, **b**, and **10a**, **b**.

Thiazole-based compounds have been recognized as a significant system that emerged as a hopeful fundamental scaffold in the discovery of anticancer drugs [20,21]. However, Dabrafenib [22,23], Tiazofurin [24], Dasatinib [25], and Ixabepilone [26] are thiazole-based anticancer drugs approved or under investigation for the treatment of cancer (Figure 1). Dabrafenib (Tafinlar^®^) is an inhibitor for the b-Raf enzyme used for cancers associated with the mutated gene BRAF, such as BRAF-mutated melanoma [23]. Tiazofurin is an inhibitor of the IMP dehydrogenase enzyme. It is a thiazole-based molecule with potential for cancer treatment. In addition, Dasatinib (Sprycel^®^) is a tyrosine-kinase inhibitor that blocks Bcr-AbI and Scr kinases. It is used in the treatment of certain cases of leukemia. Moreover, Ixabepilone is a semi-synthetic natural product that stabilizes microtubules and is a highly potent anticancer agent for the treatment of breast cancer. Moreover, thiazole-based inhibitors of kinases like phosphatidylinositol-3-kinases, EGFR [27], VEGFR [28], GSK-3*β* [29], and BRAF [30], exhibited promising results in preclinical studies.

On the other hand, the pyrazoline moiety showed interesting biological activities like anticancer [31], anti-inflammatory [32,33], analgesic [34], antidepressant [35], and antimicrobial activities [34,36,37]. Thiazole–pyrazoline molecular hybridization into a unified framework has shown a feasible and encouraging strategy to produce useful molecules for medicinal chemistry research. This approach developed a new class of compounds centered around the thiazolyl–pyrazoline system, which has subsequently been recognized as a significant framework in the exploration of anticancer, anti-inflammatory, and antimicrobial agents [38].

In addition, a considerable number of thiazolyl-pyrazoline derivatives exhibit significant cytotoxic activity, possessing potential inhibitions against EGFR and/or HER2. For example, thiazolyl–pyrazoline derivatives **I** and **II** revealed inhibition activity against EGFR with IC_50_ = 60 nM and 32 nM, respectively [39], whereas their analogue compound **III** showed inhibition activity with IC_50_ = 180 nM toward HER2 [40] (Figure 1).

Furthermore, phenylthiazolyl-pyrazoline **IV** [41] and compound **V** exhibited potent cytotoxicity for MCF-7 with EGFR inhibition (IC_50_ = 9 and 31 nM, respectively) [27]. For compound **VI**, it displayed antiproliferative activity for the breast cancer T-47D cell line with inhibition activity toward EGFR (IC_50_ = 83 nM) [42] (Figure 1).

According to the above data and in continuation of our scholarly interest in the chemistry of thiazolyl–pyrazolines and their biological activities [43,44,45], especially as possible anticancer agents [27,46,47], our objective was to enhance and modify a novel series of innovative thiazolyl-pyrazoline derivatives (**4a**–**d**, **5a**–**d**, **6a**, **b**, **7a**–**d**, **8a**, **b**, and **10a**, **b**) as potential dual-inhibitors against EGFR/HER2 with possible enhancement of their antiproliferative activities depending on introducing bioactive fragments like aryl-diazinyl, arylidene, isatin, or coumarin substitutions into the thiazole–pyrazoline combination as the core center (Figure 1). So, the basis and rationale of the design of target compounds as potential anticancer agents for breast cancer depend on the hybridization of the two bioactive scaffolds thiazole and pyrazoline which give greater anticancer activity by optimization of previous compounds by adding extra hydrophobic binding site and choosing the most active substituents (Cl and OCH_3_) in the previous work [48] which depends on inhibiting MCF-7 which represent a very important candidate as they are used ubiquitously in research for estrogen receptor (ER)-positive breast cancer cell experiments and many sub-clones, which have been established, represent different classes of ER-positive tumors with varying nuclear receptor expression levels. MCF-7 is a commonly used breast cancer cell line that has been propagated for many years by multiple groups and proves to be a suitable model cell line for breast cancer investigations worldwide, including those regarding anticancer [49] and evaluated against MCF-10a cells, which are the most common cell line used as a model for normal human breast cells. They are very structurally similar to normal human mammary epithelial cells and are usually supplemented with various factors such as hydrocortisone (HC), epidermal growth factor (EGF), and phenol red (PHR, a pH indicator), which can affect redox state as well as ER activity [49,50]. EGFR/HER2, the targets for our compounds, are highly expressed on both cell lines.

It is crucial to note that the pharmacophoric properties necessary for the inhibition of the EGFR family involve the presence of an adenine pocket located in the hinge region that necessitates a central flat hetero-aromatic ring scaffold in order to establish a crucial hydrogen bond. Additionally, a terminal hydrophobic head is required to occupy a hydrophobic sub-pocket, while a hydrophobic tail is necessary to occupy a second hydrophobic region. The hydrophobic pockets are important for increasing inhibitor selectivity [51,52]. Interestingly, the thiazolyl-pyrazoline scaffold achieved the required features for EGFR/HER2 inhibition.

The aim of this study is to optimize and increase the selectivity of the previous synthesized thiazolyl–pyrazoline series [48] by increasing the bulkiness on C4 or C5 of thiazole to find out the most effective scaffold. The synthesized thiazolyl–pyrazolines were investigated for their anticancer activity toward MCF-7. Their potential inhibitory effect of the promising compounds **6a**, **6b**, **10a**, and **10b** towards EGFR and HER2 kinases was assessed. After that, the most effective kinase and cell growth inhibitors, **6a** and **10a**, were then chosen in order to evaluate their potential biological mechanism of action using apoptosis in addition to the cell cycle analysis. Finally, the hybrid scaffolds for **6a** and **10a** were studied in silico to investigate their binding interactions at the ATP-binding sites in both kinases.

## 2. Results and Discussion

### 2.1. Chemistry

**Scheme 1:**
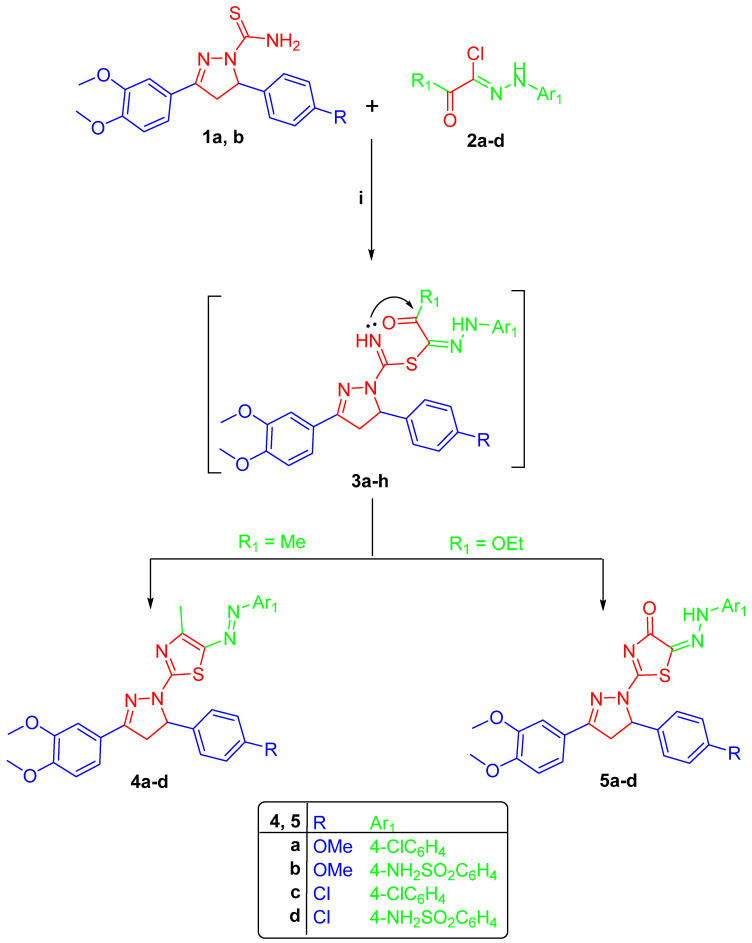
(**i**) Asolute EtOH, reflux, 6 h.

In first, the intermediate building blocks pyrazoline carbothioamide intermediates **1a**, **b** were accomplished by the reaction of precursor chalcone derivatives with thiosemicarbazide [48,53] (Scheme 1). The latter carbothioamides **1a**, **b** were refluxed with hydrazonoyl chloride derivatives (**2a**–**d**) [42] in absolute ethanol to form the S-alkylated non-isolable intermediate **3a**–**h**, which could undergo cyclization via nucleophilic addition followed by the loss of water, in case of **2a**, **b**; R_1_ = Me, or the loss of ethanol, in case of **2c**, **d**; R_1_ = OEt, to furnish the 5-(aryldiazenyl)4-methylthiazoles **4a**–**d** and 2-(arylhydrazineylidene)thiazol-4(5*H*)-ones **5a**–**d**, respectively (Scheme 1).

^1^H NMR spectra of 5-(aryldiazenyl)4-methylthiazoles **4a**–**d** supported the proposed structures, revealing the presence of singlets in the range *δ* 2.48–2.52 ppm corresponding to thiazole CH_3_ protons. Moreover, ^1^H NMR of **4a**–**d** showed the common pattern of pyrazoline protons that appeared as three doublets of doublet around *δ* 3.4, 4.1, and 5.8 ppm and two singlet signals for the protons of two -OCH_3_ groups at *δ* 3.7 and 3.8 ppm. The ^13^CNMR of **4a**–**d** revealed the signals of -CH_3_ carbon at *δ* 16.1–16.5 ppm, signals of -OCH_3_ groups carbon at *δ* 55.1–55.7 ppm, and the signals of C4 and C5 of pyrazoline at *δ* 43.7 and 62.5 ppm, respectively. On the other hand, the IR spectra of the 2-(arylhydrazineylidene)thiazol-4(5*H*)-one **5a**–**d** revealed the absorption band of the C=O group at 1668-1699 cm^−1^. The ^1^H NMR spectra of **5a**–**d** exhibited the D_2_O exchangeable singlet signal in the range *δ* 10.58–11.05 ppm for the hydrazone NH proton. The ^13^C NMR spectra displayed a signal of thiazolone C=O at *δ* 175 ppm. Moreover, the IR spectra of compounds **4b**, **4d**, **5b**, and **5d** showed the characteristic bands corresponding to the NH_2_ group at 3314, 3342, and 3232, 3241 cm^−1^.

**Scheme 2:**
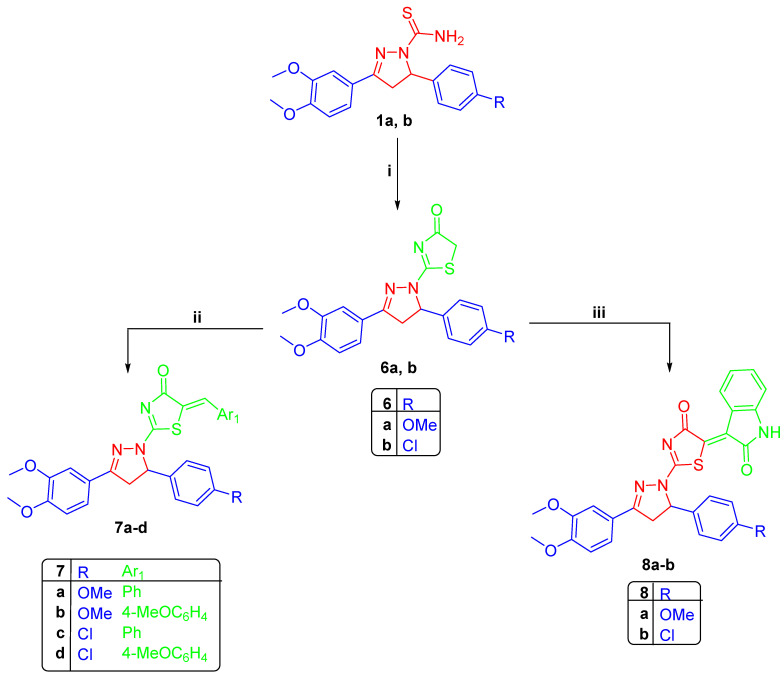
(**i**) Bromoacetic acid, AcOH, anhydrous AcONa, reflux 8 h; (**ii**) Ar_1_-CH=O, EtOH, piperdine, reflux 24 h; (**iii**) Isatin, AcOH, anhydrous AcONa, reflux 16 h.

Next, the carbothioamide **1a**, **b** refluxed with bromoacetic acid in acetic acid to afford the thiazolones **6a**, **b**, respectively, which reacted in refluxing absolute ethanol with different substituted aldehydes using a catalytic amount of piperidine to afford the arylidines **7a**–**d** (Scheme 2). Additionally, thiazolones **6a**, **b** were reacted with isatin in refluxed glacial acetic acid in the presence of anhydrous AcONa to give compounds **8a**, **b**, respectively.

The IR spectra of thiazolones **6a**, **b** revealed a band at 1740–1693 cm^−1^ due to C=O. ^1^H NMR spectra of **6a**, **b** exhibited a singlet signal of thiazolone CH_2_ protons around *δ* 3.9 ppm. ^13^C NMR displayed a signal at *δ* 39.2 assigned to CH_2_ of the thiazolone ring and a signal at *δ* 187.2 assigned to the C=O carbon of thiazolone. Regarding arylidines **7a**–**d**, their ^1^H NMR showed the signal of the -CH=N- proton, which was detected around *δ* 7.40 ppm*,* whereas their ^13^C NMR showed the disappearance of the CH_2_ signal at *δ* 39.2.

Regarding compounds **8a**, **b**, their IR spectra displayed a characteristic absorption band at 3307–3312 cm^−1^ due to NH function in addition to absorption bands of carbonyl groups of thiazolone and isatin around 1680 cm^−1^. The ^1^HNMR spectra of **8a**, **b** showed the D_2_O exchangeable signal of the NH proton around *δ* 11.1 ppm*,* whereas their ^13^C NMR displayed two signals of carbonyl carbons for thiazolone and isatin around *δ* 172 and 179 ppm.

**Scheme 3:**
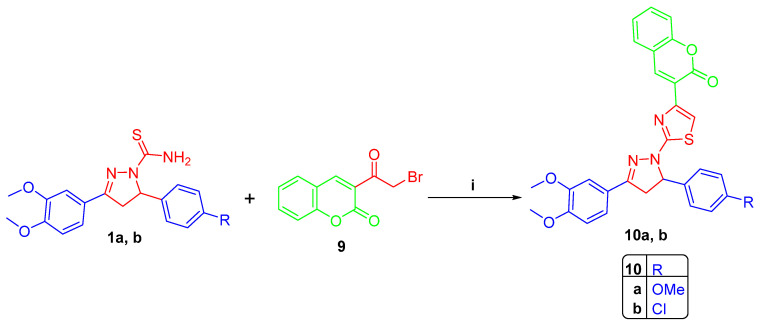
(**i**) Absolute EtOH, reflux 6 h.

Finally, the 2-bromoacetyl coumarin **9**, which was synthetized via bromination of acetyl coumarin using Br_2_ in glacial acetic acid [54], was reacted with carbothioamide **1a**, **b** in refluxed absolute EtOH to furnish compounds **10a**, **b**, respectively (Scheme 3). The IR spectra of **10a**–**b** showed the absorption band of coumarin C=O at 1730–1711 cm^−1^. ^1^HNMR spectra for compounds **10a**, **b** revealed a singlet signal around *δ* 7.8 ppm due to thiazole H5. ^13^C NMR for compounds **10a**–**b** showed a signal around *δ* 164 ppm for the C=O of coumarin.

### 2.2. Biological Evaluation

#### 2.2.1. Anti-CancerActivity against MCF-7 and MCF-10A

The eighteen newly synthesized thiazolyl–pyrazoline derivatives were evaluated for their cytotoxic activity toward MCF-7 using the MTT assay, and their IC_50_ values were evaluated in relation to Lapatinib. Among the screened compounds, four compounds (**6a**, **6b**, **10a**, and **10b**) revealed significant anticancer activity with IC_50_ = 4.08, 5.64, 3.37, and 3.54 µM against MCF-7 when compared to Lapatinib (IC_50_ = 5.88 µM) (Table 1).

On the other side, compounds **6a** and **10a** showed non-cytotoxic activity against MCF-10A compared with Lapatinib, where IC_50_ ≥ 50 µM for all of them; hence, compounds **6a** and **10a** were assumed to be of value to be investigated for the effective molecular target and apoptosis in MCF-7 cells (Table 2).

#### 2.2.2. SAR Studies

**Figure 2:**
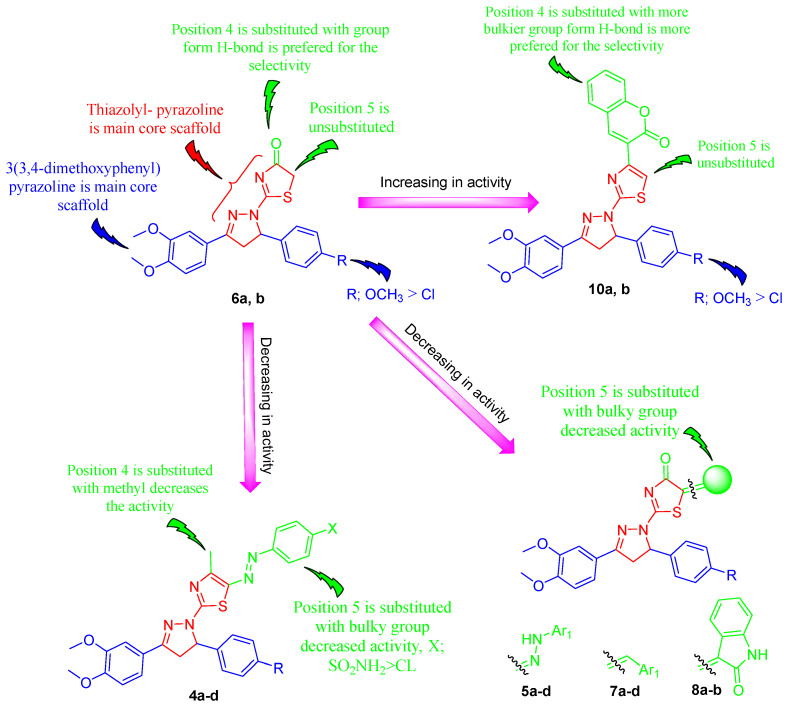
Structure features of the synthesized series relative to their cytotoxic IC_50_ values**.**

According to the results in Table 1, we can conclude some correlations between the structure of the study compounds and their anticancer activity. Generally, compounds 5-(4-methoxy phenyl) pyrazoline are more active than compounds 5-(4-chloro phenyl) pyrazoline. For the thiazole, the substitution on C5 decreases the potent activity; therefore, series **6a**, **b** and **10a**, **b** are more potent than the other designed series. The substitution on C4 is preferred to be an H-bond acceptor group, and the bulkier group is more preferred, so series **10a**, **b** is more potent than series **6a**–**b**. For C4 substitution, it is worth noting that for series **4a**–**d** and **5a**–**d**, the *p*-SO_2_NH_2_ phenyl derivative is more active than *p*-Cl phenyl derivatives. Series **5a**–**d**, **7a**–**d**, and **8a**–**b** showed relatively less activity compared to **6a**–**b** owing to the unavailable carbonyl involved in the intramolecular H-bond (Figure 2).

#### 2.2.3. EGFR and HER2 Kinase Inhibitory Assay

To emphasize the mechanistic studies, the most potent compounds (**6a**, **6b**, **10a**, and **10b**) towards MCF-7 cells were evaluated against their EGFR/HER2 inhibitory activities. The compounds under investigation showed excellent dual inhibitions of EGFR and HER2. Interestingly, compounds **6a** and **10a** showed IC_50_ = 0.024 and 0.005 µM, respectively, toward EGFR and IC_50_ values of 0.047 and 0.022 µM, respectively, against HER2, in a comparative way, like Lapatinib (Table 3). Therefore, both compounds **6a** and **10b** were worth testing for their apoptosis induction, owing to their significant cytotoxic activity and EGFR/HER2 inhibition activity.

#### 2.2.4. Cell Cycle Analysis

**Figure 3:**
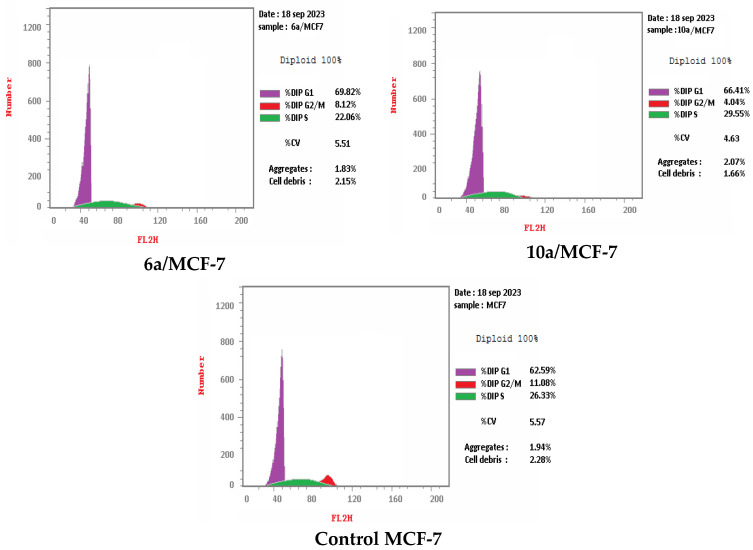
Cell cycle analysis of compounds **6a** and **10a** in MCF-7 at concentrations of 4.08 and 3.37 µM.

The specific phase of cell cycle arrest for compounds **6a** and **10a** at their respective IC_50_ values in the MCF-7 cell line was determined (Table 4 and Figure 3). In addition, **6a** exhibited high cell accumulation (69.82%, 1.16 folds) for the MCF-7 cell line at G0-G1 (control; 62.59) whereas **10a** arrests the cell cycle of the MCF-7 cell line at G1 and S phases with 66.41% (1.06 folds) and 29.55% (1.12 folds) compared with the control (62.59% and 26.33%, respectively).

#### 2.2.5. Apoptosis Analysis

**Figure 4:**
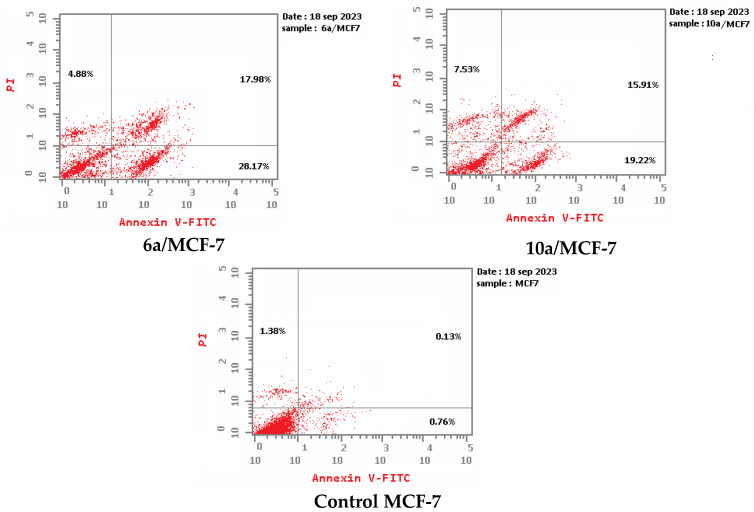
Apoptosis analysis of **6a** and **10a** in MCF-7 cells.

To investigate the exact cause of apoptotic activity of **6a** (IC_50_ = 4.08 μM) and **10a** (IC_50_ = 3.37 μM) in MCF-7 cells using flow cytometric analysis of Annexin V/PI staining. Compounds **6a** and **10a** showed significant total apoptotic death with 51.03% (28.17% and 17.98% for early and late apoptosis, respectively), whereas compound **10a** exhibited total apoptosis of 42.66% (19.22% and 15.91% for early and late apoptosis, respectively), when compared to total apoptosis of 2.27% in the control MCF-7 (Table 5 and Figure 4).

### 2.3. Computational Studies

#### Molecular Modelling

**Figure 5:**
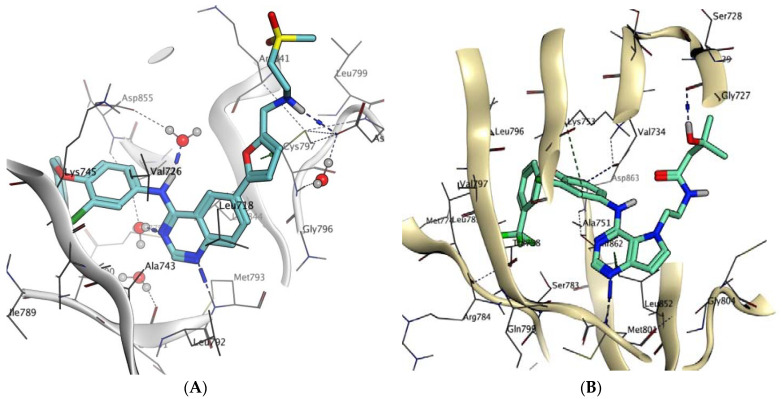
(**A**) 3D of Lapatinib interactions in the active binding site of EGFR (1XKK); (**B**) 3D of TAK285 interactions in the active binding site of HER2 (3RCD)**.**

The utilization of molecular docking was employed to evaluate the interaction between the hybrid compounds that were generated and the kinase domain of EGFR/HER2. The objective of this investigation was to offer a credible rationale for the biological activity of the compounds and to reveal their probable binding pattern. The study was conducted with the MOE 2019.01 software, specifically the molecular operating environment, employing the QuickPrep procedure. There are many crystal structures (PDB codes) of EGFR and HER2 that were obtained by downloading the PDB codes 1XKK [55] and 3RCD [56], respectively, due to their high resolution and their suitable co-crystallized ligands (Lapatinib and TAK-285, respectively). In the first stage of the study, the validation of the docking method involved the redocking of co-crystallized ligands into the active regions of EGFR and HER2. The docking position of the lapatinib derivative, 4-anilinoquinazoline, into the active region of EGFR resulted in significant interactions. Specifically, the ligand formed hydrogen bonds with the amino acids Met793, Asp800, and Leu788 (Figure 5). Furthermore, the arene-H interactions occur with the amino acids Leu844 and Leu718, and they are also facilitated by water-mediated hydrogen bonding with Thr854. Regarding HER2, TAK-285 accurately replicated the essential interactions seen between the co-crystalized ligand and the active site. Specifically, TAK-285 engaged in hydrogen bonding with Asp863 and Gly727 and formed two hydrogen bonds with Met801. In addition, there is a π-H interaction with the amino acids Phe864, Lys753, and Leu800. The minimal root-mean-square deviation (RMSD) values for co-crystallized ligand in EGFR (0.917 Å) and HER2 (0.625 Å) provide evidence for this assertion (Figure 5). The patterns of binding observed in both kinases were found to be similar for the recently synthesized thiazole-pyrazoline hybrids.

**Figure 6:**
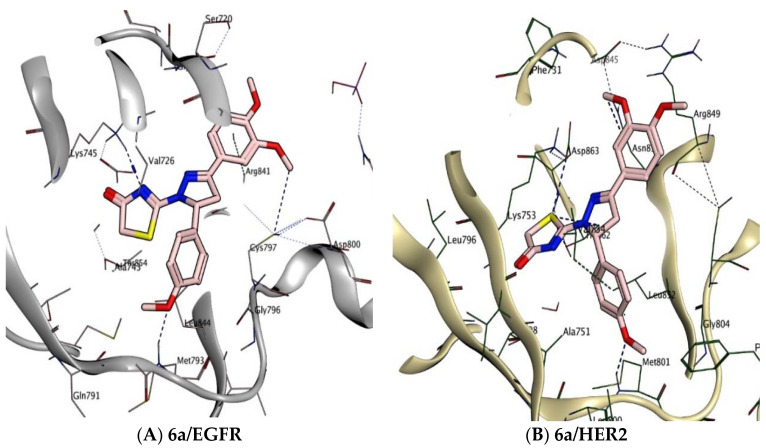
3D binding interactions of 6a in (**A**) EGFR and (**B**) HER2 binding pockets (for 2D binding interactions, see Figure S64, Supplementary Materials).

**Figure 7:**
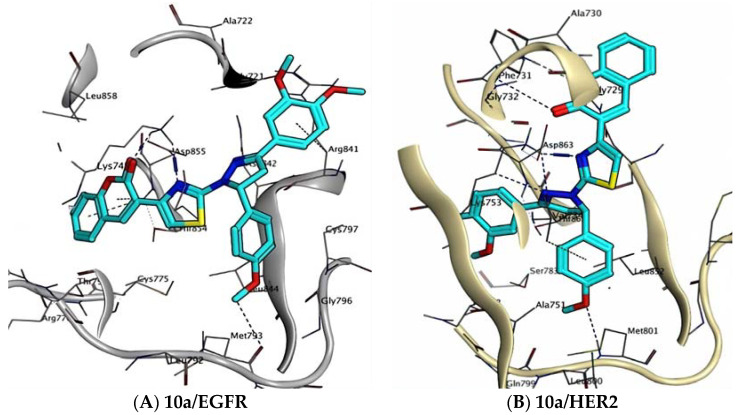
3D binding interactions of **10a** at (**A**) EGFR and (**B**) HER2 binding pockets (for 2D binding interactions, see Figure S65, Supplementary Materials).

Further investigation was conducted on the recently synthesized compounds, namely **6a** and **10a**, which have shown potential as biologically active derivatives. These compounds were subjected to a detailed analysis and compared to the inhibitors Lapatinib and TAK-285, which were docked and co-crystallized with the binding pockets of EGFR and HER2, respectively. Based on the investigation of their binding modes as depicted in Figure 6 and Figure 7.

Compound **6a** inside the EGFR binding site (S = −12.42 kcal/mol) formed H-bonds with Met793, Lys745, and Val726 and two H-bonds with Cys797 amino acids, besides two π-H bindings with Arg841. For HER2, compound **6a** (S = −10.63 kcal/mol) revealed H-bonds with Met801, Asn850, and Asp863, 2 H-bonds with Thr862 amino acids, and a π-H bond with Val734 amino acid (Figure 6).

On the other hand, compound **10a** in the EGFR binding site (S = −16.18 kcal/mol) achieved H-bonds with Met793 and Leu844, two H-bonds with Lys745 amino acids, π-H bonds with Val726, Thr854, and Arg841, and two π-H bonds with Asp855 amino acids. Furthermore, it formed H-bonds with Met801, Val734, and Phe731, three H-bonds with Lys753 amino acids besides π-H bonds with Val734, Phe731, and Gly729, and two π-H bonds with Arg849 amino acids at the HER2 binding site (S = −6.63 kcal/mol) (Figure 7).

### 2.4. In Silico Evaluation of Pharmacokinetic Parameters

The processes of absorption, distribution, metabolism, excretion, and toxicity (ADMET) are important in the prediction of oral bioavailability. The assessment and prediction of pharmacokinetic parameters ADMET by in silico methods hold significance within the pharmaceutical sector. This phenomenon occurs due to the fact that this evaluation expedites the progression of exploration and advancement of novel pharmaceutical candidate substances. This study provides an overview of the ADMET outcomes for the compounds examined. Lipinski’s rule of five predicts the potential for a tiny compound to exhibit drug-like properties. Therefore, the SwissADME model was employed to forecast the aforementioned terms for compounds **4a**–**d**, **5a**–**d**, **6a**, **b**, **7a**–**d**, **8a**, **b**, and **10a**, **b** (Table S1).

The data acquired indicate that all compounds conform to the Veber rule (Table S1) without any violations, indicating their drug-like properties. However, **4b**, **4d**, **5b**, and **5d** each exhibit one violation of the Veber rule. Compounds **6a**, **6b**, and **7a** exhibit no violations according to Lipinski’s rule. The remaining compounds demonstrate one violation, with the exception of compounds **4b**, **4c**, **5b**, and **5d**, which exhibit two violations. In addition, **4b**, **d**, **5a**–**d**, **6a**, **b**, **7a**–**d**, **8a**, **b**, and **10a** exhibited clogP values within an acceptable range, often below or approximately 5, whereas compounds **4a**, **4c**, and **10b**. The topological polar surface area (TPSA) is a measure of the total surface area occupied by polar atoms within a molecule. The acceptable range for TPSA is often defined as 20–130 Å2.

In our study, all of the compounds analyzed were found to fall within this acceptable range. Except for compounds **5b**, **6a**, **6b**, and **8a**, all other compounds exhibit low solubility. All of the compounds did not exhibit the ability to penetrate the blood-brain barrier (BBB). P-glycoprotein (P-gp) functions as an efflux transporter responsible for the extrusion of xenobiotics from cells, hence facilitating their clearance. The compounds **4a**, **4c**, **5a**, **7b**, **8a**, and **8b** were determined to be non-substrates for P-gp, while all other compounds were anticipated to have this characteristic. The bioavailability scores for all compounds, with the exception of compounds **4a**, **4c**, **5b**, and **5d**, were 0.55. However, these specific compounds exhibited a bioavailability score of 0.17. Except for compounds **4a**–**d**, **5b**, **5d**, and **10a**, **b**, all other compounds exhibit favorable gastrointestinal (GI) absorption. Compounds **6a**, **b** exhibit no pain or Brenk alerts, whereas compounds **10a**, **b** have no pain alerts and just one Brenk warning. The remaining compounds show one pain alert and one Brenk alert (Table S1, Supplementary Materials).

The Swiss ADME platform offers a BOILED-Egg intrinsic model that can be utilized for the prediction of BBB permeability and passive gastrointestinal absorption (HIA) [57].

**Figure 8:**
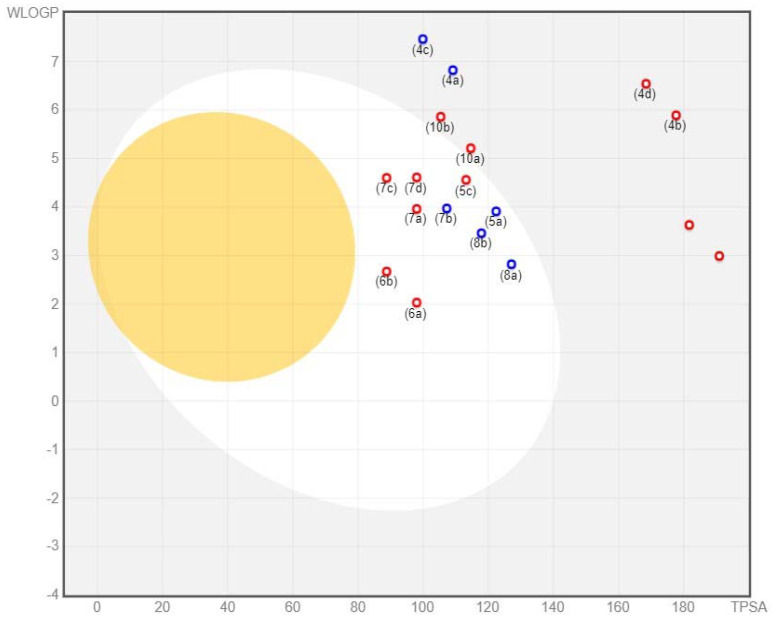
BOILED–Egg plot for **4a**–**d**, **5a**–**d**, **6a**, **b**, **7a**–**d**, **8a**, **b**, and **10a**, **b**.

Figure 8 depicts the distribution of **4a**–**d**, **5a**–**d**, **6a**, **b**, **7a**–**d**, **8a**, **b** and **10a**, **b** on a boiled egg. No compound was detected within the yellow zone, suggesting that all compounds do not possess the ability to penetrate the BBB. The proper absorption of compounds **5a**, **5c**, **6a**, **b**, **7a**–**d**, and **8a**, **b** was suggested by their presence in the white zone. Conversely, compounds **4a**–**d**, **5b**, **5d**, and **10a**, **b** were found outside the white region, suggesting poor gastrointestinal absorption. Out of the total chemicals examined, six were predicted to exhibit P-glycoprotein substrate activity (PGP+), as indicated by the blue dot, but the remainder of the compounds did not demonstrate interaction with the active efflux P-glycoprotein pump (PGP-), as represented by the red dot.

## 3. Conclusions

A series of thiazolyl–pyrazolines was designed and synthesized. Compounds **6a**, **b**, and **10a**, **b** possessed potent anticancer activities toward MCF-7 and served as dual inhibitors for EGFR/HER2. Among the screened compounds, four compounds (**6a**, **6b**, **10a**, and **10b**) revealed significant anticancer activity with IC_50_ = 4.08, 5.64, 3.37, and 3.54 µM against MCF-7 when compared to Lapatinib (IC_50_ = 5.88 µM). Compounds **6a** and **10a** showed IC_50_ = 0.024 and 0.005 µM, respectively, toward EGFR and IC_50_ = 0.047 and 0.022 µM, respectively, toward HER2. **6a** exhibited high cell accumulation for the MCF-7 cell line at G0-G1, whereas **10a** arrests the cell cycle at the G1 and S phases. Furthermore, compounds **6a** and **10a** showed significant total apoptotic death. Finally, the molecular modeling studies investigated the mode of binding and potential variations in interactions between the designed derivatives and the EGFR and HER2 active sites. Thus, the thiazolyl-pyrazoline scaffold presents an interesting avenue for the discovery of more potent dual EGFR/HER2 inhibitors in the future.

## 4. Experimental

### 4.1. Chemistry

For chemistry apparatus, see the supplementary file (page 65) Carbothioamides **1a**, **b** [40,43], *N*-arylpropanehydrazonoyl chlorides **2a**–**d** [44], and 2-bromoacetyl coumarin **9** [45] were prepared following the reported methods.

#### 4.1.1. Synthesis of the 5-(aryldiazinyl)-4-methylthiazoles 4a–d and 5-(arylhydrazineylidene)-thiazol-4(5*H*)-ones 5a–d

Carbothioamide derivatives **1a**, **b** (1 mmol) and hydrazonyl chlorides **2a**–**d** (1 mmol) in absolute EtOH (40 mL) were refluxed for 6 h. The formed precipitate was filtered, washed with EtOH, and crystallized from EtOH/DMF to yield the 5-(aryldiazinyl)-4-methylthiazoles 4a–d and 5-(arylhydrazineylidene)-thiazol-4(5*H*)-ones 5a–d.

5-((4-Chlorophenyl)diazenyl)-2-(3-(3,4-dimethoxyphenyl)-5-(4-methoxyphenyl)-4,5-dihydro-1*H*-pyrazol-1-yl)-4-methylthiazole (4a): Reddish orange powder, 59% yield; mp 192–194 °C; IR *ν*_max_/cm^−1^ 3061 (C-H Ar), 2967 (C-H Ali.), 1566 (C=N), 1508 (C=C), 1244 (C-O); ^1^H-NMR *δ* 2.48 (s, 3H, CH_3_), 3.38 (dd, *J* = 18, 4.8 Hz, 1H, H_a_4 pyrazoline), 3.73 (s, 3H, OCH_3_), 3.83 (s, 3H, OCH_3_), 3.85 (s, 3H, OCH_3_), 4.01 (dd, *J* = 18, 11.2 Hz, 1H, H_b_4 pyrazoline), 5.78 (dd, *J* = 11.2, 4.8 Hz, 1H, H5 pyrazoline), 6.92 (d, *J* = 8.8 Hz, 2H, ArHs), 7.04 (d, *J* = 8.4 Hz, 1H, ArH), 7.21 (d, *J* = 8.5 Hz, 2H, ArHs), 7.33-7.40 (m, 2H, ArHs), 7.49 (d, *J* = 8.7 Hz, 2H, ArHs), 7.66 (d, *J* = 8.7 Hz, 2H, ArHs); ^13^C-NMR *δ* 16.55 (C of CH_3_), 44.01 (C4 of pyrazoline), 55.57 (C of OCH_3_), 56.05 (C of OCH_3_), 56.14 (C of OCH_3_), 62.95 (C5 of pyrazoline), 109.70, 112.04, 114.67 (2C), 121.63, 123.24, 123.49 (2C), 127.61 (2C), 129.78 (2C), 133.33, 133.45, 140.60, 149.34, 151.52, 151.90, 157.79, 159.22, 159.91, 165.00 (C2 of thiazole); MS *m*/*z* (*%*) 548.88 (M^+^), 550.3 (M^+^+1); For C_28_H_26_ClN_5_O_3_S (548.06): Calc.: C, 61.36; H, 4.78; N, 12.78. Found: C, 61.62; H, 4.59; N, 12.97.
4-((2-(3-(3,4-Dimethoxyphenyl)-5-(4-methoxyphenyl)-4,5-dihydro-1*H*-pyrazol-1-yl)-4-methylthiazol-5-yl)diazenyl)benzenesulfonamide (**4b**): Red powder, 52% yield; mp 200–202 °C; IR *ν*_max_/cm^−1^ 3342, 3241 (NH_2_), 2935 (C-H Ali.), 1554 (C=N), 1509 (C=C), 1245 (C-O); ^1^H-NMR *δ* 2.53 (s, 3H, CH_3_), 3.43 (dd, *J* = 18, 6.4 Hz, 1H, H_a_4 pyrazoline), 3.73 (s, 3H, OCH_3_), 3.83 (s, 3H, OCH_3_), 3.85 (s, 3H, OCH_3_), 4.05 (dd, *J* = 18, 12 Hz, 1H, H_b_4 pyrazoline), 5.83 (dd, *J* = 11.2, 4.0 Hz, 1H, H5 pyrazoline), 6.93 (d, *J* = 8.8 Hz, 2H, ArHs), 7.06 (d, *J* = 8.4 Hz, 1H, ArH), 7.21 (d, *J* = 8.7 Hz, 2H, ArHs), 7.64-7.33 (m, 4H, 2ArH + 2H of D_2_O exchangeable NH_2_), 7.79 (d, *J* = 8.7 Hz, 2H, ArHs), 7.89 (d, *J* = 8.7 Hz, 2H, ArHs); ^13^C-NMR *δ* 16.25 (C of CH_3_), 43.56 (C4 of pyrazoline), 55.10 (C of OCH_3_), 55.57 (C of OCH_3_), 55.68 (C of OCH_3_), 62.55 (C5 of pyrazoline), 109.25, 111.54, 114.21 (2C), 121.28, 121.59 (2C), 122.68, 126.94, 127.15 (2C), 132.89, 140.38, 143.04, 148.88, 151.53, 154.22, 157.93, 158.78, 161.17, 162.31, 165.01 (C2 of thiazole); MS *m*/*z* (*%*) 592.98 (M^+^). For C_28_H_28_N_6_O_5_S_2_ (592.69): Calc.: C, 56.74; H, 4.76; N, 14.18. Found: C, 56.89; H, 4.92; N, 14.31.
2-(5-(4-Chlorophenyl)-3-(3,4-dimethoxyphenyl)-4,5-dihydro-1*H*-pyrazol-1-yl)-5-((4-chlorophenyl)diazenyl)-4-methylthiazole (**4c**): Redish orange powder, 91% yield; mp 210–212 °C; IR *ν*_max_/cm^−1^ 3070 (C-H Ar), 2963 (C-H Ali.), 1556 (C=N), 1509 (C=C), 1246 (C-O); ^1^H-NMR *δ* 2.5 (s, 3H, CH_3_), 3.43 (dd, *J* = 18, 3.6 Hz, 1H, H_a_4 pyrazoline), 3.82 (s, 3H, OCH_3_), 3.84 (s, 3H, OCH_3_), 4.07 (dd, *J* = 18, 12 Hz, 1H, H_b_4 pyrazoline), 5.87 (dd, *J* = 12, 4.0 Hz, 1H, H5 pyrazoline), 7.06 (d, *J* = 8 Hz, 1H, ArHs), 7.30–7.52 (m, 8H, ArHs), 7.66 (d, *J* = 8 Hz, 2H, ArHs); ^13^C-NMR *δ* 16.1 (C of CH_3_), 43.48 (C4 of pyrazoline), 55.60 (C of OCH_3_), 55.71 (C of OCH_3_), 62.28 (C5 of pyrazoline), 109.28, 111.57, 121.22, 122.63, 123.10 (2C), 127.82 (2C), 128.90 (2C), 129.36 (2C), 132.27, 132.66, 140.03, 140.32, 148.88, 151.03, 151.48, 157.26, 159.31, 164.53 (C2 of thiazole); MS *m*/*z* (*%*) 554.86 (M^+^+2). For C_27_H_23_Cl_2_N_5_O_2_S (552.47): Calc.: C, 58.70; H, 4.20; N, 12.68. Found: C, 58.88; H, 4.35; N, 12.79.
4-((2-(5-(4-Chlorophenyl)-3-(3,4-dimethoxyphenyl)-4,5-dihydro-1*H*-pyrazol-1-yl)-4- methylthiazol-5-yl)diazenyl)benzenesulfonamide (**4d**): Red powder, 90% yield; mp 208–210 °C; IR *ν*_max_/cm^−1^ 3314, 3233 (NH_2_), 3084 (C-H Ar), 2958 (C-H Ali.), 1600 (C=N), 1509 (C=C), 1250 (C-O); ^1^H-NMR *δ* 2.52 (s, 3H, CH_3_), 3.45 (dd, *J* = 18, 4 Hz, 1H, H_a_4 pyrazoline), 3.83 (s, 3H, OCH_3_), 3.85 (s, 3H, OCH_3_), 4.09 (dd, *J* = 18, 11.4 Hz, 1H, H_b_4 pyrazoline), 5.9 (dd, *J* = 11.4, 4.0 Hz, 1H, H5 pyrazoline), 7.06 (d, *J* = 8.4 Hz, 1H, ArH), 7.31 (d, *J* = 8.4 Hz, 2H, ArHs), 7.49-7.34 (m, 6H, 4ArHs + 2H of D_2_O exchangeable NH_2_), 7.80 (d, *J* = 8.5 Hz, 2H, ArHs), 7.89 (d, *J* = 8.7 Hz, 2H, ArHs); ^13^C-NMR *δ* 16.24 (C of CH_3_), 43.49 (C4 of pyrazoline), 55.60 (C of OCH_3_), 55.72 (C of OCH_3_), 62.63 (C5 of pyrazoline), 109.33, 111.59, 121.35, 121.66 (2C), 122.54, 126.98 (2C), 127.82 (2C), 128.93 (2C), 132.33, 140.55, 148.89, 151.59, 154.17, 157.83, 160.84, 162.35 (2C), 165.00 (C2 of thiazole); MS *m*/*z* (*%*) 597.83 (M^+^); For C_27_H_25_ClN_6_O_4_S_2_ (597.11): Calc.: C, 54.31; H, 4.22; N, 14.07. Found: C, 54.49; H, 4.34; N, 14.21.
5-(2-(4-Chlorophenyl)hydrazineylidene)-2-(3-(3,4-dimethoxyphenyl)-5-(4-methoxyphenyl)-4,5-dihydro-1*H*-pyrazol-1-yl)thiazol-4(5*H*)-one (**5a**): yellow powder, 32% yield; mp > 300 °C; IR *ν*_max_/cm^−1^ 3077 (N-H), 3001 (C-H Ar), 2962 (C-H Ali.), 1668 (C=O), 1602 (C=N), 1512 (C=C); ^1^H-NMR *δ* 3.49 (dd, *J* = 18, 3.8 Hz, 1H, H_a_4 pyrazoline), 3.74 (s, 3H, OCH_3_), 3.85 (s, 3H, OCH_3_), 3.85 (s, 3H, OCH_3_), 4.13 (dd, *J* = 18, 12 Hz, 1H, H_b_4 pyrazoline), 5.84 (dd, *J* = 12, 3.8 Hz, 1H, H5 pyrazoline), 6.94 (d, *J* = 7.0 Hz, 2H, ArHs), 7.10–7.14 (m, 1H, ArH), 7.20 (d, *J* = 8.4 Hz, 2H, ArHs), 7.26–7.35 (m, 4H, ArHs), 7.4-7.45 (m, 2H, ArHs), 10.76 (s, D_2_O exchangeable, 1H, NH); ^13^C-NMR *δ* 44.02 (C4 of pyrazoline), 55.62 (C of OCH_3_), 56.09 (C of OCH_3_), 56.23 (C of OCH_3_), 63.42 (C5 of pyrazoline), 110.07, 112.16, 114.76 (2C), 115.85 (2C), 122.23, 122.48, 125.48, 127.63 (2C), 129.50 (2C), 131.08, 132.54, 143.37, 149.33, 152.53, 159.39, 162.02, 167.19 (C2 of thiazolone), 175.63 (C=O); MS *m*/*z* (*%*) 551.03 (M^+^+1), 550.52 (M^+^); For C_27_H_24_ClN_5_O_4_S (550.03): Calc.: C, 58.96; H, 4.40; N, 12.73. Found: C, 59.09; H, 4.28; N, 12.85.
4-(2-(2-(3-(3,4-Dimethoxyphenyl)-5-(4-methoxyphenyl)-4,5-dihydro-1*H*-pyrazol-1-yl)-4-oxothiazol-5(4*H*)-ylidene)hydrazineyl)benzenesulfonamide (**5b**): Yellow powder, 40% yield; mp 249–251 °C; IR *ν*_max_/cm^−1^ 3254 (NH_2_), 2965 (C-H Ali.), 1699 (C=O), 1598 (C=N), 1509 (C=C); ^1^H-NMR *δ* 3.51 (dd, *J* = 18, 4 Hz, 1H, H_a_4 pyrazoline), 3.74 (s, 3H, OCH_3_), 3.84 (s, 3H, OCH_3_), 3.85 (s, 3H, OCH_3_), 4.13 (dd, *J* = 18, 11 Hz, 1H, H_b_4 pyrazoline), 5.84 (dd, *J* = 11, 4 Hz, 1H, H5 pyrazoline), 6.94 (d, *J* = 8.8 Hz, 2H, ArHs), 7.05-7.27 (m, 4H, ArHs), 7.35-7.62 (m, 4H, 2ArHs + 2H of D_2_O exchangeable NH_2_), 7.40-7.45 (m, 2H, ArHs), 7.9 (s, 1H, ArH), 11.01 (s, D_2_O exchangeable, 1H, NH); ^13^C-NMR *δ* 43.57 (C4 of pyrazoline), 55.14 (C of OCH_3_), 55.60 (C of OCH_3_), 55.77 (C of OCH_3_), 63.02 (C5 of pyrazoline), 109.57, 111.67, 113.31, 114.25, 114.30, 114.32 (2C), 121.79, 122.00, 126.31, 127.21, 127.36, 132.03, 132.30, 136.38, 146.59, 148.88, 152.10, 158.94, 161.79, 166.64 (C2 of thiazolone), 174.92 (C=O); MS *m*/*z* (*%*) 594. 35 (M^+^); For C_27_H_26_N_6_O_6_S_2_ (594.66): Calc.: C, 54.53; H, 4.41; N, 14.13. Found: C, 54.68; H, 4.52; N, 14.26.
2-(5-(4-Chlorophenyl)-3-(3,4-dimethoxyphenyl)-4,5-dihydro-1*H*-pyrazol-1-yl)-5-(2-(4-chlorophenyl)hydrazineylidene)thiazol-4(*5H*)-one (**5c**): Yellow powder, 70% yield; mp 278–280 °C; IR *ν*_max_/cm^−1^ 3190 (N-H), 3065 (C-H Ar), 2930 (C-H Ali.), 1663 (C=O), 1601 (C=N), 1510 (C=C); ^1^H-NMR *δ* 3.52 (dd, *J* = 18.2, 4.5 Hz, 1H, H_a_4 pyrazoline), 3.84 (s, 3H, OCH_3_), 3.84 (s, 3H, OCH_3_), 4.14 (dd, *J* = 18.2, 11.1 Hz, 1H, H_b_4 pyrazoline), 5.9 (dd, *J* = 11.1, 4.0 Hz, 1H, H5 pyrazoline), 7.01 (d, *J* = 8.4 Hz, 1H, ArH), 7.22-7.35 (m, 6H, ArHs), 7.39-7.47 (m, 4H, ArHs), 10.58 (s, D_2_O exchangeable, 1H, NH); ^13^C-NMR *δ* 43.46 (C4 of pyrazoline), 55.58 (C of OCH_3_), 55.76 (C of OCH_3_), 62.67 (C5 of pyrazoline), 109.54, 111.64, 115.37, 121.79, 121.92, 125.03, 127.81 (2C), 128.96 (2C), 129.11, 132.53, 139.05, 148.87, 152.08, 161.34, 166.76 (C2 of thiazolone), 174.86 (C=O); MS *m*/*z* (*%*) 558.7 (M^+^ + 3), 554.39 (M^+^); For C_26_H_21_Cl_2_N_5_O_3_S (554.45): Calc.: C, 56.32; H, 3.82; N, 12.63. Found: C, 56.43; H, 3.98; N, 12.56.
4-(2-(2-(5-(4-Chlorophenyl)-3-(3,4-dimethoxyphenyl)-4,5-dihydro-1*H*-pyrazol-1-yl)-4-oxothiazol-5(4*H*)-ylidene)hydrazineyl)benzenesulfonamide (**5d**): Yellow powder, 80% yield; mp 292–294 °C; IR *ν*_max_/cm^−1^ 3236 (NH_2_), 3098 (N-H), 3065 (C-H Ar), 2965 (C-H Ali.), 1681 (C=O), 1642 (C=N), 1513 (C=C);^1^H-NMR *δ* 3.53 (dd, *J* = 18.3, 4.4 Hz, 1H, H_a_4 pyrazoline), 3.85 (s, 6H, 2OCH_3_), 4.17 (dd, *J* = 18.2, 11.2 Hz, 1H, H_b_4 pyrazoline), 5.92 (dd, *J* = 11.2, 4.4 Hz, 1H, H5 pyrazoline), 7.13 (d, *J* = 8.5 Hz, 1H, ArH), 7.21 (s, 2H, ArHs), 7.31 (d, *J* = 8.6 Hz, 2H, ArHs), 7.37-7.47 (m, 6H, ArHs + 2H of D_2_O exchangeable NH_2_), 7.75 (d, *J* = 8.9 Hz, 2H, ArHs), 11.05 (s, D_2_O exchangeable, 1H, NH); ^13^C-NMR *δ* 43.50 (C4 of pyrazoline), 55.62 (C of OCH_3_), 55.78 (C of OCH_3_), 62.76 (C5 of pyrazoline), 109.68, 111.69, 113.36 (2C), 121.81, 121.87, 127.34, 127.81 (2C), 128.97 (2C), 132.07, 132.54, 132.60, 136.41, 139.01, 146.63, 148.86, 152.12, 161.58, 167.04 (C2 of thiazolone), 174.90 (C=O); For C_26_H_23_ClN_6_O_5_S_2_ (599.08): Calc.: C, 52.13; H, 3.87; N, 14.03. Found: C, 52.30; H, 4.02; N, 14.16.

#### 4.1.2. Synthesis of 2-(3-(3,4-dimethoxyphenyl)-5-(aryl)-4,5-dihydro-1H-pyrazol-1-yl)thiazol-4(5H)-ones **6a**, **b**

A mixture of **1a**, **b** (2 mmol), bromoacetic acid (0.33 g, 2.4 mmol), and AcONa (0.4 g, 4 mmol) in glacial AcOH (5 mL) was refluxed for 8 h. Following the cooling, the solution was added to the ice–water mixture. The resulting precipitate was separated by filtration and subsequently subjected to crystallization in DCM/EtOH to give thiazolones **6a**, **b**.

2-(3-(3,4-Dimethoxyphenyl)-5-(4-methoxyphenyl)-4,5-dihydro-1*H*-pyrazol-1-yl)thiazol-4(5*H*)-one (**6a**): White powder, 77% yield; mp 200–202 °C; IR *ν*_max_/cm^−1^ 3008 (C-H Ar), 2963 (C-H Ali.), 1693 (C=O), 1620 (C=N), 1513 (C=C); ^1^H-NMR *δ* 3.42 (dd, *J* = 18.4, 4 Hz, 1H, H_a_4 pyrazoline), 3.73 (s, 3H, OCH_3_), 3.83 (s, 3H, OCH_3_), 3.84 (s, 3H, OCH_3_), 3.91 (s, 2H, thiazolone CH_2_), 4.05 (dd, *J* = 18.4, 11.2 Hz, 1H, H_b_4 pyrazoline), 5.73 (dd, *J* = 11.2, 4.0 Hz, 1H, H5 pyrazoline), 6.92 (d, *J* = 8.6 Hz, 2H, ArHs), 7.08 (d, *J* = 8.4 Hz, 1H, ArH), 7.15 (d, *J* = 8.5 Hz, 2H, ArH), 7.38–7.42 (m, 2H, ArHs); ^13^C-NMR *δ* 39.18 (C5 of thiazolone), 43.90 (C4 of pyrazoline), 55.59 (C of OCH_3_), 56.07 (C of OCH_3_), 56.18 (C of OCH_3_), 63.63 (C5 of pyrazoline), 110.14, 112.09, 114.68 (2C), 121.87, 122.74, 127.52 (2C), 132.97, 149.31, 152.27, 159.30, 160.99, 177.27 (C2 of thiazolone), 187.27 (C=O). MS *m*/*z* (*%*) 411.67 (M^+^). For C_21_H_21_N_3_O_4_S (411.48): Calc.: C, 61.30; H, 5.14; N, 10.21. Found: C, 61.48; H, 5.22; N, 10.35.
2-(5-(4-Chlorophenyl)-3-(3,4-dimethoxyphenyl)-4,5-dihydro-1*H*-pyrazol-1-yl)thiazol-4(5*H*)-one (**6b**): White powder, 83% yield; mp 208–210 °C; IR *ν*_max_/cm^−1^ 3004 (C-H Ar), 2965 (C-H Ali.), 1740 (C=O), 1599 (C=N), 1510 (C=C); ^1^H-NMR *δ* 3.45 (dd, *J* = 18, 4 Hz, 1H, H_a_4 pyrazoline), 3.82 (s, 3H, OCH_3_), 3.83 (s, 3H, OCH_3_), 3.93 (s, 2H, thiazolone CH_2_), 4.09 (dd, *J* = 18, 11.2 Hz, 1H, H_b_4 pyrazoline), 5.79 (dd, *J* = 11.2, 4.0 Hz, 1H, H5 pyrazoline), 7.09 (d, *J* = 8.4 Hz, 1H, ArH), 7.25 (d, *J* = 8.4 Hz, 2H, ArHs), 7.36–7.44 (m, 4H, ArHs); ^13^C-NMR *δ* 39.28 (C of thiazolone), 43.79 (C4 of pyrazoline), 56.05 (C of OCH_3_), 56.16 (C of OCH_3_), 63.42 (C5 of pyrazoline), 110.17, 112.04, 121.90, 122.60, 128.15 (2C), 129.35 (2C), 132.89, 139.90, 149.30, 152.31, 160.83, 177.47 (C2 of thiazolone), 187.15 (C=O); MS *m*/*z* (*%*) 417.82 (M^+^ +2), 415.78 (M^+^). For C_20_H_18_ClN_3_O_3_S (415.89): Calc.: C, 57.76; H, 4.36; N, 10.10. Found: C, 57.67; H, 4.48; N, 10.34.

#### 4.1.3. Synthesis of 5-arylidene-2-(3-(3,4-dimethoxyphenyl)-5-(4-methoxyphenyl)-4,5-dihydro-1H-pyrazol-1-yl)thiazol-4(5H)-ones **7a**–**d**

A mixture of **6a**, **b** (0.30 g, 1 mmol), the appropriate aldehyde (1.0 mmol), and piperidine (0.2 mL) in EtOH (30 mL) was refluxed for 24 h. The produced solid was filtered and crystallized from EtOH/DMF to yield the corresponding arylidines (**7a**–**d**).

5-Benzylidene-2-(3-(3,4-dimethoxyphenyl)-5-(4-methoxyphenyl)-4,5-dihydro-1*H*-pyrazol-1-yl)thiazol-4(5*H*)-one (**7a**): Yellow powder, 66% yield; mp 294–296 °C; IR *ν*_max_/cm^−1^ 3004 (C-H Ar), 2965 (C-H Ali.), 1682 (C=O), 1610 (C=N), 1511 (C=C); ^1^H-NMR *δ* 3.51 (dd, *J* = 18.2, 4 Hz, 1H, H_a_4 pyrazoline), 3.73 (s, 3H, OCH_3_), 3.85 (s, 3H, OCH_3_), 3.86 (s, 3H, OCH_3_), 4.12 (dd, *J* = 18.2, 11.2 Hz, 1H, H_b_4 pyrazoline), 5.85 (dd, *J* = 11.2, 4.0 Hz, 1H, H5 pyrazoline), 6.94 (d, *J* = 8.8 Hz, 2H, ArHs), 7.11 (d, *J* = 8.6 Hz, 1H, ArH), 7.2 (d, *J* = 8.8 Hz, 2H, ArH), 7.41 (d, *J* = 2.1 Hz, 1H, ArH), 7.44–7.76 (m, 6H, ArHs), 7.88–7.98 (m, 1H, ArH); ^13^C-NMR *δ* 43.60 (C4 of pyrazoline), 55.14 (C of OCH_3_), 55.75 (C of OCH_3_), 55.80 (C of OCH_3_), 63.32 (C5 of pyrazoline), 110.21, 111.69, 114.30 (2C), 121.67, 122.06, 127.18 (2C), 128.18, 129.29 (2C), 129.67 (2C), 129.85, 130.61, 132.17, 133.92, 148.89, 152.06, 158.94, 161.73, 169.67 (C2 of thiazolone), 179.05 (C=O); MS *m*/*z* (*%*) 499.59 (M^+^). For C_28_H_25_N_3_O_4_S (499.59): Calc.: C, 67.32; H, 5.04; N, 8.41. Found: C, 67.19; H, 5.15; N, 8.58.
2-(3-(3,4-Dimethoxyphenyl)-5-(4-methoxyphenyl)-4,5-dihydro-1*H*-pyrazol-1-yl)-5-(4-methoxybenzylidene)thiazol-4(5*H*)-one (**7b**): Orange powder, 54% yield; mp 245–247 °C; IR *ν*_max_/cm^−1^ 1682 (C=O), 1601 (C=N), 1510 (C=C); ^1^H-NMR *δ* 3.49 (dd, *J* = 18, 3.8 Hz, 1H, H_a_4 pyrazoline), 3.74 (s, 3H, OCH_3_), 3.83 (s, 3H, OCH_3_), 3.85 (s, 3H, OCH_3_), 3.86 (s, 3H, OCH_3_), 4.10 (dd, *J* = 18, 10.8 Hz, 1H, H_b_4 pyrazoline), 5.83 (dd, *J* = 10.8, 3.8 Hz, 1H, H5 pyrazoline), 6.94 (d, *J* = 8.7 Hz, 2H, ArHs), 7.09 (d, *J* = 6.4 Hz, 3H, ArHs), 7.2 (d, *J* = 8.8 Hz, 2H, ArHs), 7.41 (s, 1H, ArH), 7.47 (d, *J* = 8.3 Hz, 1H, ArH), 7.60 (d, *J* = 8.8 Hz, 3H, ArHs); ^13^C-NMR *δ* 43.57 (C4 of pyrazoline), 55.12 (C of OCH_3_), 55.43 (C of OCH_3_), 55.74 (C of OCH_3_), 55.76 (C of OCH_3_), 63.22 (C5 of pyrazoline), 110.11, 111.66, 114.28 (2C), 114.83 (2C), 121.60, 122.13, 125.23, 126.34 (2C), 127.15, 130.63, 131.57 (2C), 132.26, 148.87, 151.99, 158.91, 160.54, 161.31, 169.59 (C2 of thiazolone), 179.31 (C=O). For C_29_H_27_N_3_O_5_S (529.61): Calc.: C, 65.77; H, 5.14; N, 7.93. Found: 65.59; H, 5.31; N, 7.86.
5-Benzylidene-2-(5-(4-chlorophenyl)-3-(3,4-dimethoxyphenyl)-4,5-dihydro-1*H*-pyrazol-1-yl)thiazol-4(5*H*)-one (**7c**): Yellow powder, 78% yield; mp 283–285 °C; IR *ν*_max_/cm^−1^ 3001 (C-H Ar), 2956 (C-H Ali.), 1687 (C=O), 1609 (C=N), 1513 (C=C); ^1^H-NMR *δ* 3.54 (dd, *J* = 18, 4 Hz, 1H, H_a_4 pyrazoline), 3.85 (s, 3H, OCH_3_), 3.87 (s, 3H, OCH_3_), 4.16 (dd, *J* = 18, 11.6 Hz, 1H, H_b_4 pyrazoline), 5.93 (dd, *J* = 11.6, 4 Hz, 1H, H5 pyrazoline), 7.1–7.13 (m, 1H, ArHs), 7.24–7.58 (m, 9H, ArHs), 7.67–7.69 (m, 2H, ArHs), 7.96 (s, 1H, ArH); ^13^C-NMR *δ* 46.45 (C4 of pyrazoline), 56.07 (C of OCH_3_), 56.50 (C of OCH_3_), 63.55 (C5 of pyrazoline), 110.19, 110.73, 112.09, 122.22, 128.17 (2C), 128.28, 129.37 (2C), 129.46, 129.79 (2C), 129.80, 130.17, 131.59, 139.99, 148.84, 149.34, 152.34, 152.43, 166.19, 177.46 (C2 of thiazolone), 187.16 (C=O). MS *m*/*z* (*%*) 503.71 (M^+^). For C_27_H_22_ClN_3_O_3_S (504.00): Calc.: C, 64.34; H, 4.40; N, 8.34. Found: C, 64.61; H, 4.52; N, 8.39.
2-(5-(4-Chlorophenyl)-3-(3,4-dimethoxyphenyl)-4,5-dihydro-*1H*-pyrazol-1-yl)-5-(4-methoxybenzylidene)thiazol-4(5*H*)-one (**7d**): Yellow powder, 82% yield; m.p. 250–252 °C; IR *ν*_max_/cm^−1^ 3047 (C-H Ar), 2930 (C-H Ali.), 1688 (C=O), 1600 (C=N), 1508 (C=C); ^1^H-NMR *δ* 3.48 (dd, *J* = 18, 4 Hz, 1H, H_a_4 pyrazoline), 3.82 (s, 3H, OCH_3_), 3.83 (s, 3H, OCH_3_), 3.84 (s, 3H, OCH_3_), 4.13 (dd, *J* = 18, 11.2 Hz, 1H, H_b_4 pyrazoline), 5.89 (dd, *J* = 11.2, 4.0 Hz, 1H, H5 pyrazoline), 7.07-7.09 (m, 3H, ArHs), 7.24-7.58 (m, 9H, ArHs); ^13^C-NMR *δ* 43.50 (C4 of pyrazoline), 55.45 (C of OCH_3_), 55.59 (C of OCH_3_), 55.77 (C of OCH_3_), 62.97 (C5 of pyrazoline), 110.16, 111.67, 114.86 (2C), 121.65, 121.98, 125.11, 126.28, 127.77 (2C), 128.95 (2C), 130.90, 131.60 (2C), 132.49, 139.20, 148.85, 152.03, 160.60, 161.19, 169.81 (C2 of thiazolone), 179.22 (C=O); MS *m*/*z* (*%*) 536.57 (M^+^+1), 534.27 (M^+^). For C_28_H_24_ClN_3_O_4_S (534.03): Calc.: C, 62.98; H, 4.53; N, 7.87. Found: C, 63.23; H, 4.63; N, 7.66.

#### 4.1.4. Synthesis of 2-(3-(3,4-dimethoxyphenyl)-5-(aryl)-4,5-dihydro-1H-pyrazol-1-yl)-5-(2-oxoindolin-3-ylidene)thiazol-4(5H)-ones **8a**, **b**

A mixture of **6a**, **b** (1 mmol), isatin (0.15 g, 1 mmol), and anhydrous AcONa (0.82 g, 1 mmol) in glacial AcOH (15 mL) was refluxed for 16 h. The resulted solid was filtered and crystallized from EtOH/DMF to afford compounds **8a**, **b**.

2-(3-(3,4-Dimethoxyphenyl)-5-(4-methoxyphenyl)-4,5-dihydro-1*H*-pyrazol-1-yl)-5-(2-oxoindolin-3-ylidene)thiazol-4(5*H*)-one (**8a**): Orange powder, 84% yield; mp > 300 °C; IR *ν*_max_/cm^−1^ 3307 (N-H), 3012 (C-H Ar), 2932 (C-H Ali.), 1697 (C=O), 1613 (C=N), 1510 (C=C); ^1^H-NMR *δ* 3.51 (dd, *J* = 18, 4 Hz, 1H, H_a_4 pyrazoline), 3.73 (s, 3H, OCH_3_), 3.85 (s, 3H, OCH_3_), 3.86 (s, 3H, OCH_3_), 4.12 (dd, *J* = 18, 11 Hz, 1H, H_b_4 pyrazoline), 5.9 (dd, *J* = 11, 4.0 Hz, 1H, H5 pyrazoline), 6.91–6.95 (m, 3H, ArHs), 7.02–7.05 (m, 1H, ArH), 7.11 (d, *J* = 8.4 Hz, 1H, ArH), 7.21 (d, *J* = 8.7 Hz, 2H, ArHs), 7.31–7.41 (m, 2H, ArHs), 7.50 (d, *J* = 8.4 Hz, 1H, ArH), 8.92 (d, *J* = 7.8 Hz, 1H, ArH), 11.13 (s, D_2_O exchangeable, 1H, NH); ^13^C-NMR *δ* 43.43 (C4 of pyrazoline), 55.15 (C of OCH_3_), 55.67 (C of OCH_3_), 55.72 (C of OCH_3_), 63.35 (C5 of pyrazoline), 109.88, 110.11, 111.63, 114.30 (2C), 120.41, 121.83, 122.00, 125.80, 127.18 (2C), 128.01, 131.64, 132.19, 137.24, 143.11, 148.87, 152.13, 158.94, 162.38, 169.00 (C2 of thiazolone), 172.03, 172.20 (C=O), 178.92 (C=O); MS *m*/*z* (*%*) 540.33 (M^+^). For C_29_H_24_N_4_O_5_S (540.59): Calc.: C, 64.43; H, 4.48; N, 10.36. Found: C, 64.56; H, 4.53; N, 10.51.
2-(5-(4-Chlorophenyl)-3-(3,4-dimethoxyphenyl)-4,5-dihydro-1*H*-pyrazol-1-yl)-5-(2-oxoindolin-3-ylidene)thiazol-4(5*H*)-one (**8b**): Orange powder, 87% yield; mp > 300 °C; IR *ν*_max_/cm^−1^ 3311 (N-H), 3010 (C-H Ar), 2932 (C-H Ali.), 1681 (C=O), 1612 (C=N), 1510 (C=C); ^1^H-NMR *δ* 3.52 (d, *J* = 15.2 Hz, 1H, H_a_4 pyrazoline), 3.84 (s, 3H, OCH_3_), 3.86 (s, 3H, OCH_3_), 4.12 (d, *J* = 15.2 Hz, 1H, H_b_4 pyrazoline), 5.9 (d, *J* = 12 Hz, 1H, H5 pyrazoline), 6.93–6.95 (m, 2H, ArHs), 7.01–7.16 (m, 3H, ArHs), 7.23–7.61 (m, 5H, ArHs), 8.92 (d, *J* = 8.4 Hz, 1H, ArH), 11.18 (s, D_2_O exchangeable, 1H, NH); MS *m*/*z* (*%*) 547.03 (M^+^+1). For C_28_H_21_ClN_4_O_4_S (545.01): Calc.: C, 61.71; H, 3.88; N, 10.28. Found: C, 62.03; H, 4.13; N, 10.36.

#### 4.1.5. Synthesis of 3-(2-(3-(3,4-dimethoxyphenyl)-5-(aryl)-4,5-dihydro-1H-pyrazol-1-yl)thiazol-4-yl)-2H-chromen-2-ones **10a**, **b**

Compounds **10a**, **b** were synthesized following the same procedure for the preparation of compounds **4a**–**d** by using 3-bromoacetylcoumarin (**9**) instead of hydrazonoyl chlorides **2a**–**d**.

3-(2-(3-(3,4-Dimethoxyphenyl)-5-(4-methoxyphenyl)-4,5-dihydro-1*H*-pyrazol-1-yl)thiazol-4-yl)-2*H*-chromen-2-one (**10a**): Green powder, 53% yield; mp 228–231 °C; IR *ν*_max_/cm^−1^ 3066 (C-H Ar), 2995 (C-H Ali.), 1711 (C=O), 1605 (C=N), 1511 (C=C); ^1^H-NMR *δ* 3.42 (dd, *J* = 18, 6.8 Hz, 1H, H_a_4 pyrazoline), 3.72 (s, 3H, OCH_3_), 3.83 (s, 3H, OCH_3_), 3.85 (s, 3H, OCH_3_), 4.02 (dd, *J* = 18, 12 Hz, 1H, H_b_4 pyrazoline), 5.6 (dd, *J* = 12, 6.8 Hz, 1H, H5 pyrazoline), 6.96–7.06 (m, 4H, ArHs), 7.32–7.43 (m, 5H, ArHs), 7.60–7.70 (m, 2H, ArHs), 7.78 (d, *J* = 7.6 Hz, 1H, ArH), 8.35 (s, 1H, ArH); ^13^C-NMR *δ* 43.16 (C4 of pyrazoline), 55.06 (C of OCH_3_), 55.52 (C of OCH_3_), 55.61 (C of OCH_3_), 63.68 (C5 of pyrazoline), 108.97, 110.80, 111.55, 113.86 (2C), 115.90, 119.10, 120.23, 120.46, 123.52, 124.78, 126.60, 128.36 (2C), 128.61, 131.66, 133.56, 138.29, 143.75, 148.82, 150.68, 152.26, 153.37, 158.7 (C=O), 163.58 (C2 of thiazole); MS *m*/*z* (*%*) 539.68 (M^+^). For C_30_H_25_N_3_O_5_S (539.61): Calc.: C, 66.78; H, 4.67; N, 7.79. Found: C, 66.53; H, 4.78; N, 7.85.
3-(2-(5-(4-Chlorophenyl)-3-(3,4-dimethoxyphenyl)-4,5-dihydro-1*H*-pyrazol-1-yl)thiazol-4-yl)-2*H*-chromen-2-one (**10b**): Green powder, 69% yield; mp 238–240 °C; IR *ν*_max_/cm^−1^ 3066 (C-H Ar), 2987 (C-H Ali.), 1731 (C=O), 1603 (C=N), 1543 (C=C); ^1^H-NMR *δ* 3.34 (s, 1H, H_a_4 pyrazoline), 3.92 (s, 3H, OCH_3_), 3.96 (s,1H, H_b_4 pyrazoline), 4.01 (s, 3H, OCH_3_), 5.56 (s, 1H, H5 pyrazoline), 6.86 (d, *J* = 8.4 Hz, 1H, ArH), 7.14 (d, *J* = 8 Hz, 1H, ArH), 7.27–7.3 (m, 2H, ArHs), 7.35–7.40 (m, 4H, ArHs), 7.45–7.6 (m, 3H, ArHs), 7.82 (s, 1H, ArH), 8.47 (s, 1H, ArH); ^13^C-NMR *δ* 44.68 (C4 of pyrazoline), 56.13 (C of OCH_3_), 56.47 (C of OCH_3_), 64.51 (C5 of pyrazoline), 109.01, 110.72, 116.30 (2C), 119.62, 119.75, 120.98, 123.44, 124.65 (3C), 128.24 (3C), 128.75, 129.27 (2C), 131.48, 134.13, 139.45, 149.43, 151.60, 152.95, 159.80 (C=O), 164.00 (C2 of thiazole); MS *m*/*z* (*%*) 545.59 (M^+^+1), 544.19 (M^+^). For C_29_H_22_ClN_3_O_4_S (544.02): Calc.: C, 64.03; H, 4.08; N, 7.72. Found: C, 64.24; H, 3.98; N, 7.86.

### 4.2. Biological Evaluation

#### 4.2.1. Cytotoxicity

Anti-proliferative activities were done using the MTT colorimetric assay [58]. Cell viability was assessed after 48 h, and the viability was calculated relative to the control [59] (supplementary materials, page 66).

#### 4.2.2. EGFR and HER2 Enzyme Inhibition

Four compounds (**6a**, **6b**, **10a**, and **10b**) with the highest cytotoxic activities were evaluated against the EGFR and HER2 using lapatinib as the reference drug [60] (supplementary materials, page 66).

#### 4.2.3. Cell Cycle Analysis and Apoptotic Assay

Cell cycle analysis and apoptotic assays for **6a** and **10a** were done according to the reported procedure [61,62] (supplementary materials, page 66).

### 4.3. Computational Studies

#### 4.3.1. Molecular Modeling

The MOE (2019.0102) program was utilized for the molecular docking simulations (supplementary materials, page 67).

#### 4.3.2. In Silico SwissADME Predictions

SwissADME [63] is an online utility that is widely recognized for its reliability and availability for free (supplementary materials, page 67).

## Figures and Tables

**Table 1:** Anticancer activity (IC_50_) of the new compounds toward MCF-7.

Compound	IC_50_ (µM)
**4a**	18.55 ± 0.19
**4b**	17.12 ± 0.15
**4c**	18.74 ± 0.22
**4d**	17.08 ± 0.21
**5a**	30.24 ± 0.24
**5b**	27.31 ± 0.26
**5c**	28.03 ± 0.25
**5d**	25.70 ± 0.12
**6a**	4.08 ± 0.08
**6b**	5.64 ± 0.11
**7a**	17.79 ± 0.14
**7b**	20.03 ± 0.22
**7c**	21.16 ± 0.24
**7d**	20.17 ± 0.19
**8a**	21.33 ± 0.21
**8b**	19.46 ± 0.27
**10a**	3.37 ± 0.13
**10b**	3.54 ± 0.07
Lapatinib	5.88 ± 0.04

**Table 2:** IC_50_ values of the tested compounds toward MCF-10A.

Compound	IC_50_ (µM)
**6a**	≥50
**6b**	32.81 ± 1.27
**10a**	≥50
**10b**	27.14 ± 1.98
Lapatinib	≥50

**Table 3:** Inhibition activities as IC_50_ in µM of 6a, b and 10a, b toward EGFR and HER2**.**

Compound	IC_50_ (µM)	
	EGFR Kinase	HER2 Kinase
**6a**	0.024 ± 0.005	0.047 ± 0.008
**6b**	0.026 ± 0.002	0.081 ± 0.006
**10a**	0.005 ± 0.002	0.022 ± 0.005
**10b**	0.029 ± 0.004	0.065 ± 0.007
Lapatinib	0.007 ± 0.001	0.018 ± 0.001

**Table 4:** The cell cycle analysis of 6a and 10a in MCF-7**.**

Compound/MCF-7	DNA Content		
	%G0-G1	%S	%G2/M
**6a**/MCF-7	69.82	22.06	8.12
**10a**/MCF-7	66.41	29.55	4.04
Cont. MCF-7	62.59	26.33	11.08

**Table 5:** Apoptotic cell death of MCF-7 cells via 6a and 10a.

Compound/Cell Line	Apoptosis			Necrosis
	Total	Early	Late	
**6a**/MCF-7	51.03	28.17	17.98	4.88
**10a**/MCF-7	42.66	19.22	15.91	7.53
Cont. MCF-7	2.27	0.76	0.13	1.38

## Data Availability

Not applicable.

## Supplementary Materials

The following supporting information can be downloaded at: https://www.mdpi.com/article/10.3390/molecules28217455/s1, Figures S1–S38: NMR spectra; Figures S39–S55: IR spectra; Figures S56–S63: Mass spectra; Figures S64 and S65: 2D binding interactions of 6a and 10a; Table S1: SwissADME prediction.
