# Supplementary material for: New Thiazolyl-Pyrazoline Derivatives as Potential Dual EGFR/HER2 Inhibitors: Design, Synthesis, Anticancer Activity Evaluation and In Silico Study
Source: Molecules. 2023 Nov 6;28(21):7455. doi: 10.3390/molecules28217455 (PMC10647861; doi:10.3390/molecules28217455)
Supplement: Supplementary file 1 [file molecules-28-07455-s001.zip › molecules-2687531-supplementary.pdf]

Supplementary

New Thiazolyl-Pyrazoline Derivatives as Potential Dual HER2/EGFR Inhibitors: Design, Synthesis, Anticancer Activity Evaluation and In-Silico Study

Mariam M. Fakhry[1,*], Amr A. Mattar[1], Marwa Alsulaimany[2], Ebtesam M. Al-Olayan[3], Sara T. Al-Rashood[4] and Hatem A Abdel-Aziz[5,*]

[1]*Department of Pharmaceutical Chemistry, Faculty of Pharmacy, Egyptian Russian University, Badr, Egypt*

[2]*Department of Pharmacognosy & Pharmaceutical Chemistry, College of Pharmacy, Taibah University, Medina, 42353, Saudi Arabia*

[3]*Department of Zoology, College of Science, King Saud University, Riyadh, Saudi Arabia*

[4]*Department of Pharmaceutical Chemistry, College of Pharmacy, King Saud University, P.O. Box 2457, Riyadh 11451, Saudi Arabia*

[5]*Applied Organic Chemistry Department, National Research Center, Dokki, Cairo 12622, Egypt*

* Correspondence: mariam-medhat@eru.edu.eg (M.M.F.); hatem_741@yahoo.com (H.A.A.-A.)

Supplementary content

Figure S1: ^{1}H NMR of compound **4a** 1

Figure S2: ^{13}C NMR of compound **4a** 2

Figure S3: ^{1}H NMR of compound **4b** 3

Figure S4: ^{1}H NMR of compound **4b**, aliphatic region 4

Figure S5: ^{1}H NMR of compound **4b**, aromatic region 5

Figure S6: ^{13}C NMR of compound **4b** 6

Figure S7: ^{1}H NMR of compound **4c** 7

Figure S8: ^{13}C NMR of compound **4c** 8

Figure S9: ^{1}H NMR of compound **4d** 9

Figure S10: ^{13}C NMR of compound **4d** 10

Figure S11: ^{1}H NMR of compound **5a** 11

Figure S12: ^{13}C NMR of compound **5a** 12

Figure S13: ^{1}H NMR of compound **5b** 13

Figure S14: ^{13}C NMR of compound **5b** 14

Figure S15: ^{1}H NMR of compound **5c** 15

Figure S16: ^{13}C NMR of compound **5c** 16

Figure S17: ^{1}H NMR of compound **5d** 17

Figure S18: ^{13}C NMR of compound **5d** 18

Figure S19: ^{1}H NMR of compound **6a** 19

Figure S20: ^{13}C NMR of compound **6a** 20

Figure S21: ^{1H} NMR of compound **6b** 22

Figure S22: ^{13}C NMR of compound **6b** 22

Figure S23: ^{1}H NMR of compound **7a** 23

Figure S24: ^{13}C NMR of compound **7a** 24

Figure S25: ^{1}H NMR of compound **7b** 25

Figure S26: ^{13}C NMR of compound **7b** 26

Figure S27: ^{1}H NMR of compound **7c** 27

Figure S28: ^{1}H NMR of compound **7d** 28

Figure S29: ^{13}C NMR of compound **7d** 29

Figure S30: ^{1}H NMR of compound **8a** 30

Figure S31: ^{1}H NMR of compound **8a**, Aliphatic region 31

Figure S32: ^{1}H NMR of compound **8a**, aromatic region 32

Figure S33: ^{13}C NMR of compound **8a** 33

Figure S34: ^{1}H NMR of compound **8b** 34

Figure S35: ^{1}H NMR of compound **10a** 35

Figure S36: ^{13}C NMR of compound **10a** 36

Figure S37: ^{1}H NMR of compound **10b** 37

Figure S38: ^{13}C NMR of compound **10b** 38

Figure S39: IR of compound **4a** 39

Figure S40: IR of compound **4b** 40

Figure S41: IR of compound **4d** 41

Figure S42: IR of compound **5a** 42

Figure S43: IR of compound **5b** 43

Figure S44: IR of compound **5c** 44

Figure S45: IR of compound **5d** 45

Figure S46: IR of compound **6a** 46

Figure S47: IR of compound **6b** 47

Figure S48: IR of compound **7a** 48

Figure S49: IR of compound **7b** 49

Figure S50: IR of compound **7c** **50**

Figure S51: IR of compound **7d** **51**

Figure S52: IR of compound **8a** **52**

Figure S53: IR of compound **8b** **53**

Figure S54: IR of compound **10a** **54**

Figure S55: IR of compound **10b** **55**

Figure S56: Mass of compound **4a** **56**

Figure S57: Mass of compound **4c** **57**

Figure S58: Mass of compound **5c** **58**

Figure S59: Mass of compound **6b** **59**

Figure S60: Mass of compound **7a** **60**

Figure S61: Mass of compound **7d** **61**

Figure S62: Mass of compound **8b** **62**

Figure S63: Mass of compound **10b** **63**

Figure S64: 2D binding interactions of **6a** in (A) EGFR and (B) HER2 binding pockets…...…**64**

Figure S65: 2D binding interactions of **10a** in (A) EGFR and (B) HER2 binding pockets..…...**64**

Table S1: SwissADME prediction of physicochemical properties and bioavailability of the target compounds……………………………………………………………………...**65**

Chemistry apparatus ……………………………………………………...…...**65**

Experimental of biological evaluations……………………………………………………..66

Cytotoxicity……………………………………………………………………………...66

EGFR and HER2 enzyme inhibition……………………………………………………..66

Cell cycle analysis and apoptotic assay………………………………………………….66

Experimental of computational studies…………………………………………………67

Molecular modelling……………………………………………………………………67

In Silico SwissADME predictions ……………………………………………………..67

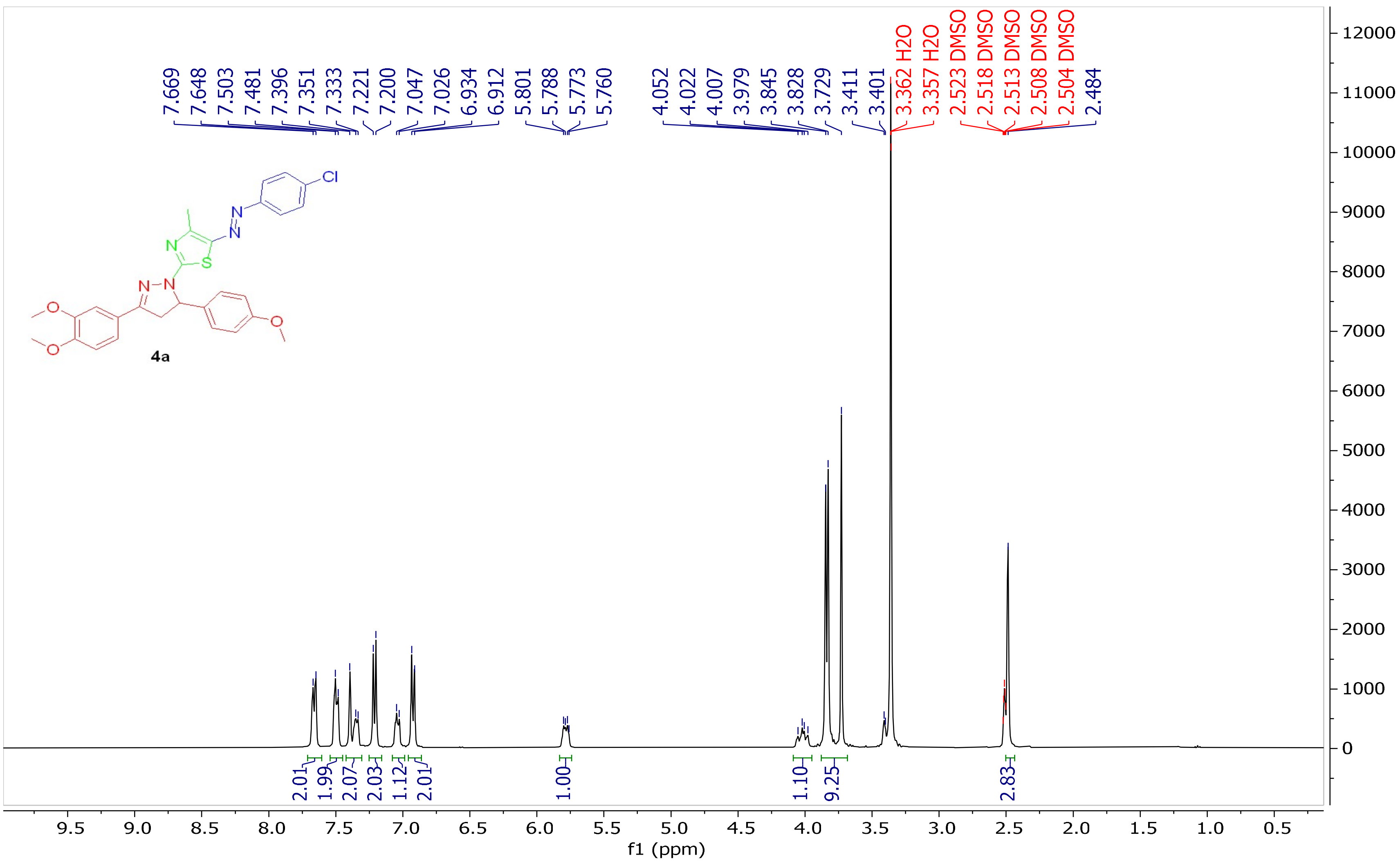

Figure S1: ^{1}H NMR of compound **4a**

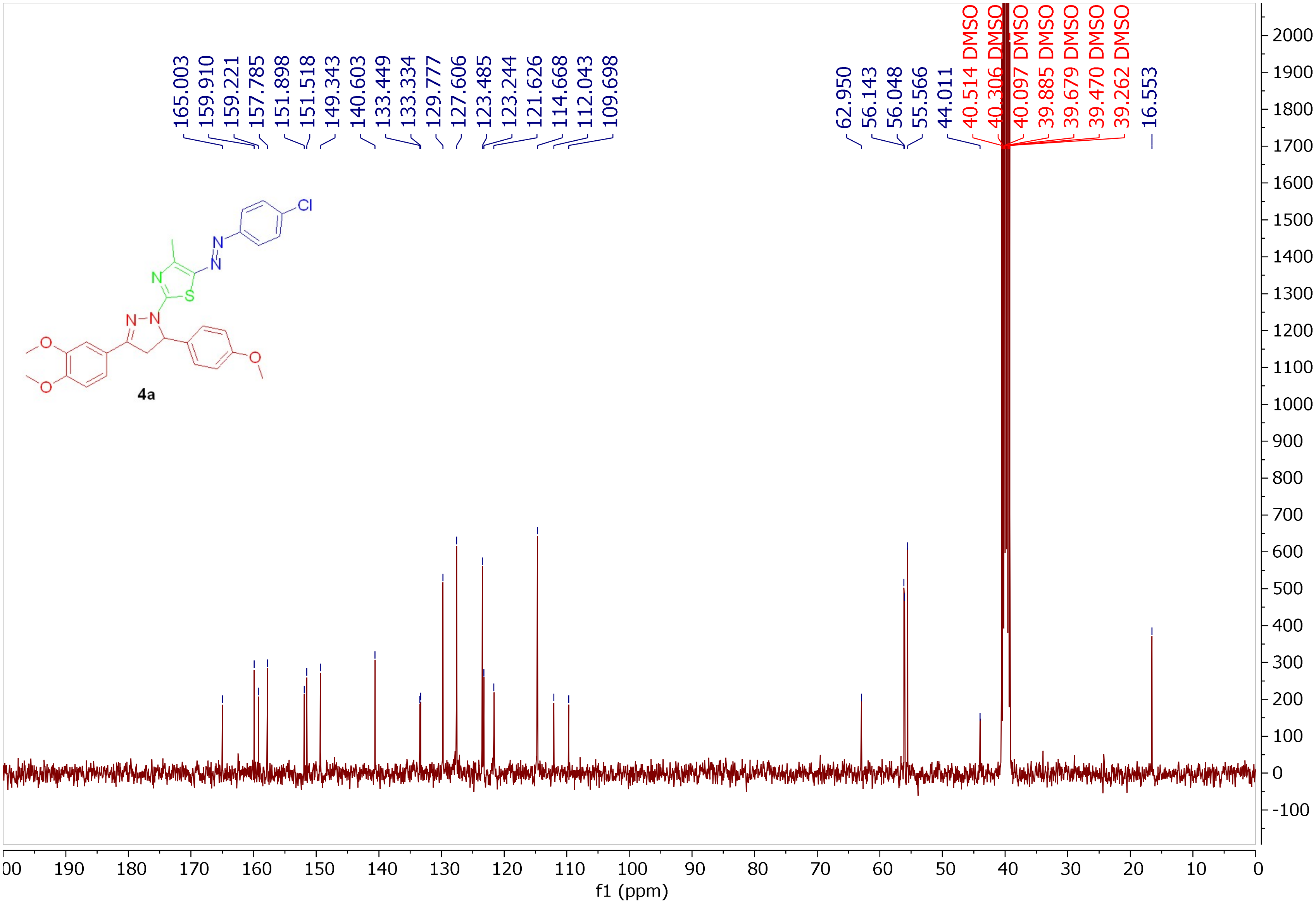

Figure S2: ^{13}C NMR of compound **4a**

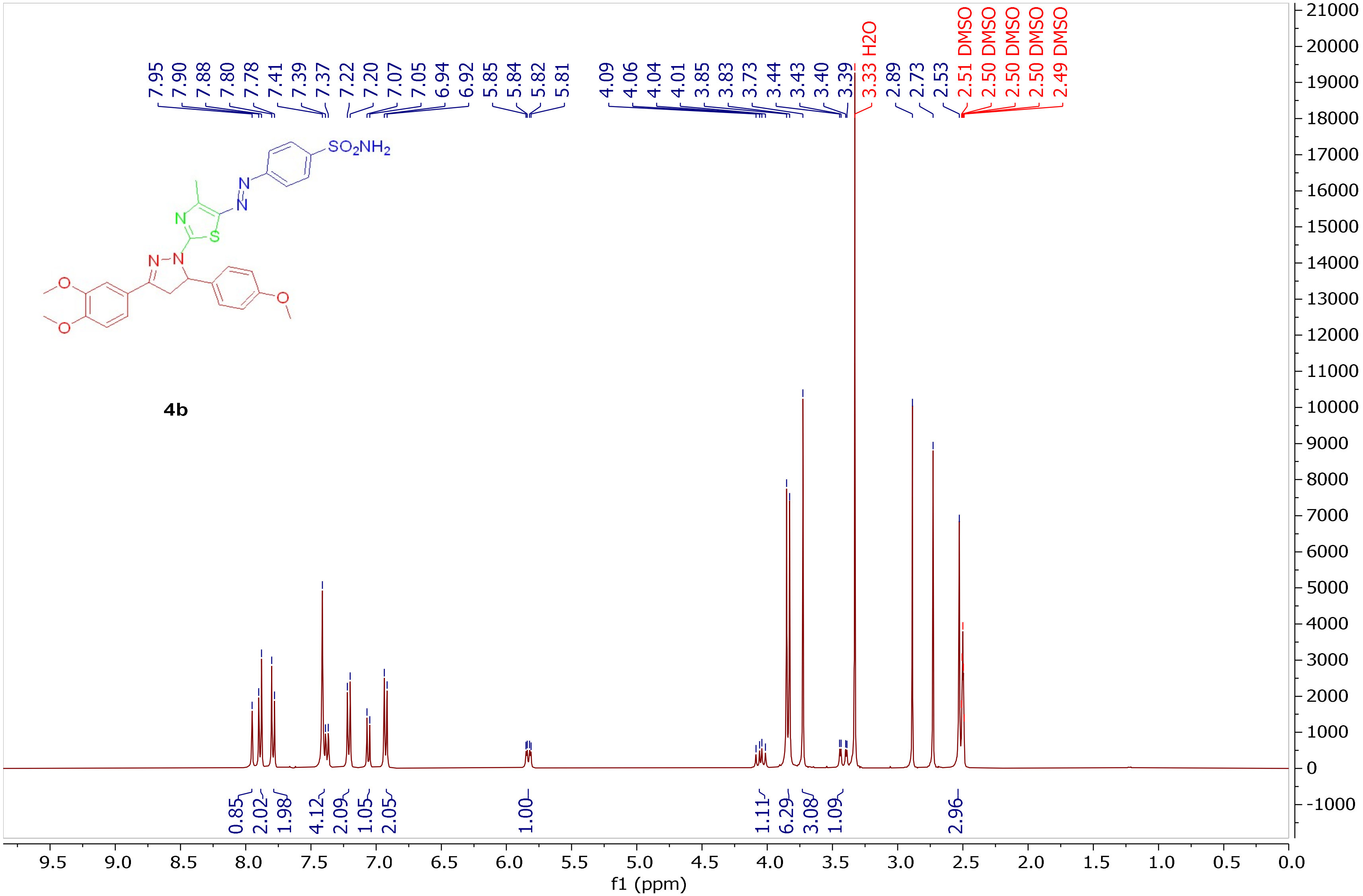

Figure S3: ^{1}H NMR of compound **4b**

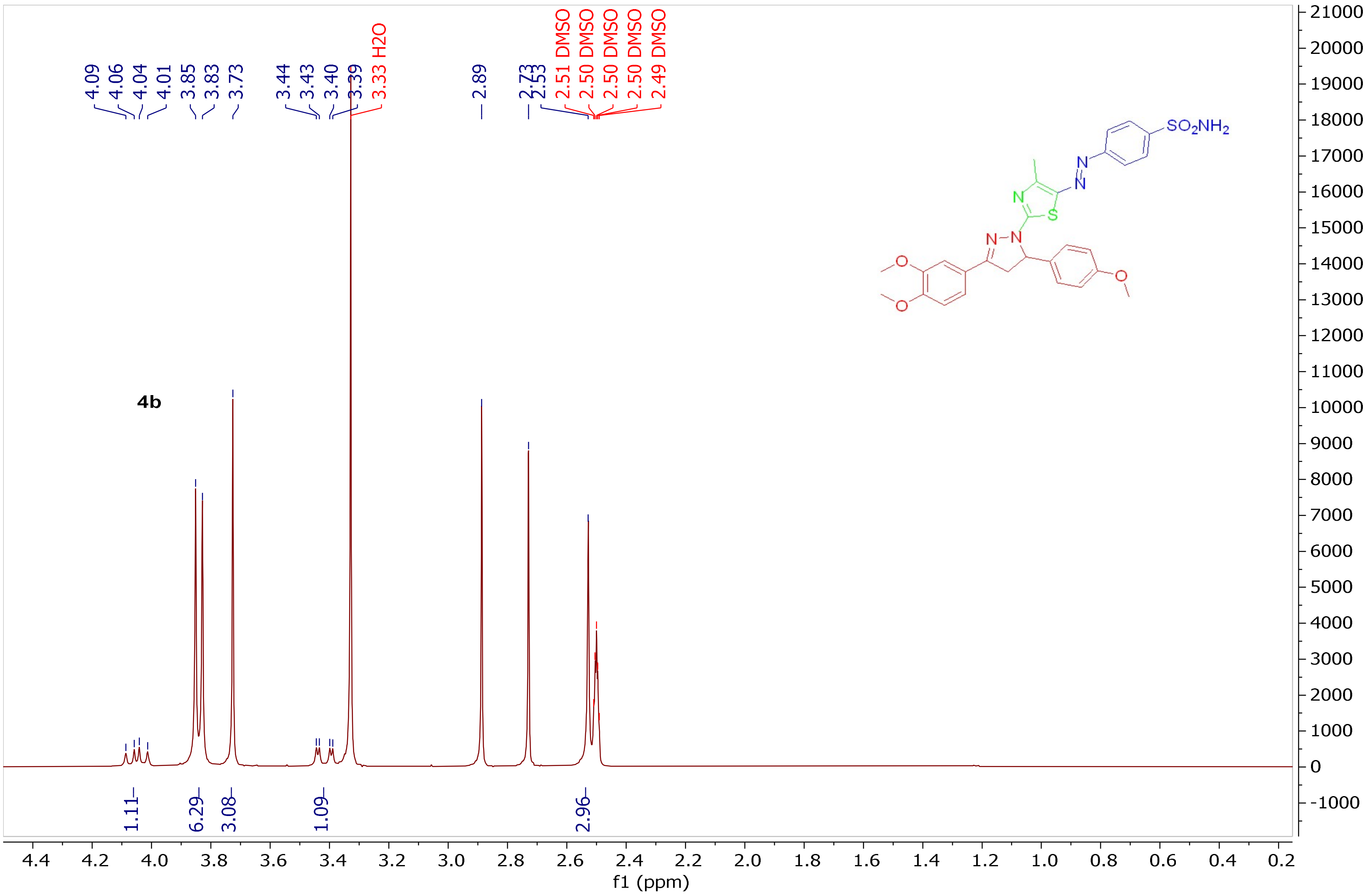

Figure S4: **^{1}H NMR** of compound **4b**, aliphatic region

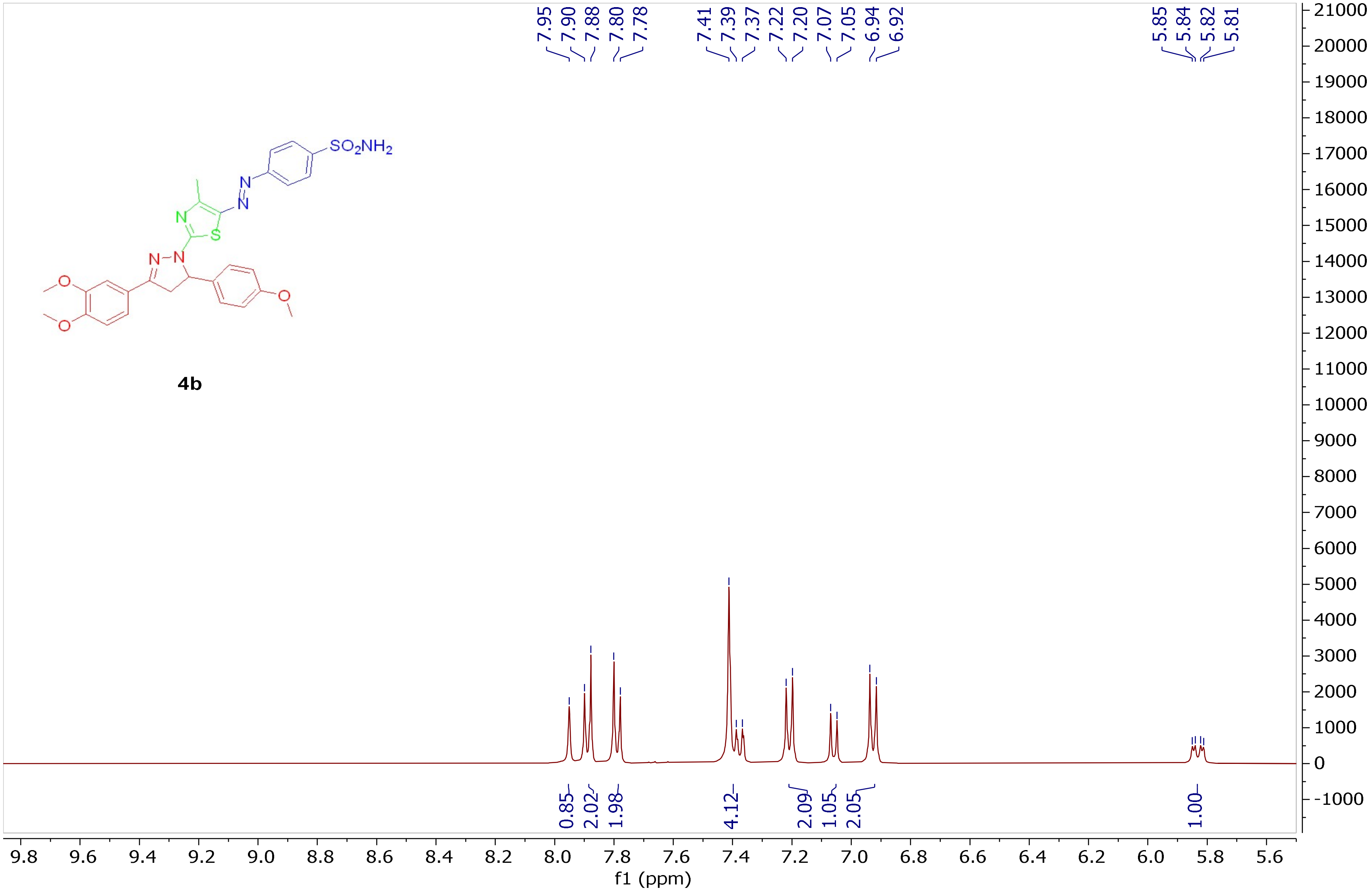

Figure S5: ^{1}H NMR of compound **4b**, aromatic region

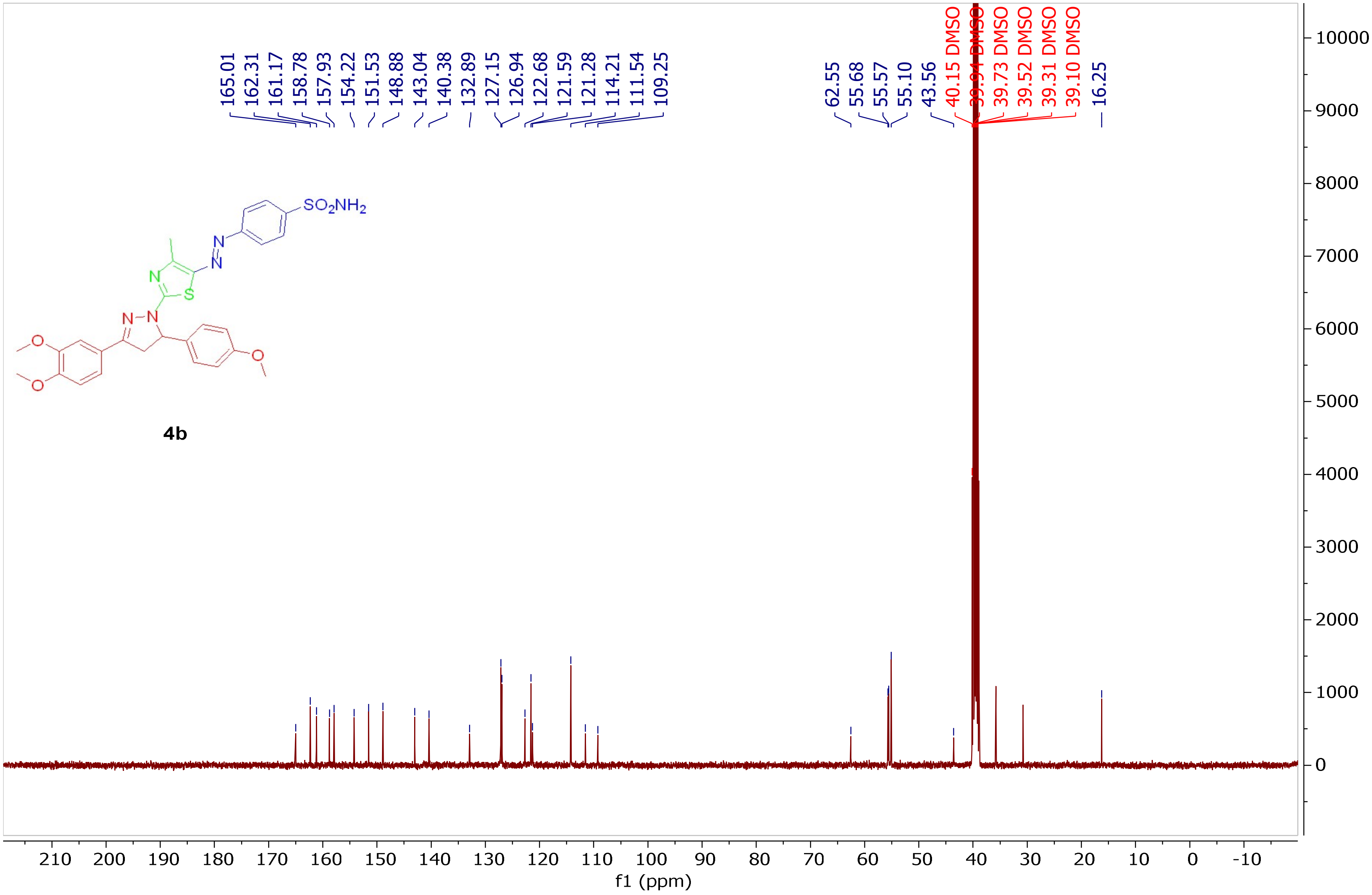

Figure S6: ^{13}C NMR of compound **4b**

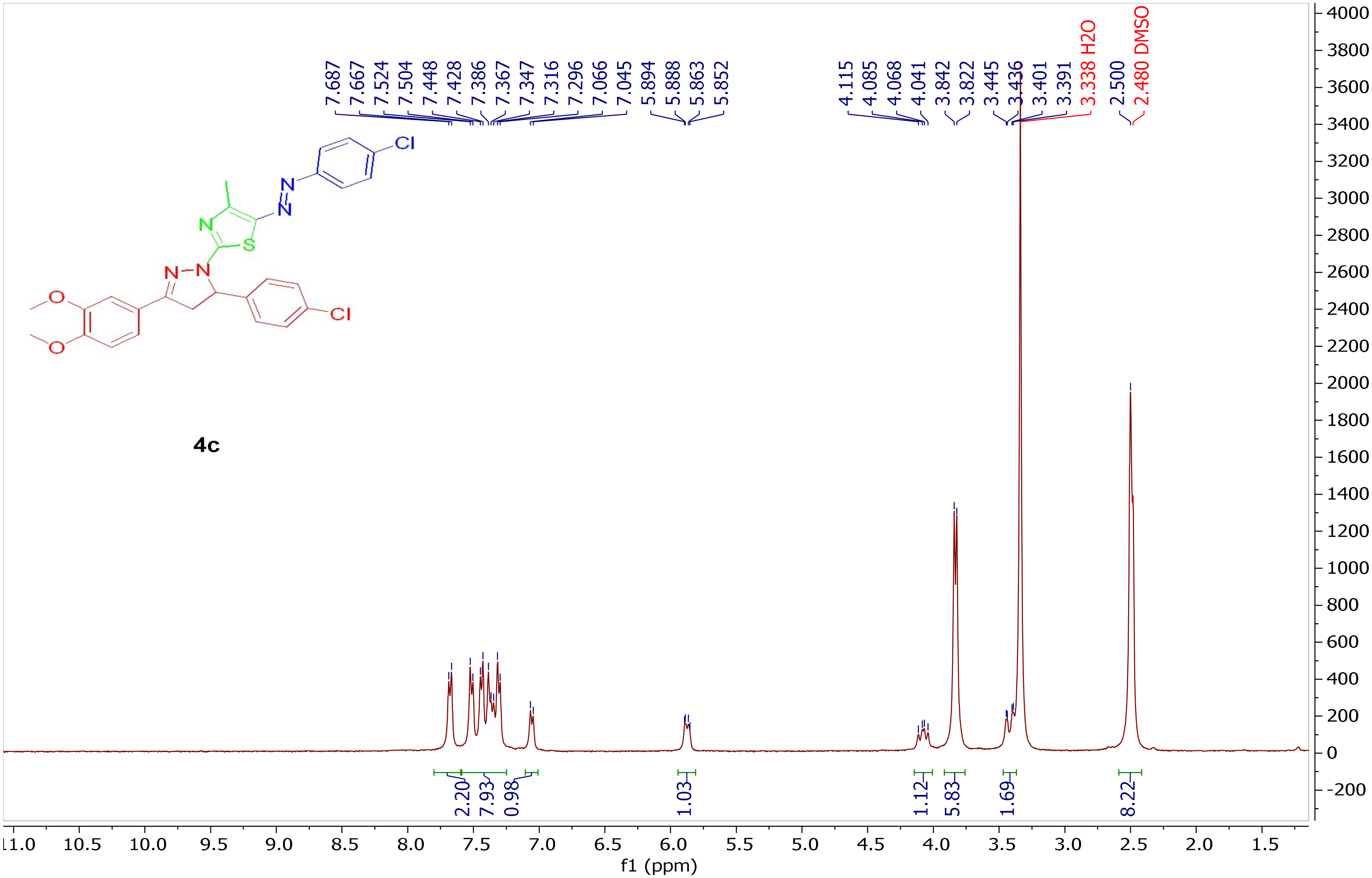

Figure S7: ^{1}H NMR of compound **4c**

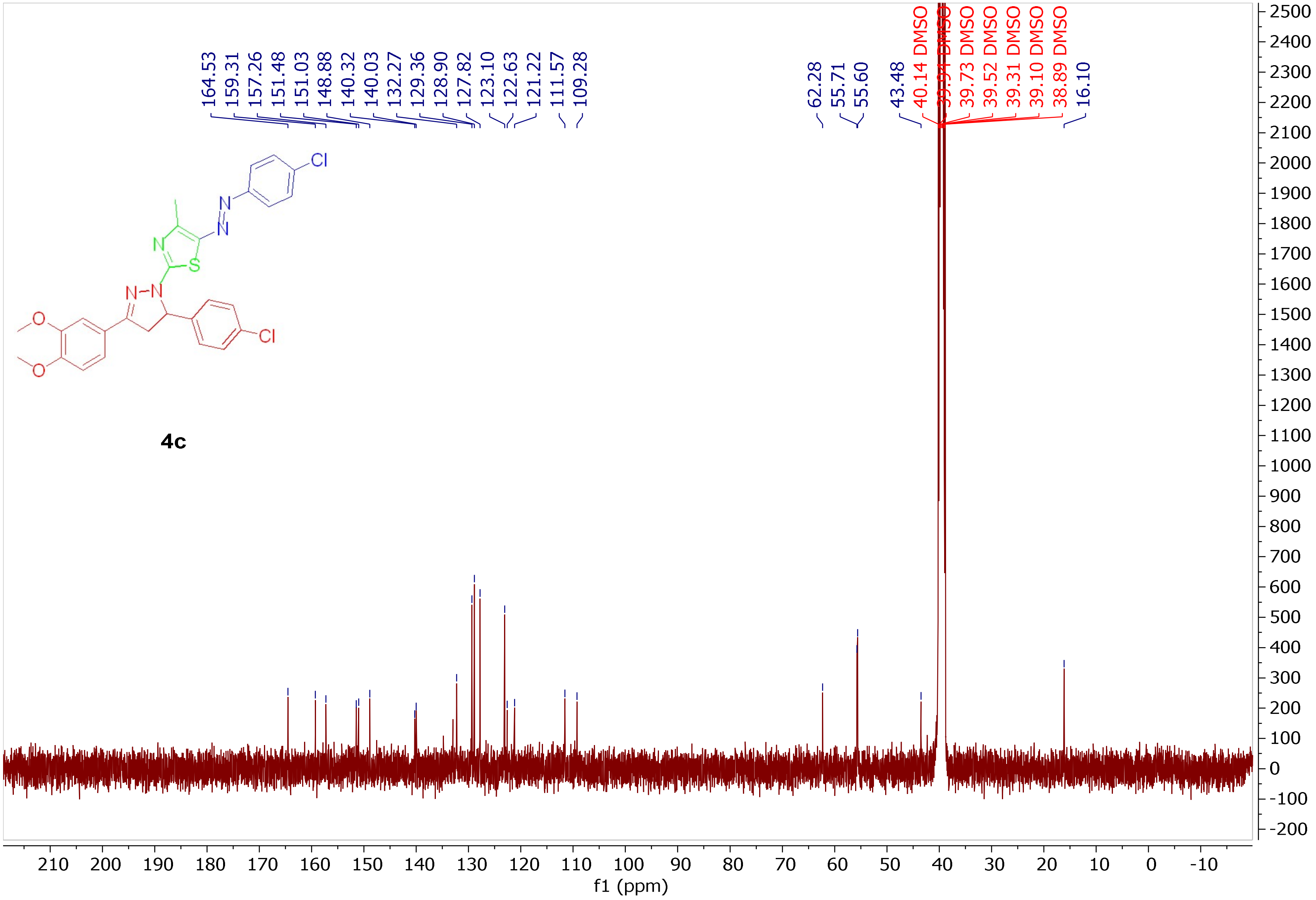

Figure S8: ^{13}C NMR of compound **4c**

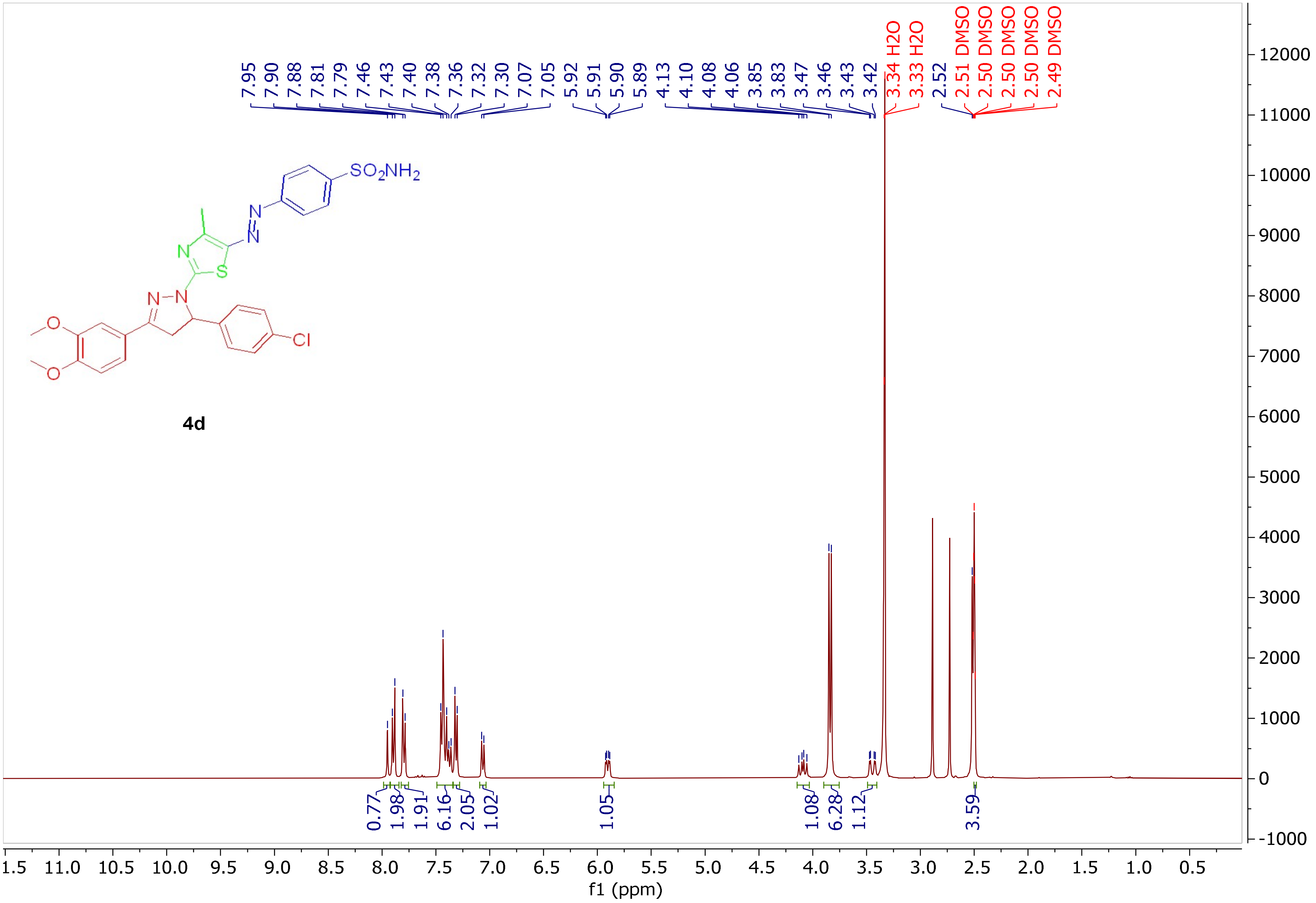

Figure S9: ^{1}H NMR of compound **4d**

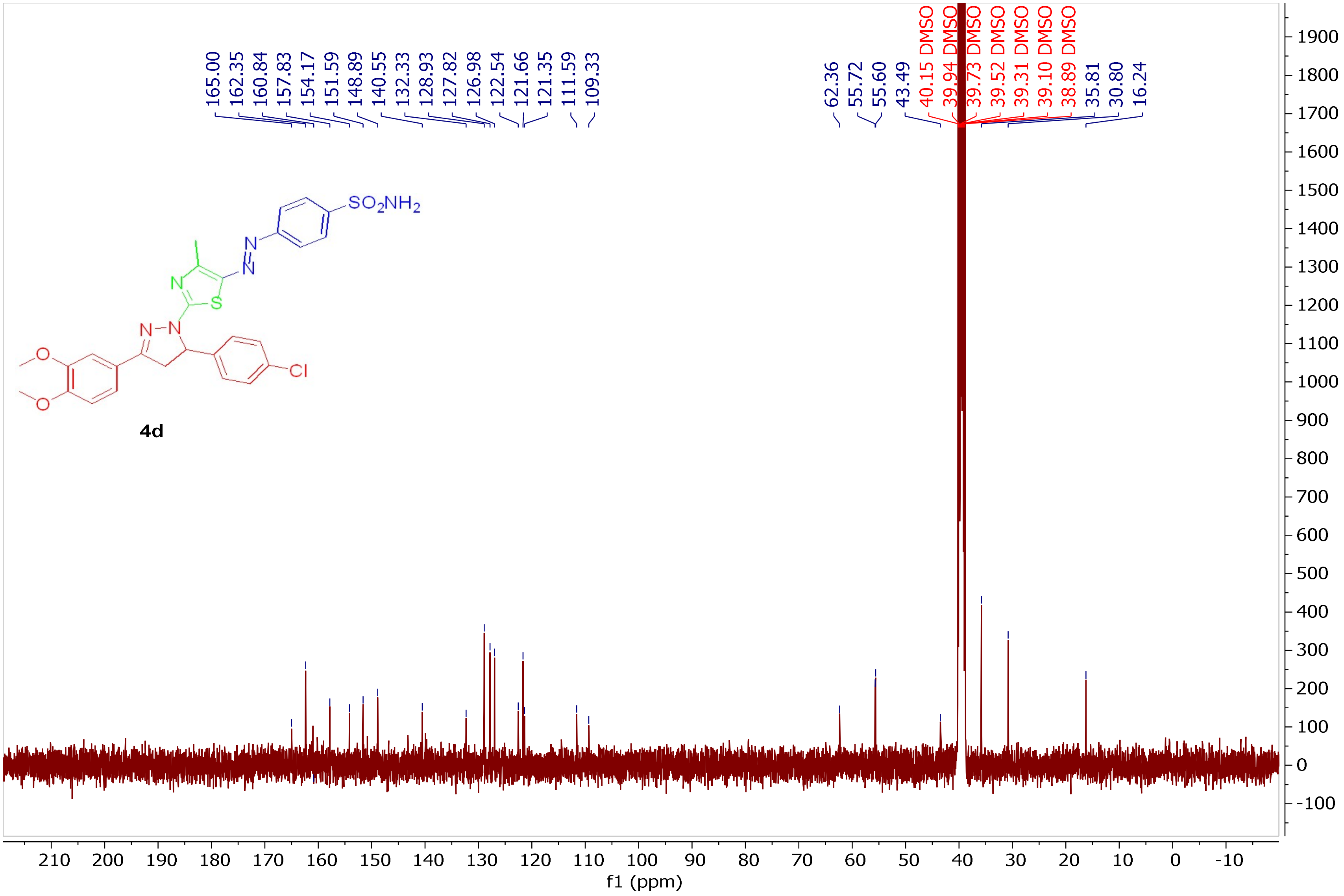

Figure S10: ^{13}C NMR of compound **4d**

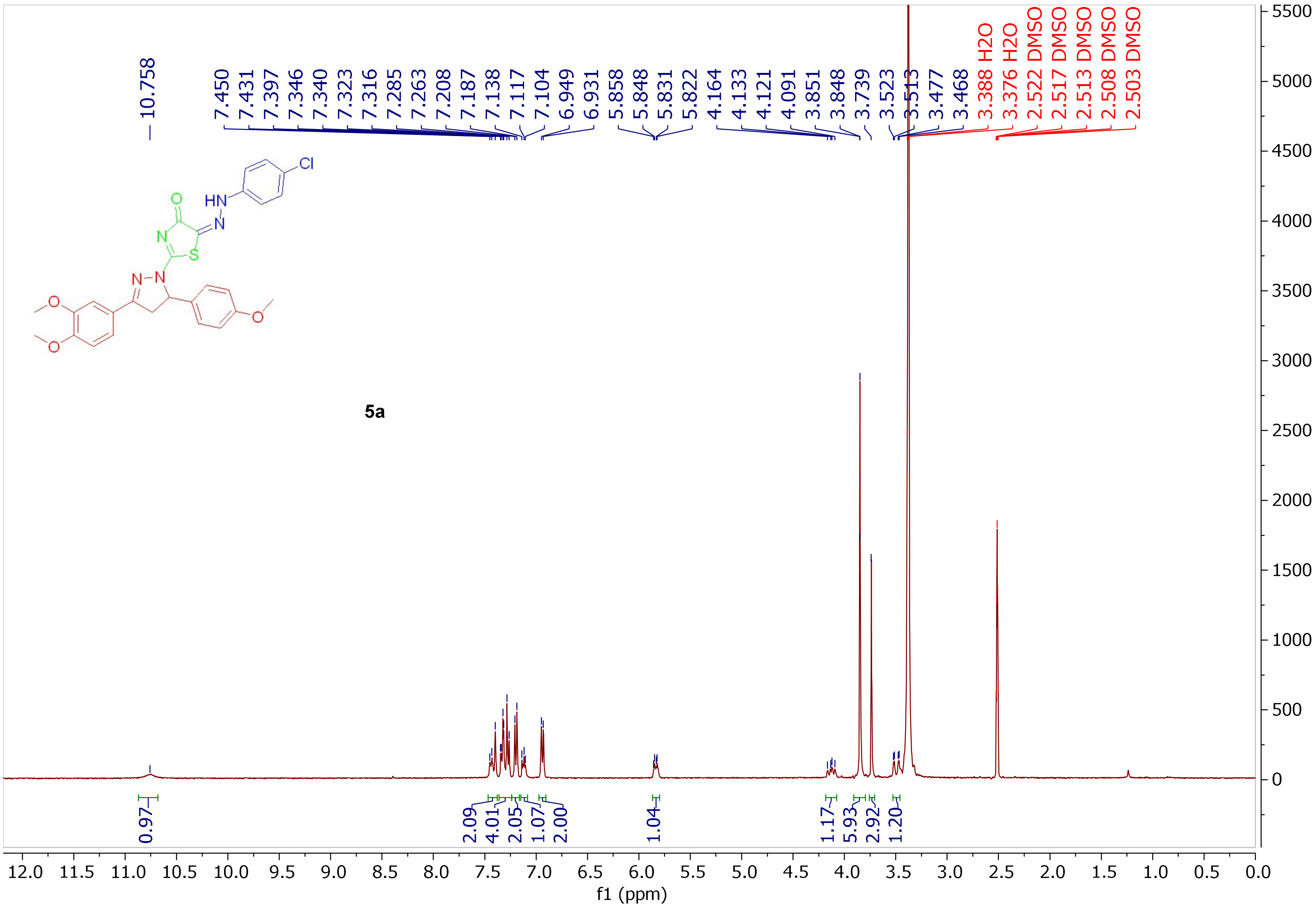

Figure S11: ^{1}H NMR of compound **5a**

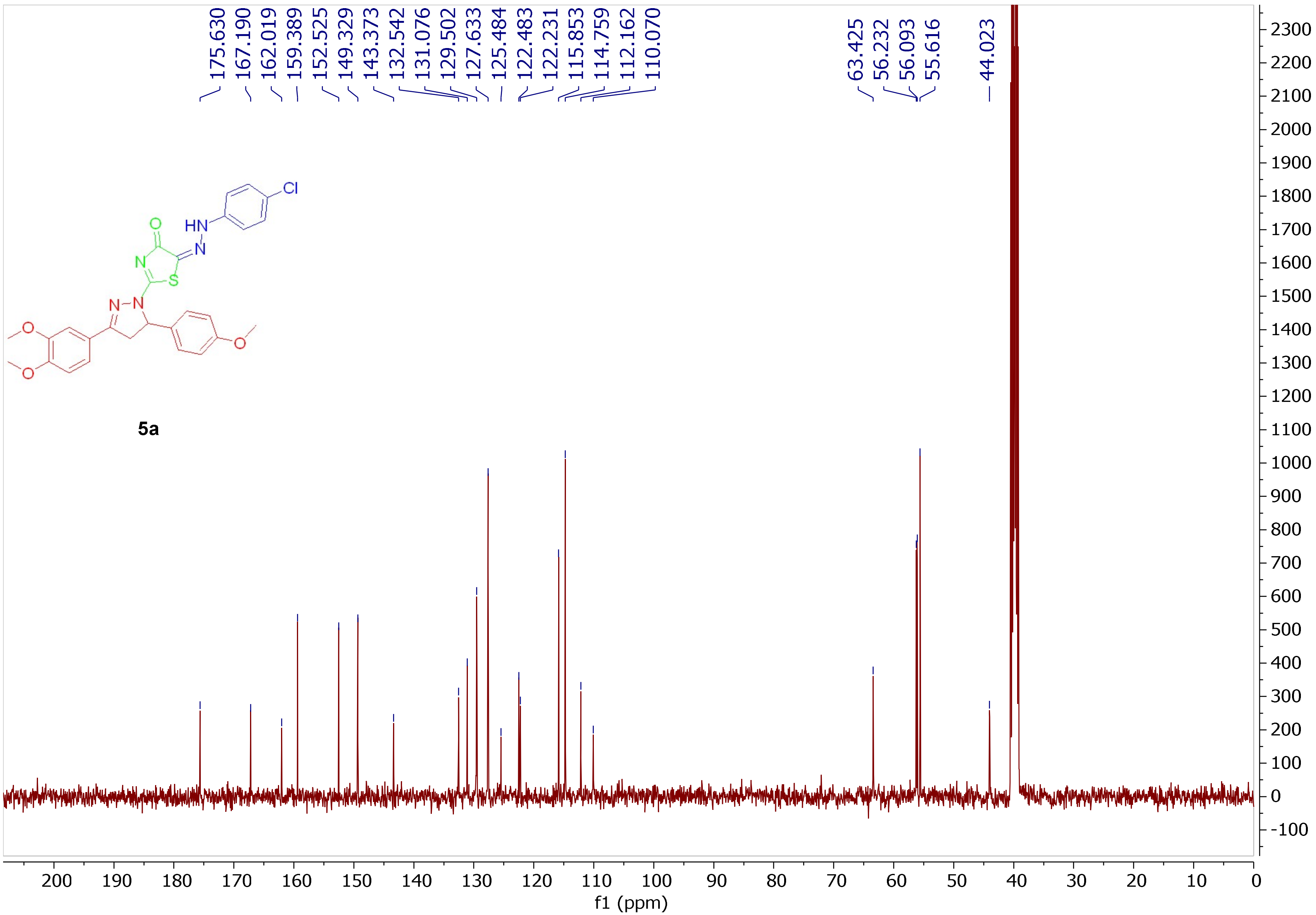

Figure S12: ^{13}C NMR of compound **5a**

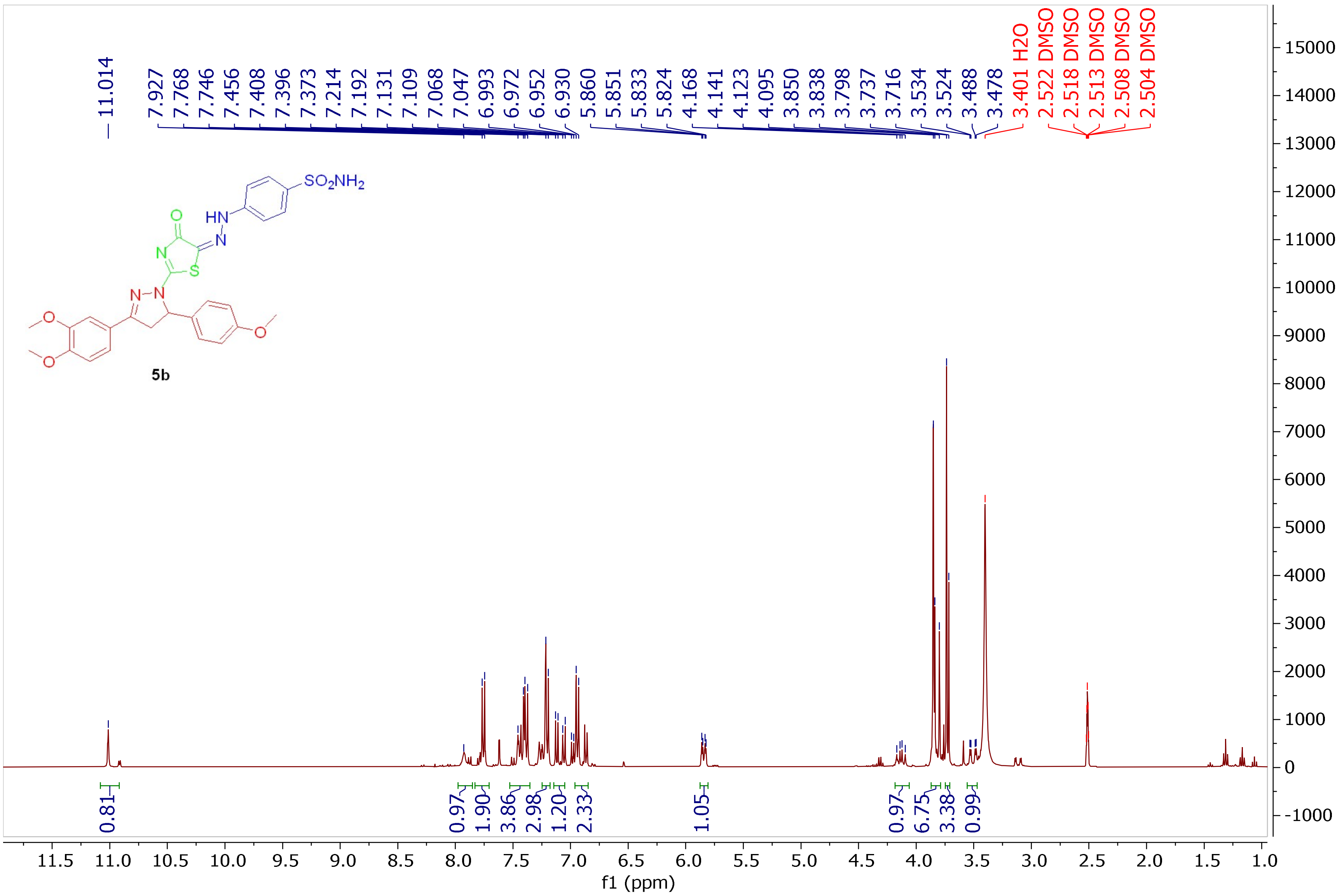

Figure S13: ^{1}H NMR of compound **5b**

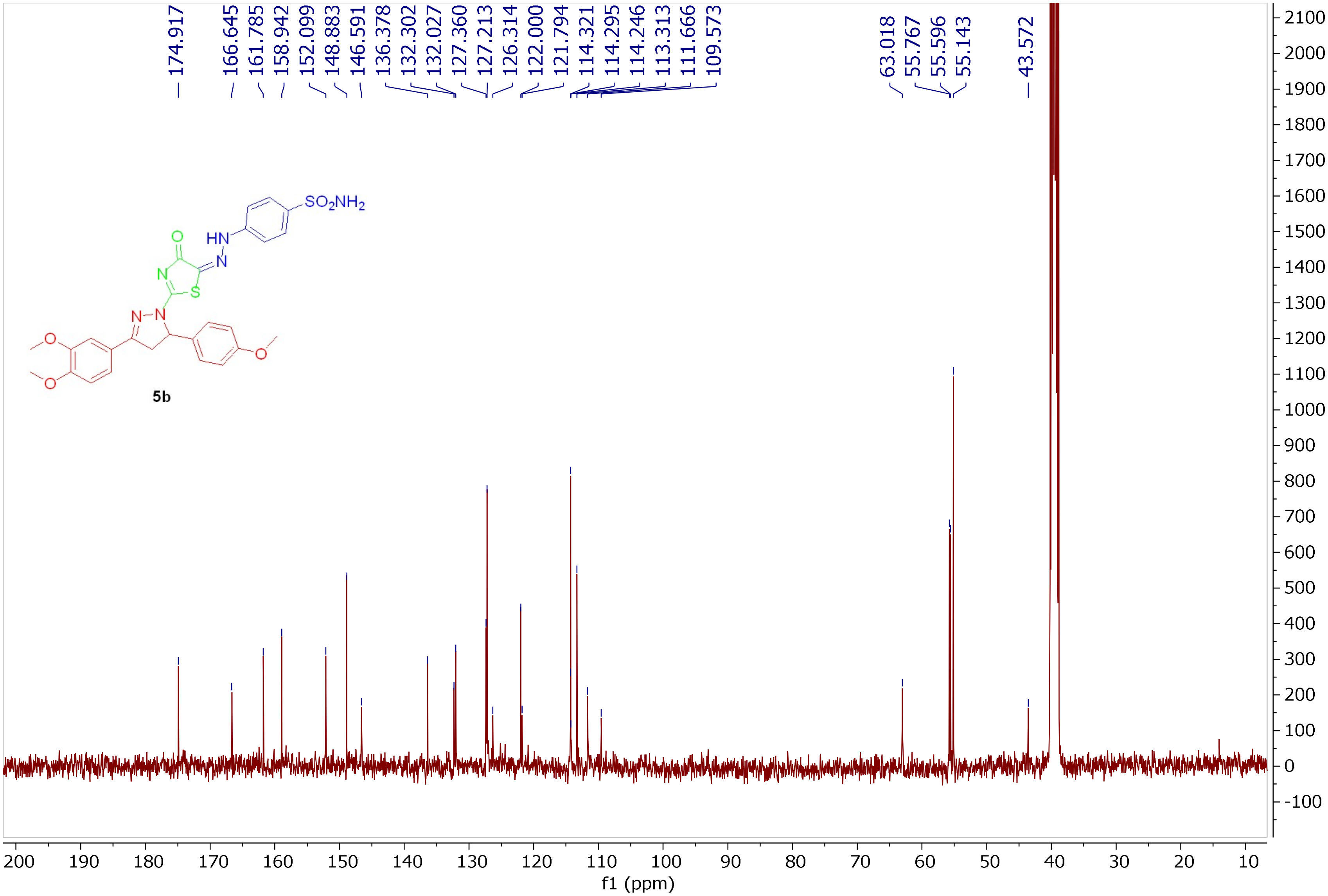

Figure S14: ^{13}C NMR of compound **5b**

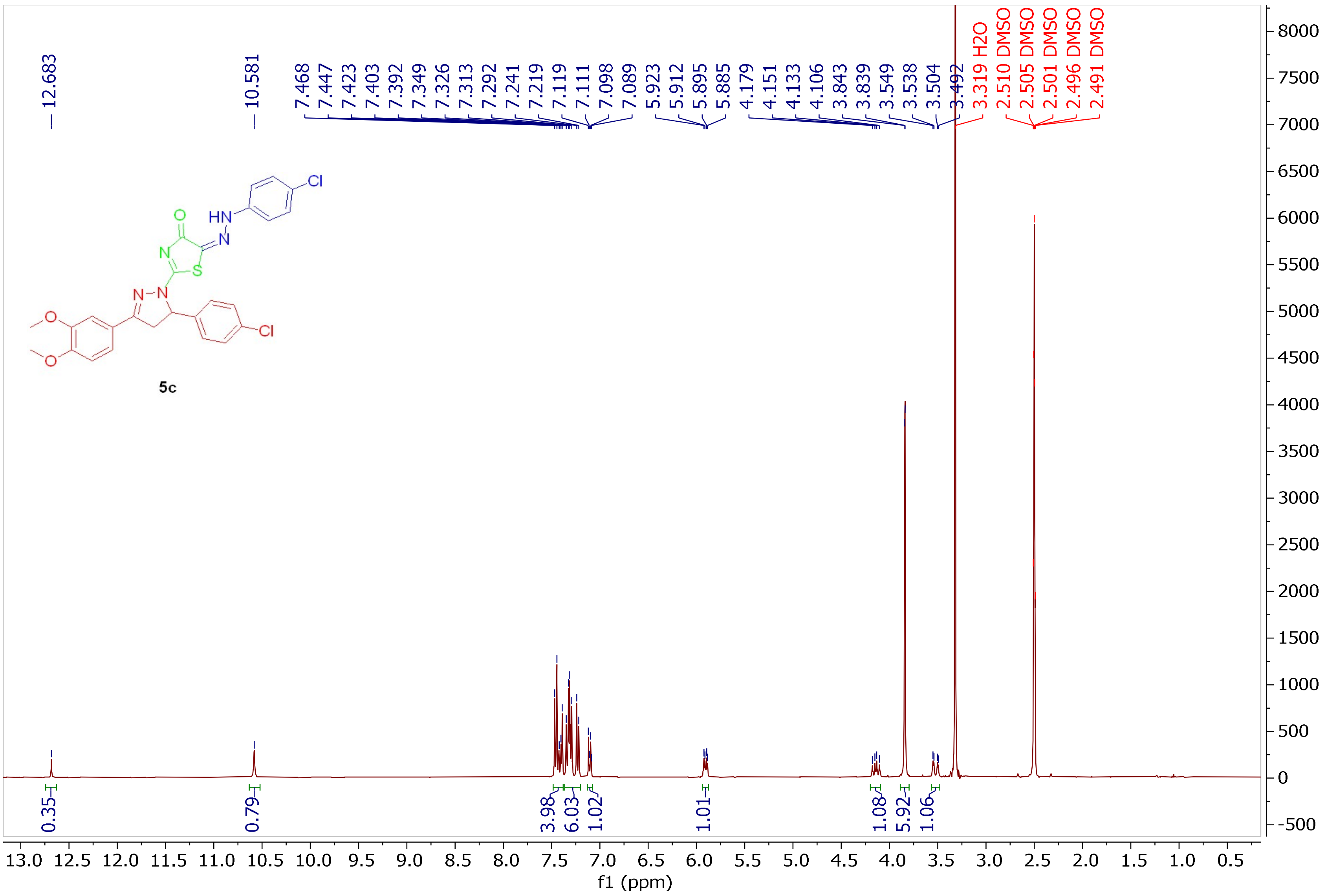

Figure S15: ^{1}H NMR of compound **5c**

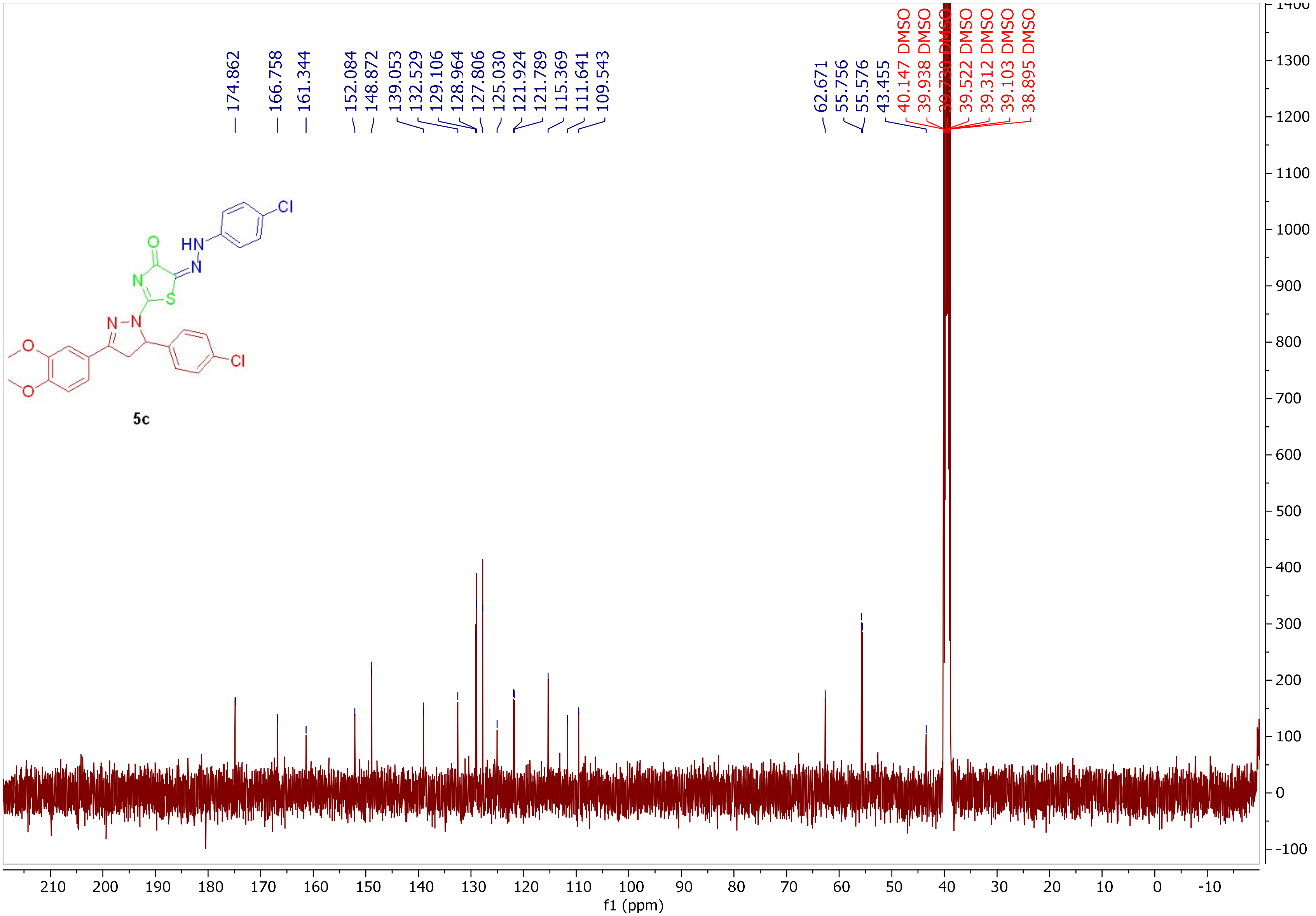

Figure S16: ^{13}C NMR of compound **5c**

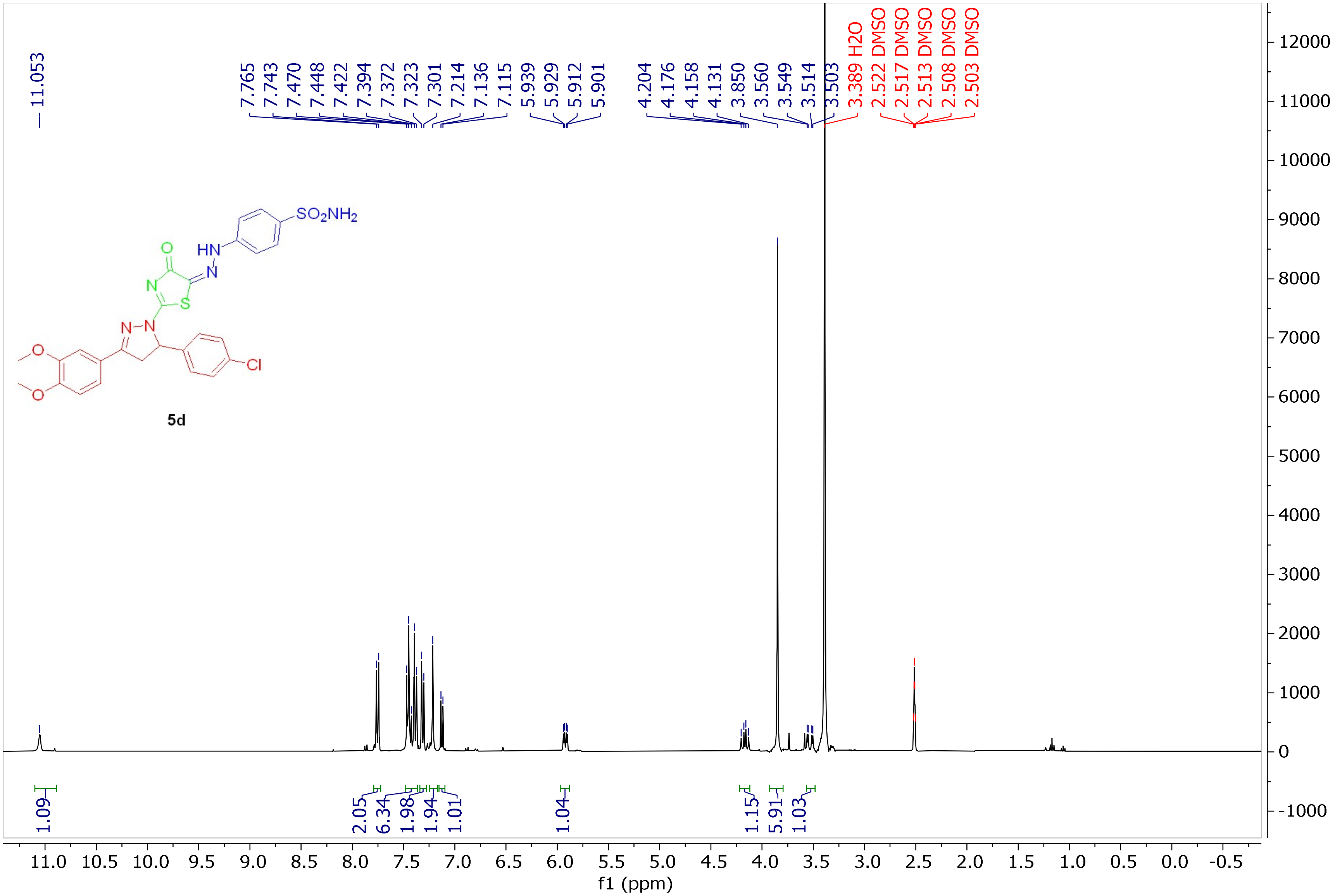

Figure S17: ^{1}H NMR of compound **5d**

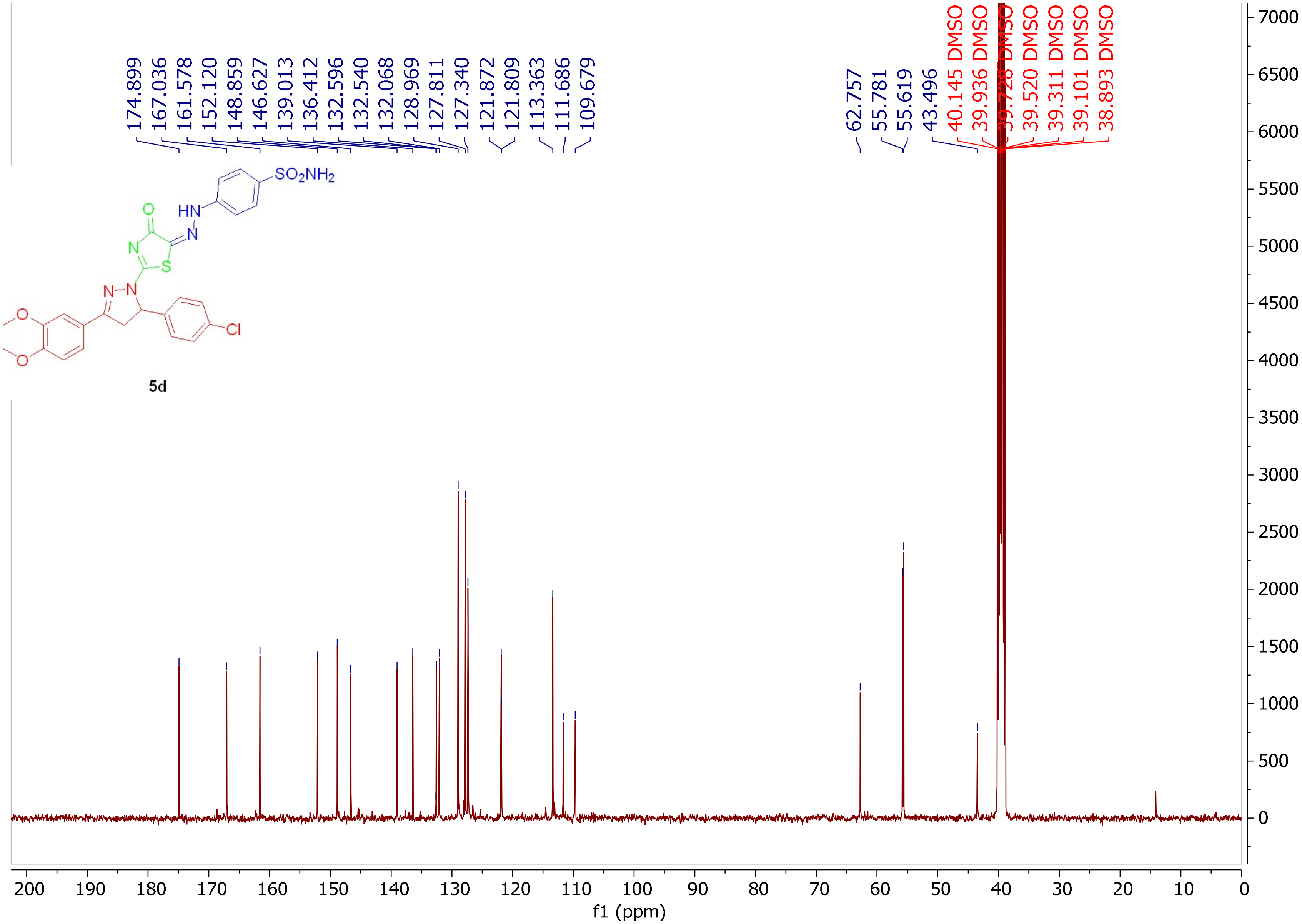

Figure S18: ^{13}C NMR of compound **5d**

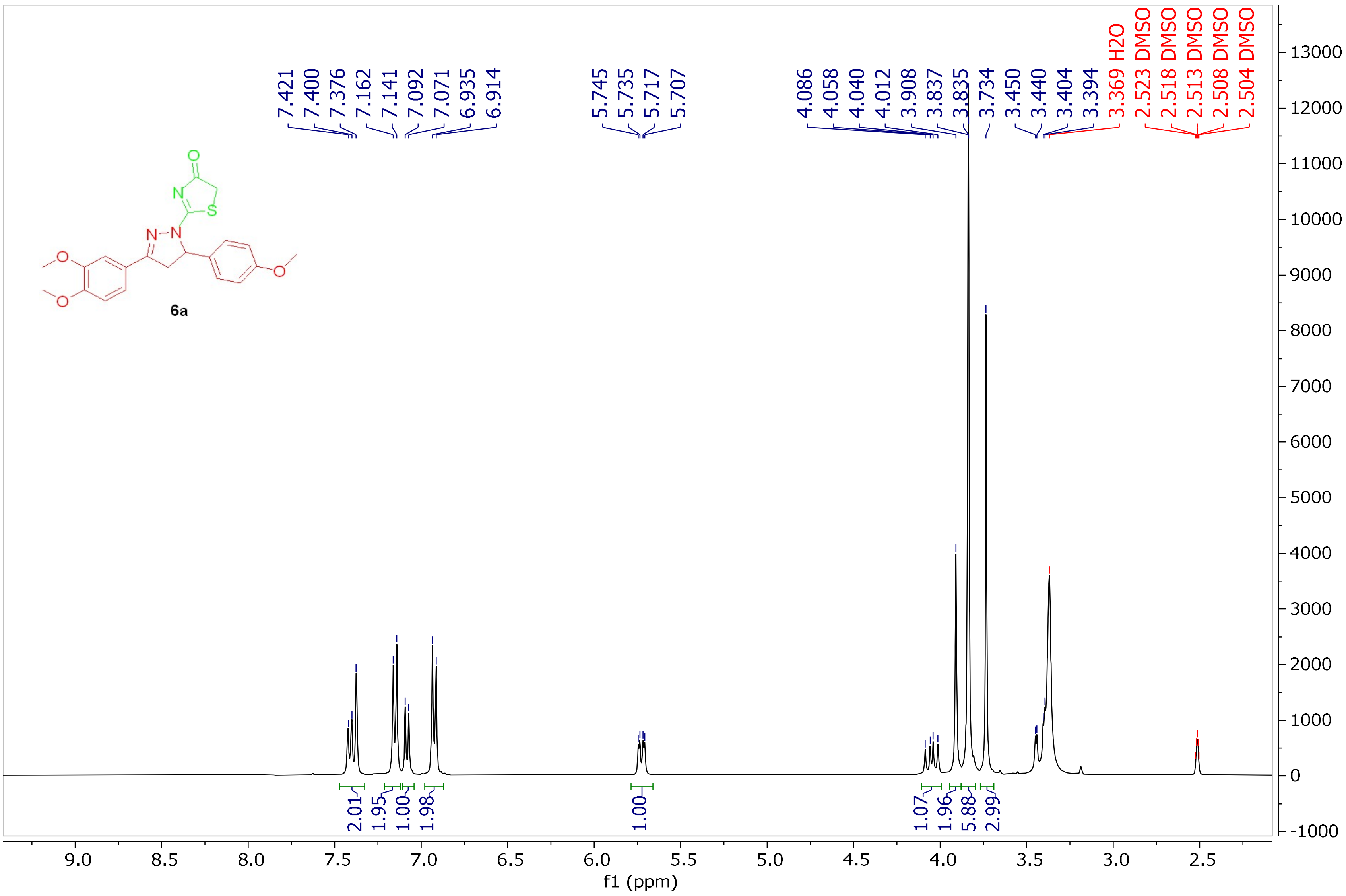

Figure S19: ^{1}H NMR of compound **6a**

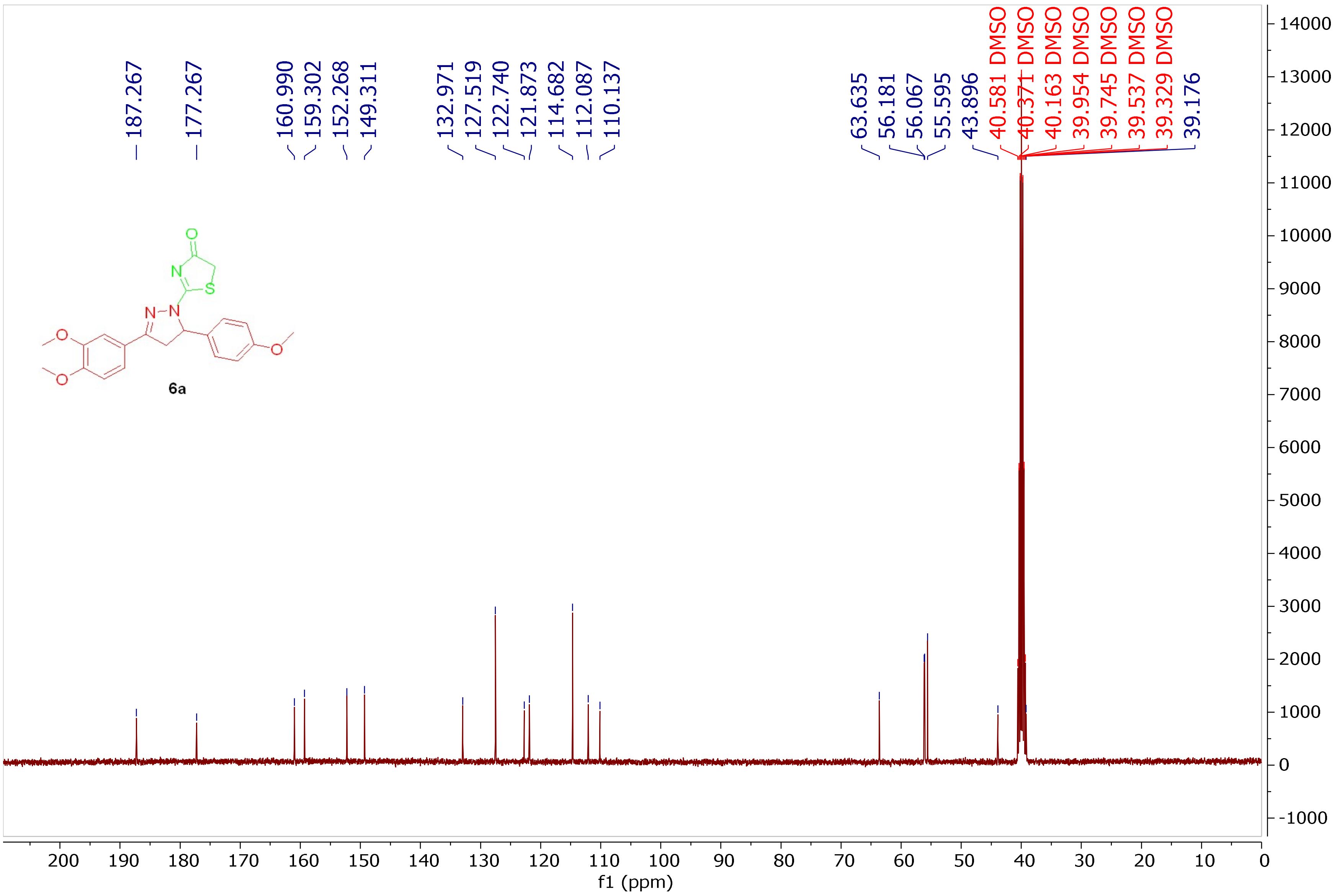

Figure S20: ^{13}C NMR of compound **6a**

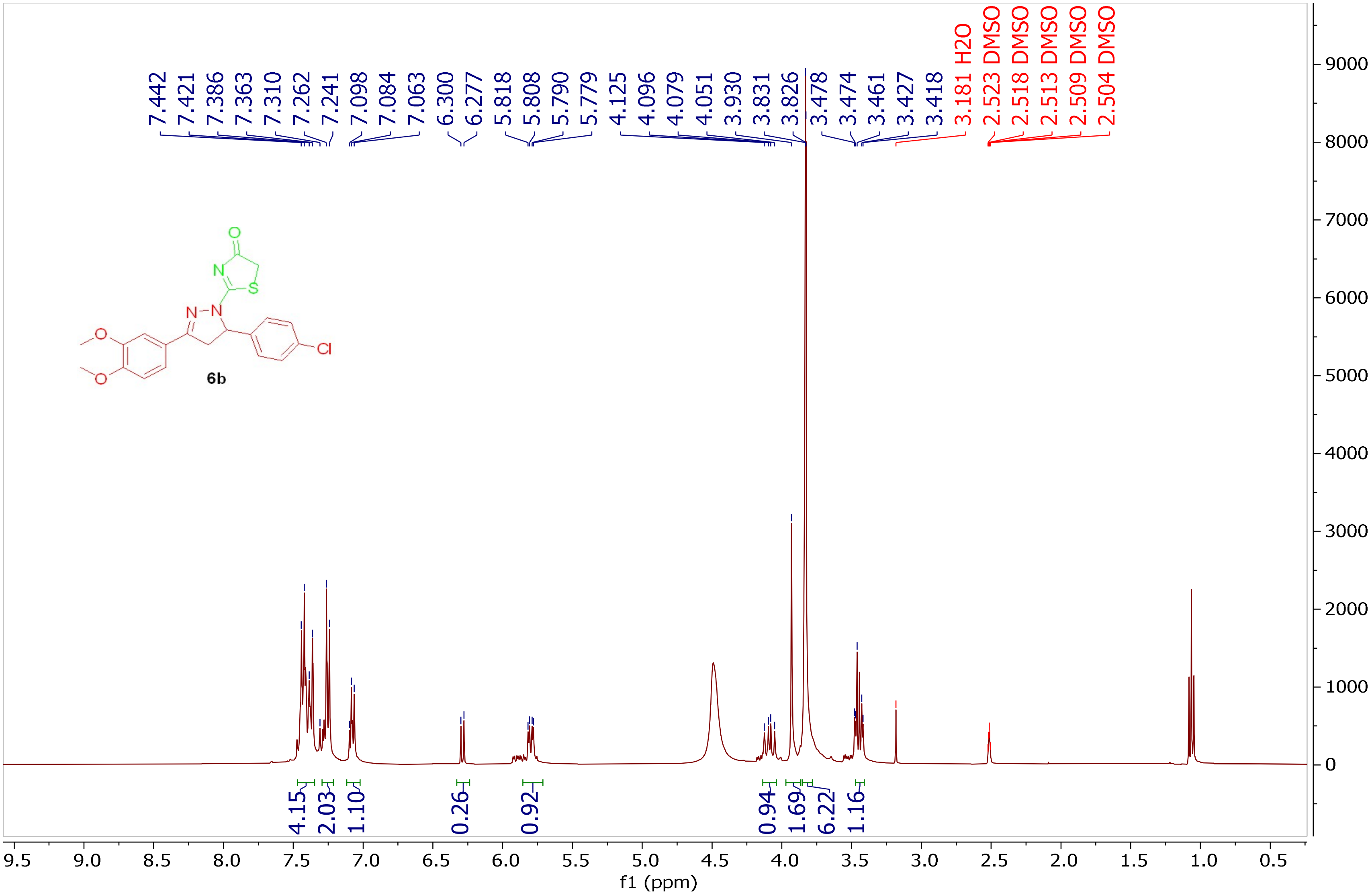

Figure S21: ^{1}H NMR of compound **6b**

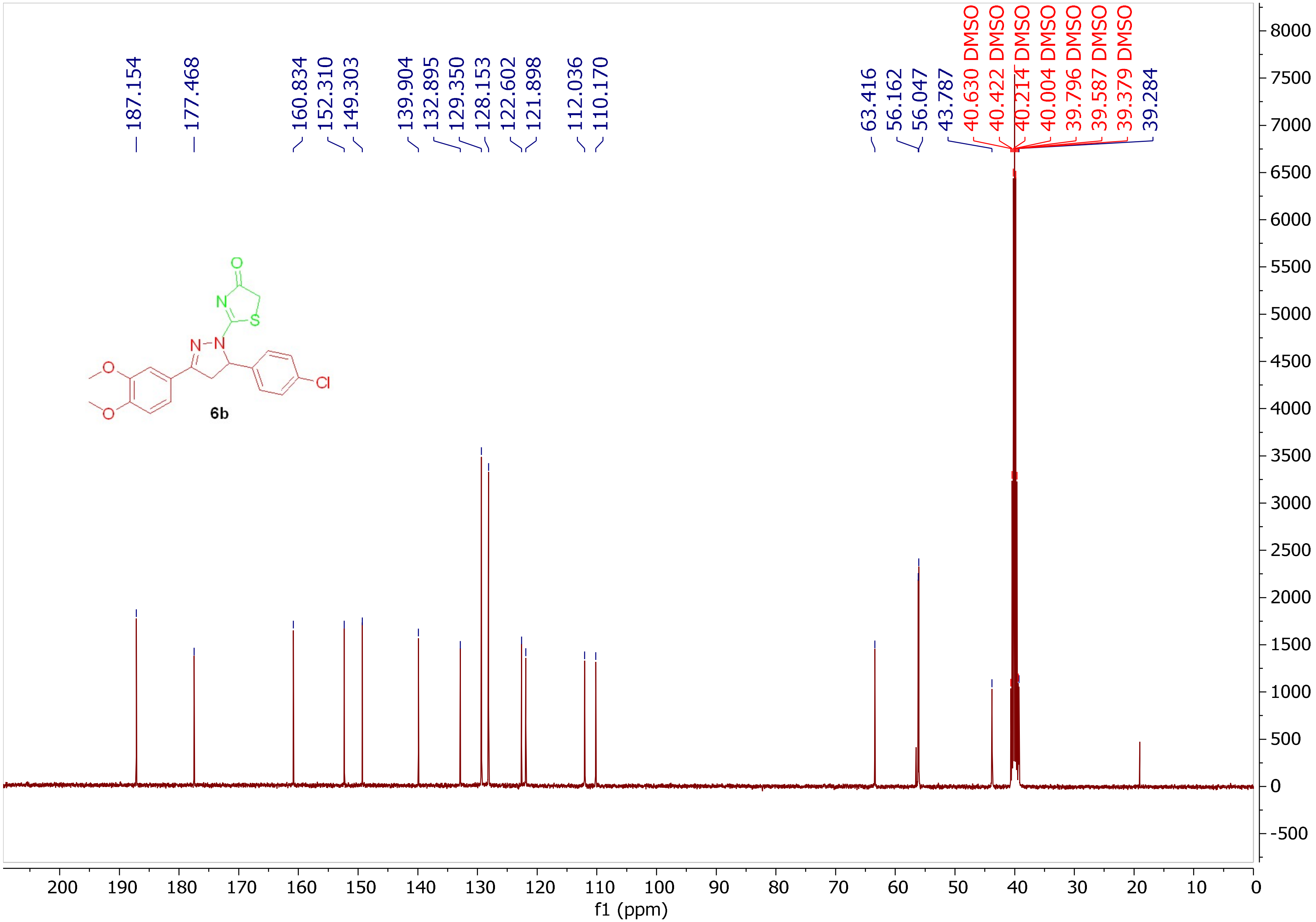

Figure S22: ^{13}C NMR of compound **6b**

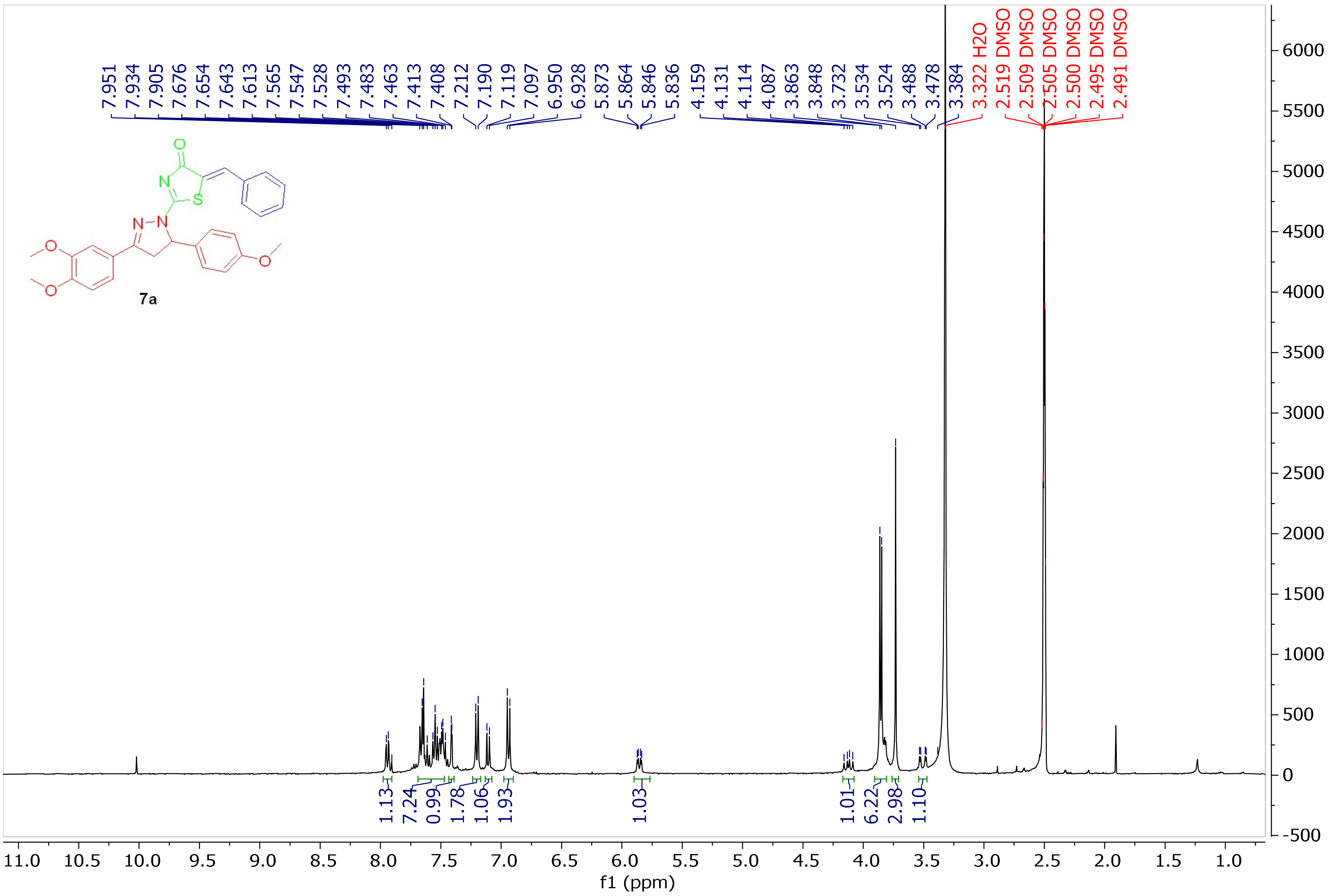

Figure S23: ^{1}H NMR of compound **7a**

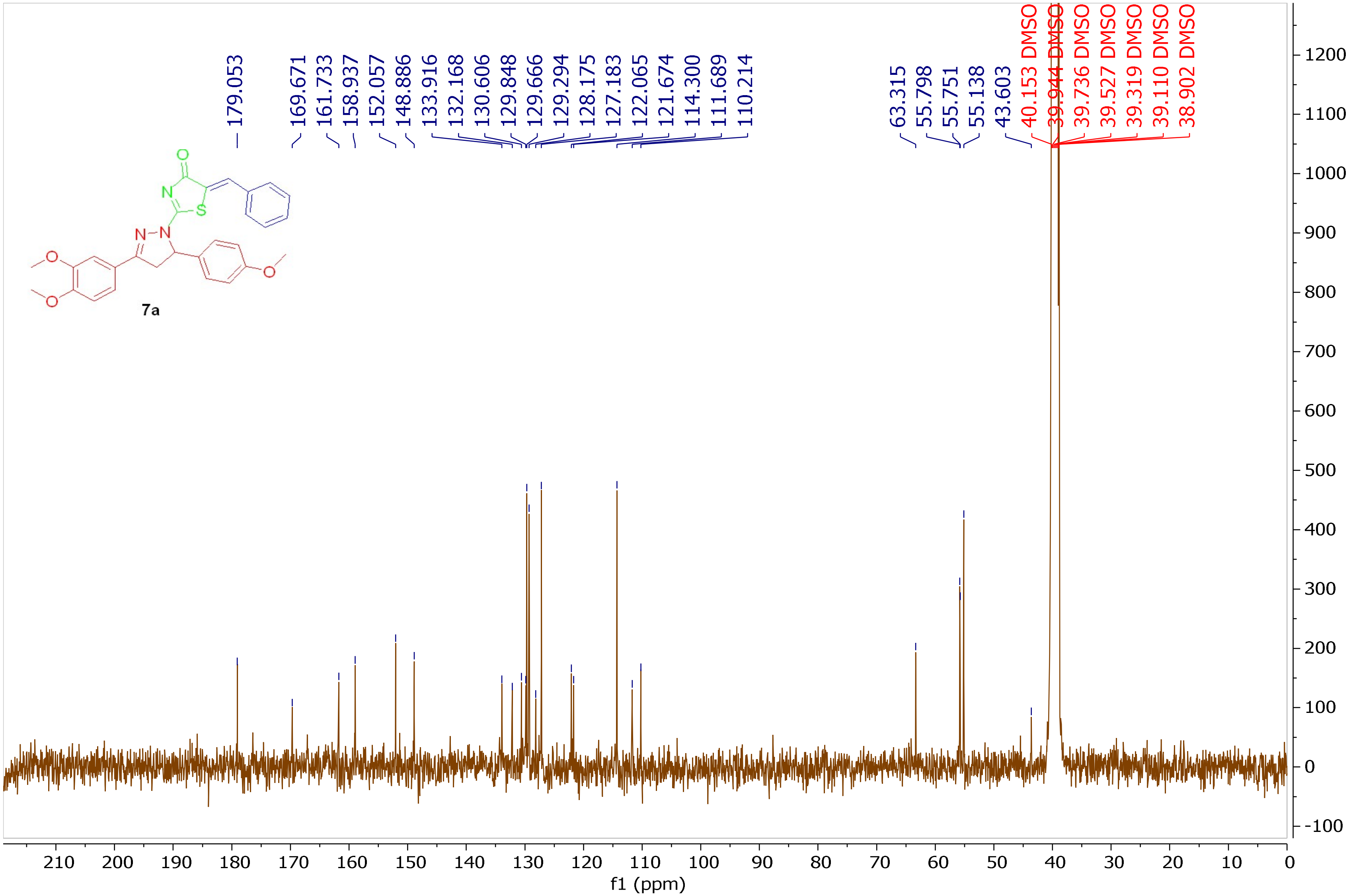

Figure S24: ^{13}C NMR of compound **7a**

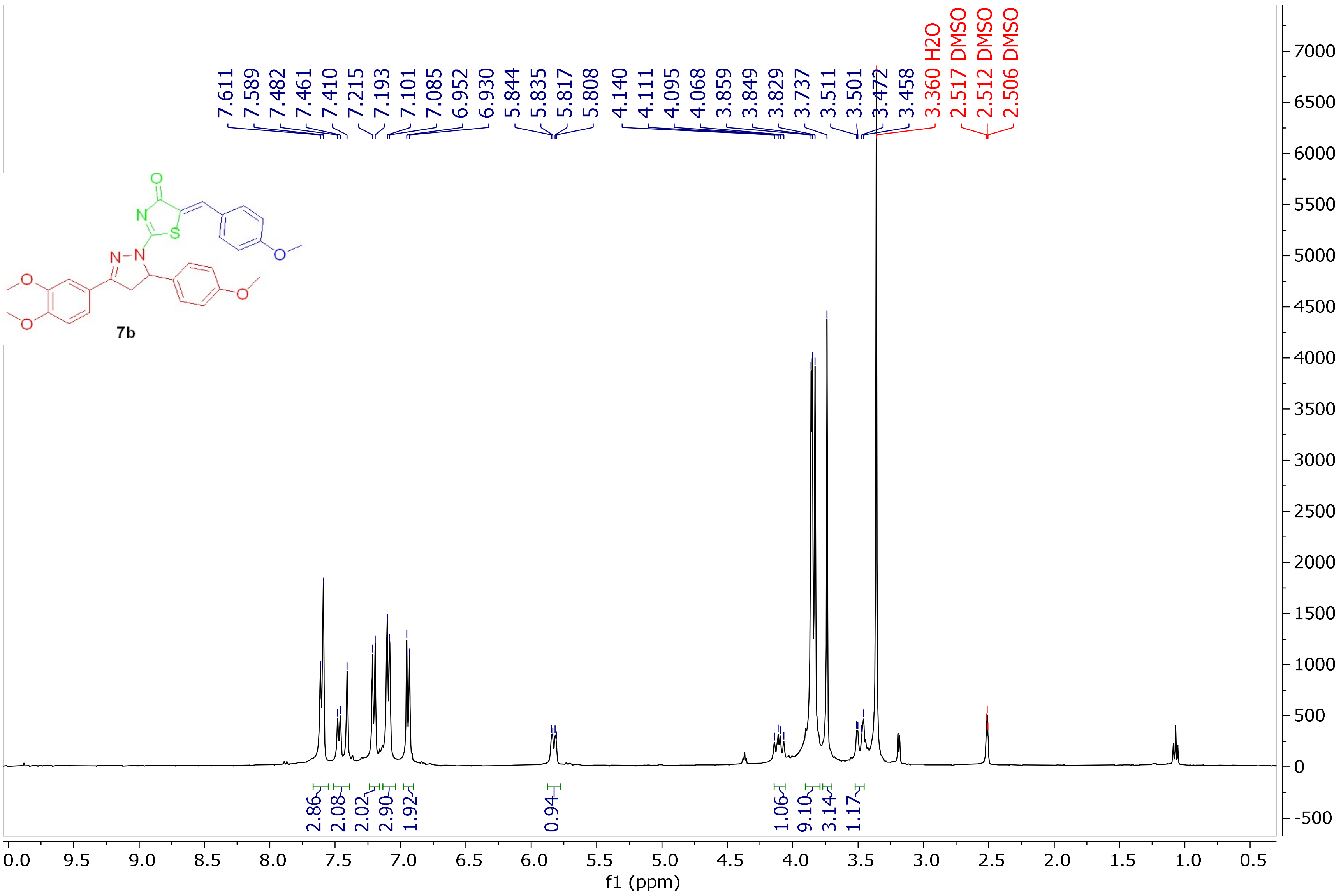

Figure S25: ^{1}H NMR of compound **7b**

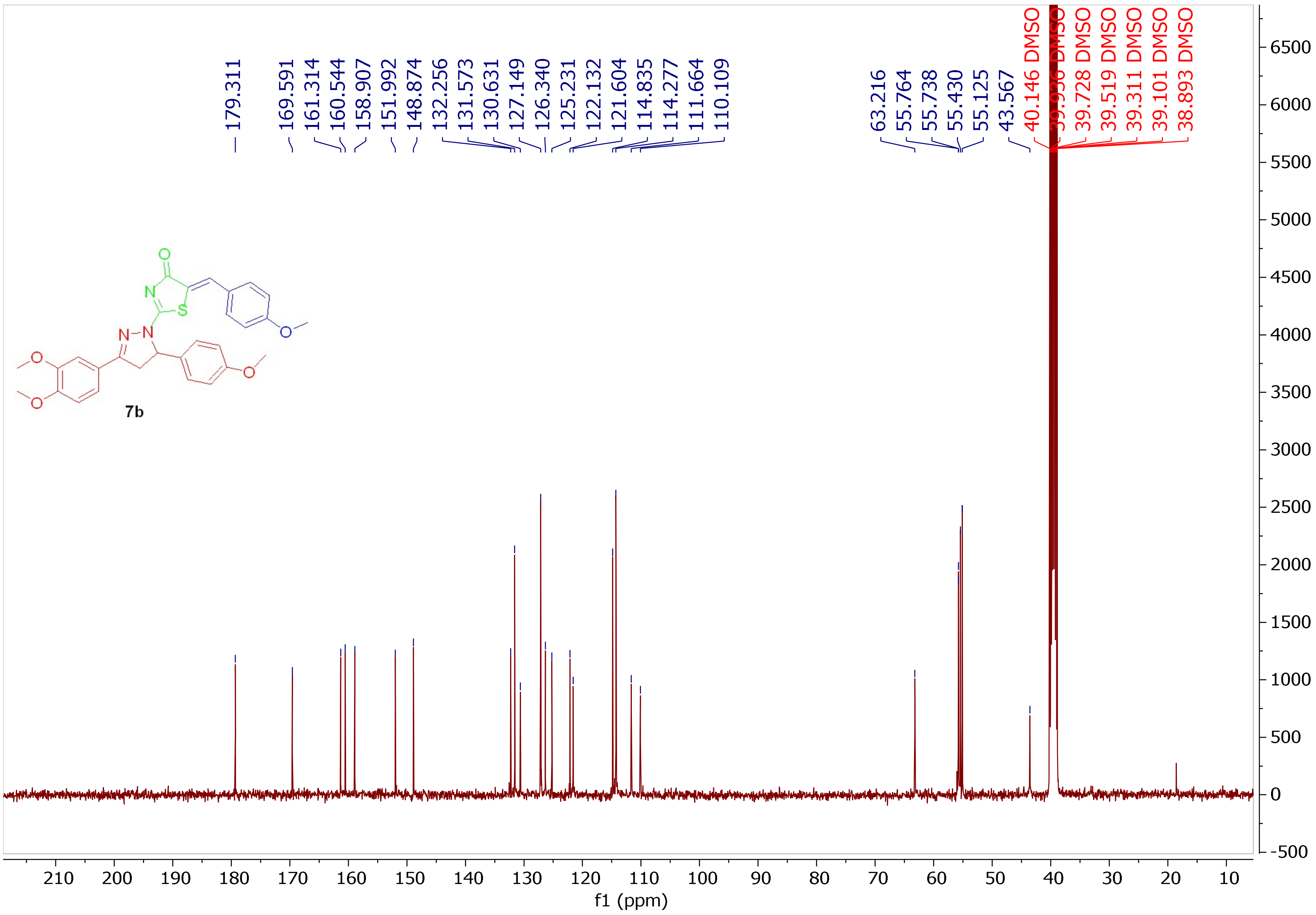

Figure S26: ^{13}C NMR of compound **7b**

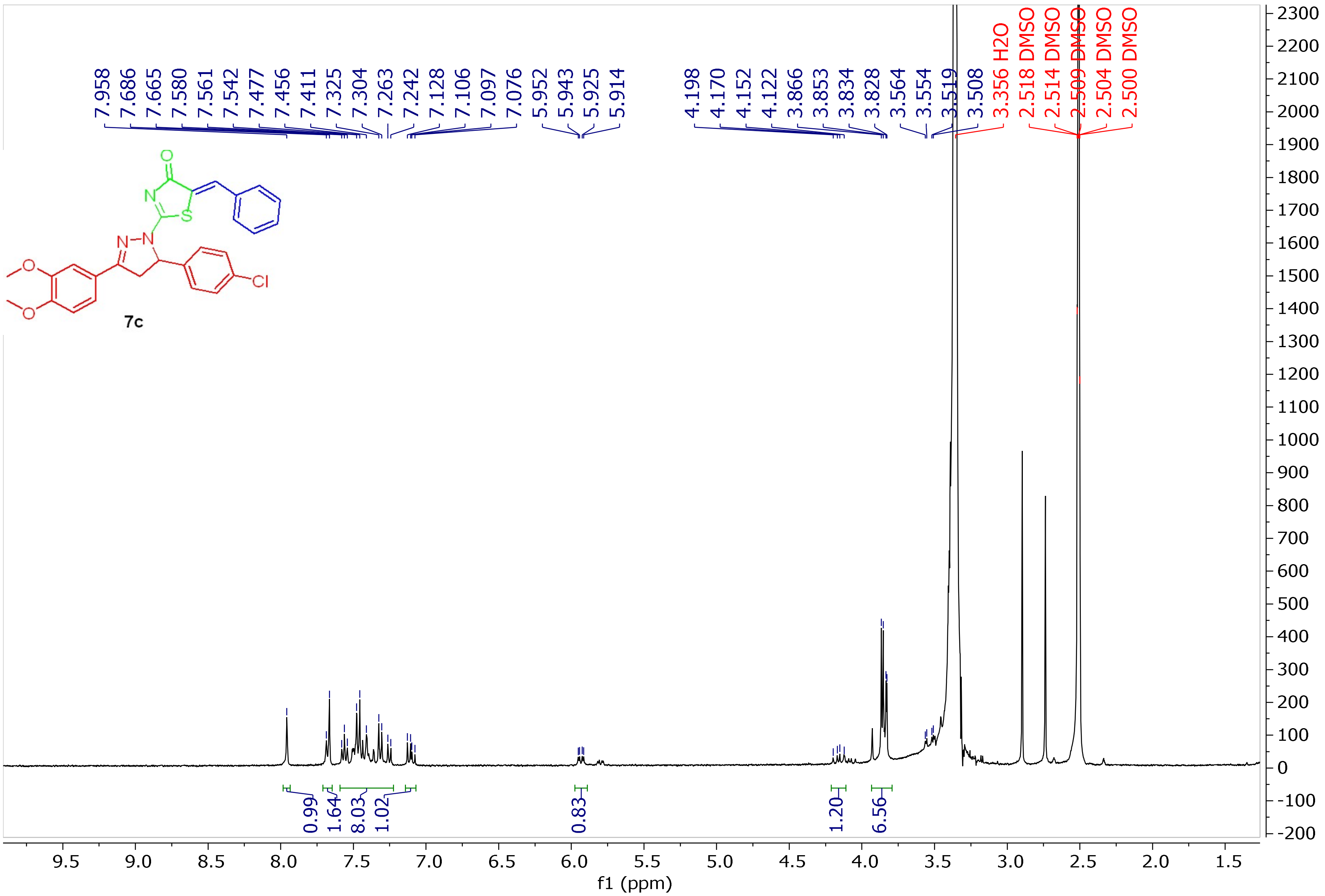

Figure S27: ^{1}H NMR of compound **7c**

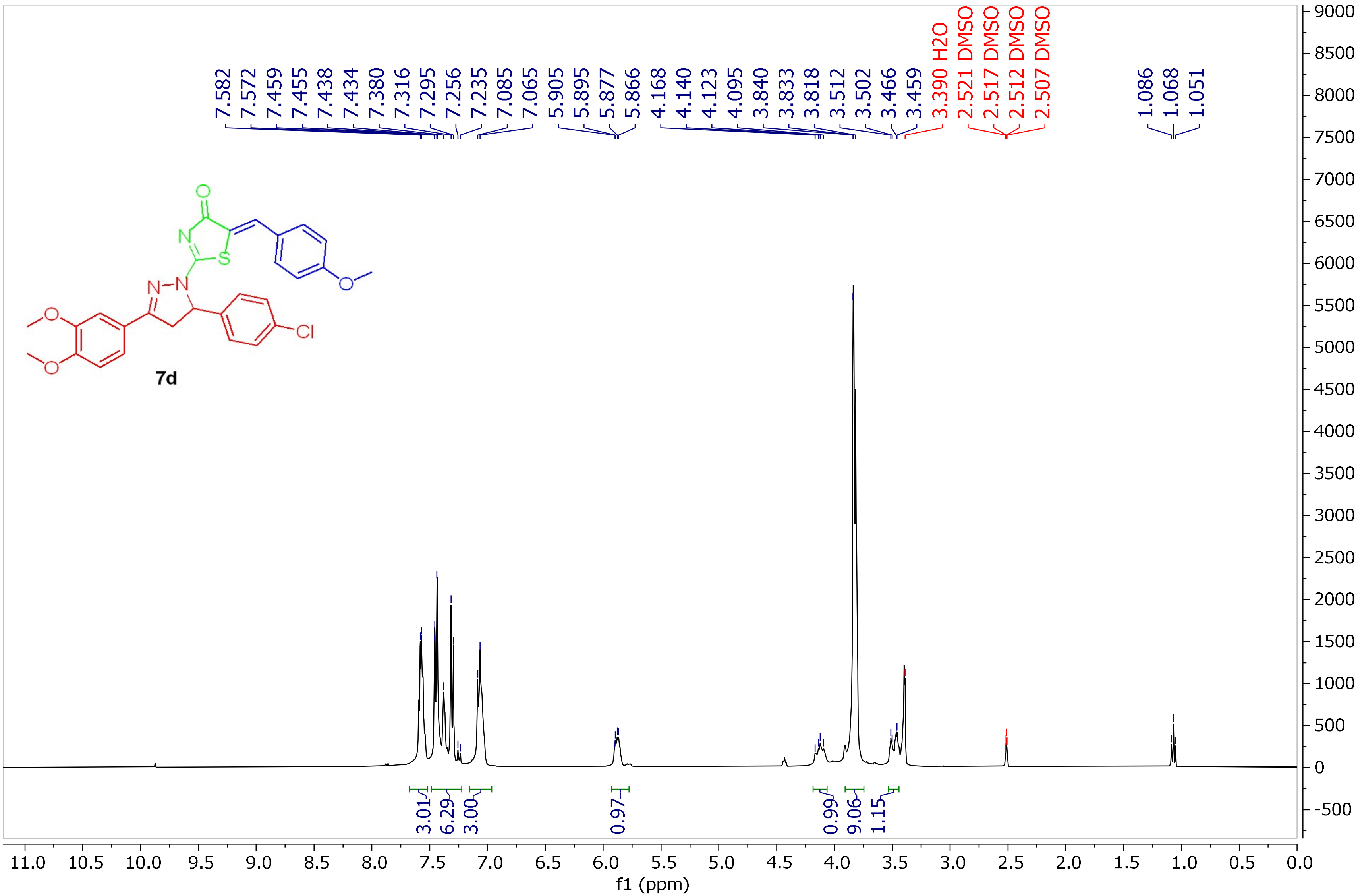

Figure S28: ^{1}H NMR of compound **7d**

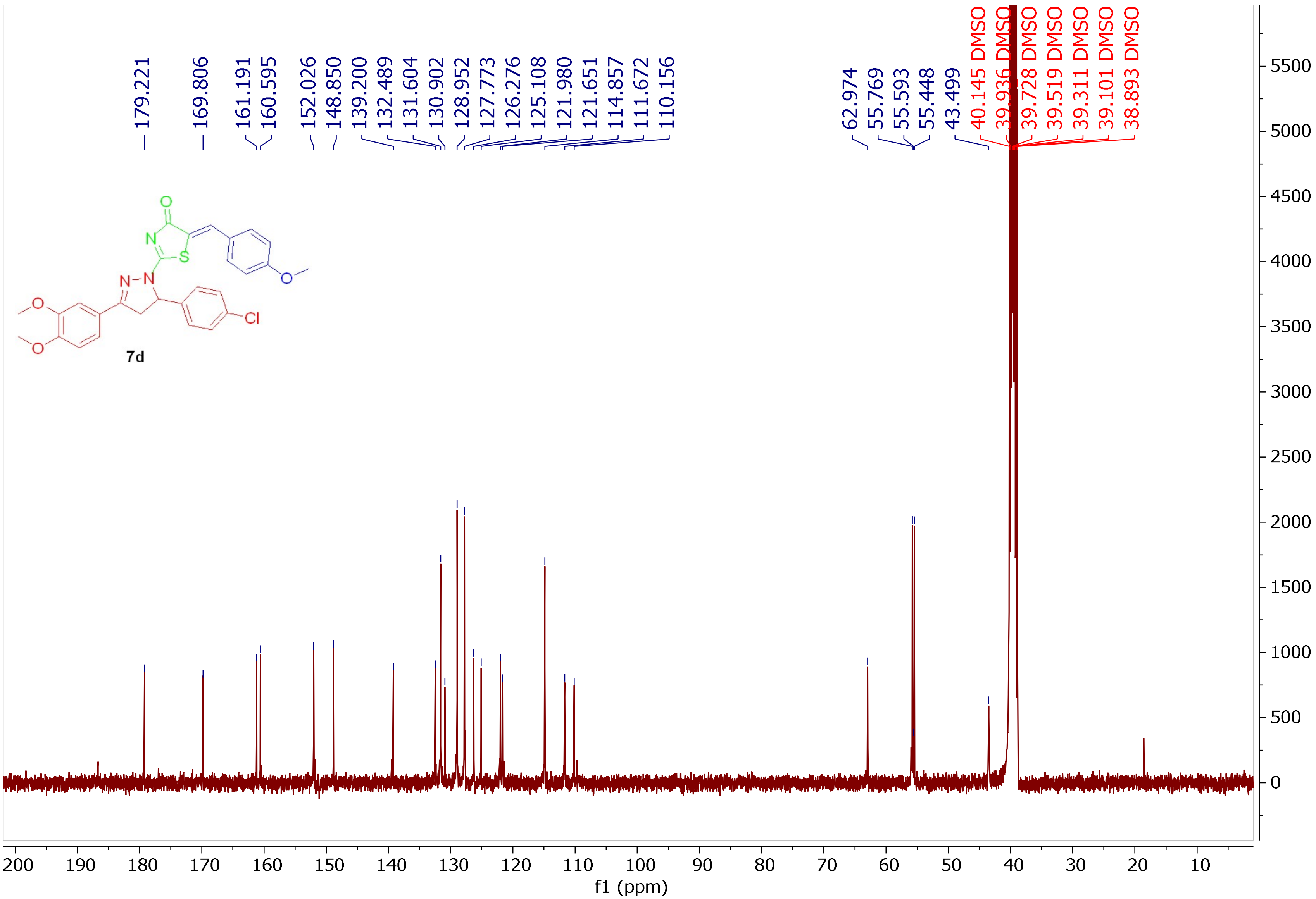

Figure S29: ^{13}C NMR of compound **7d**

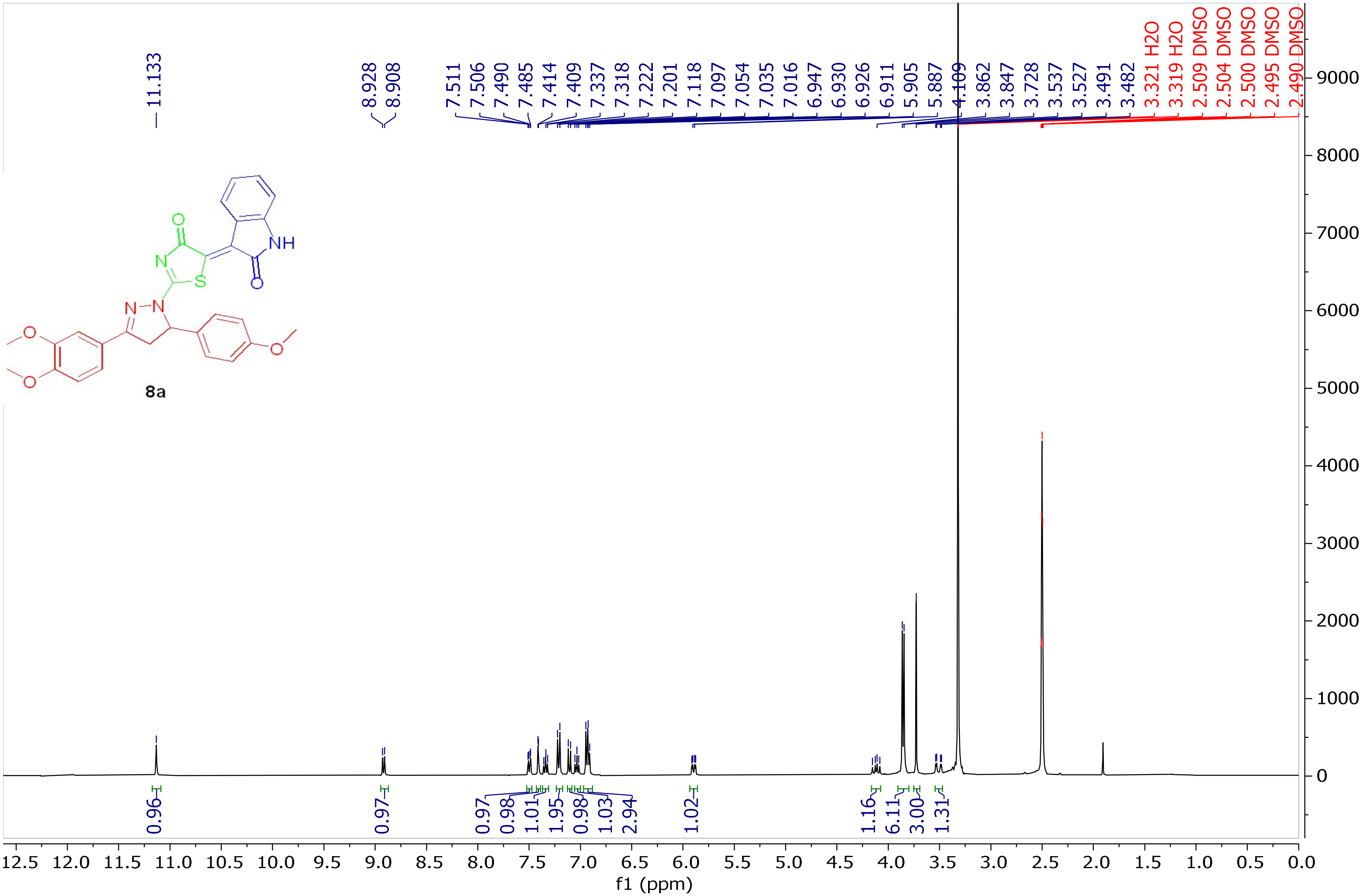

Figure S30: ^{1}H NMR of compound **8a**

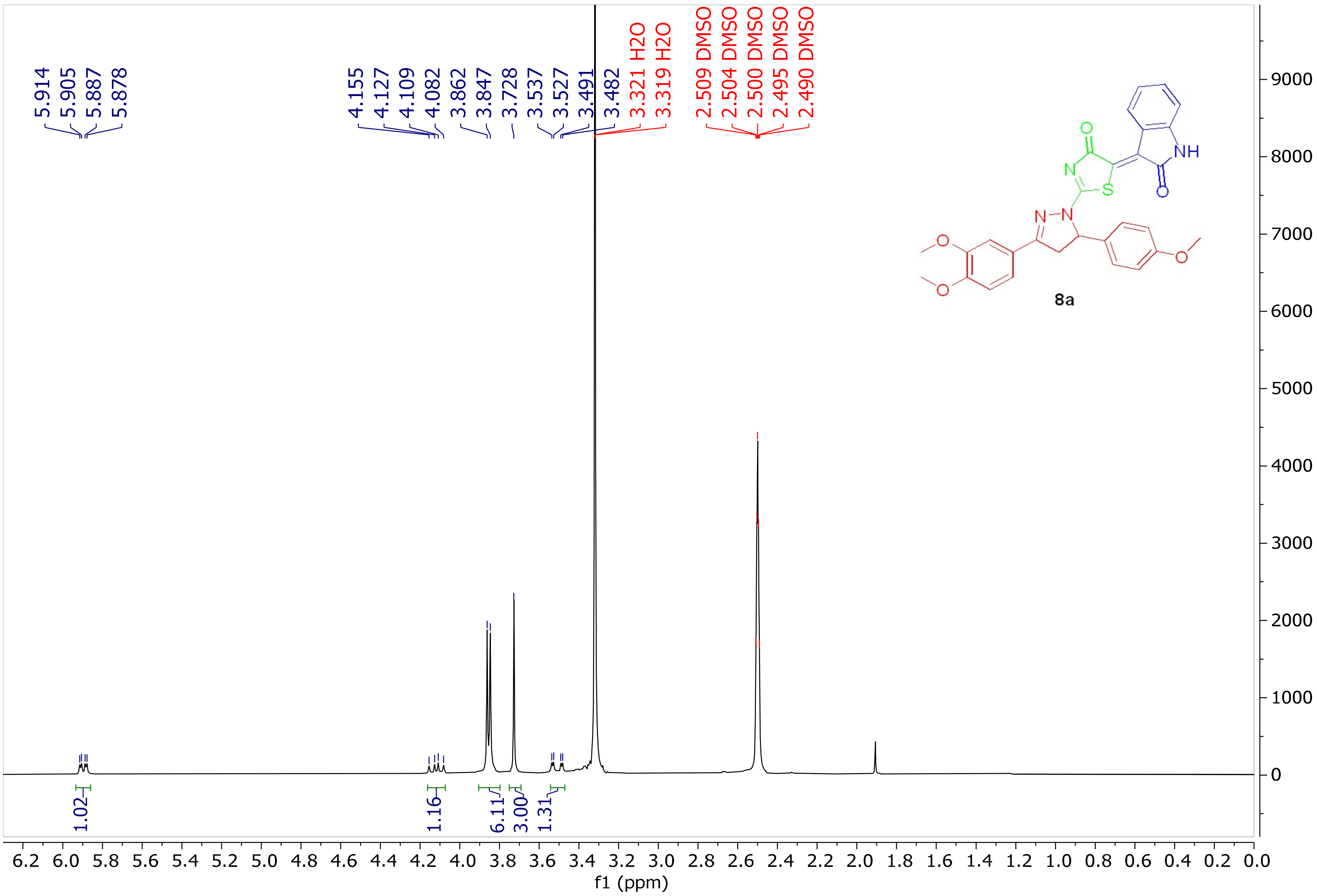

Figure S31: ^{1}H NMR of compound **8a**, Aliphatic region

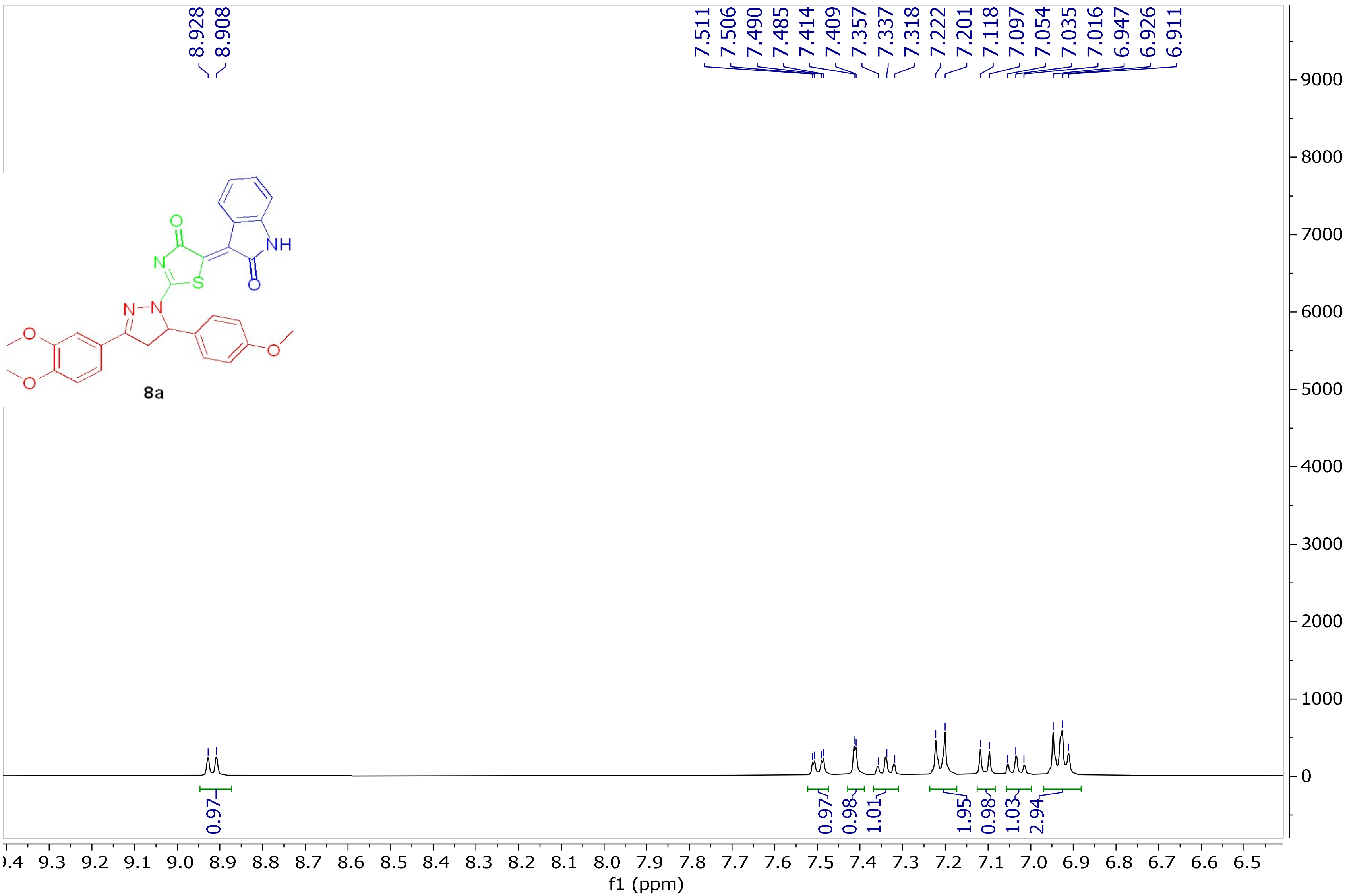

Figure S32: ^{1}H NMR of compound **8a**, aromatic region

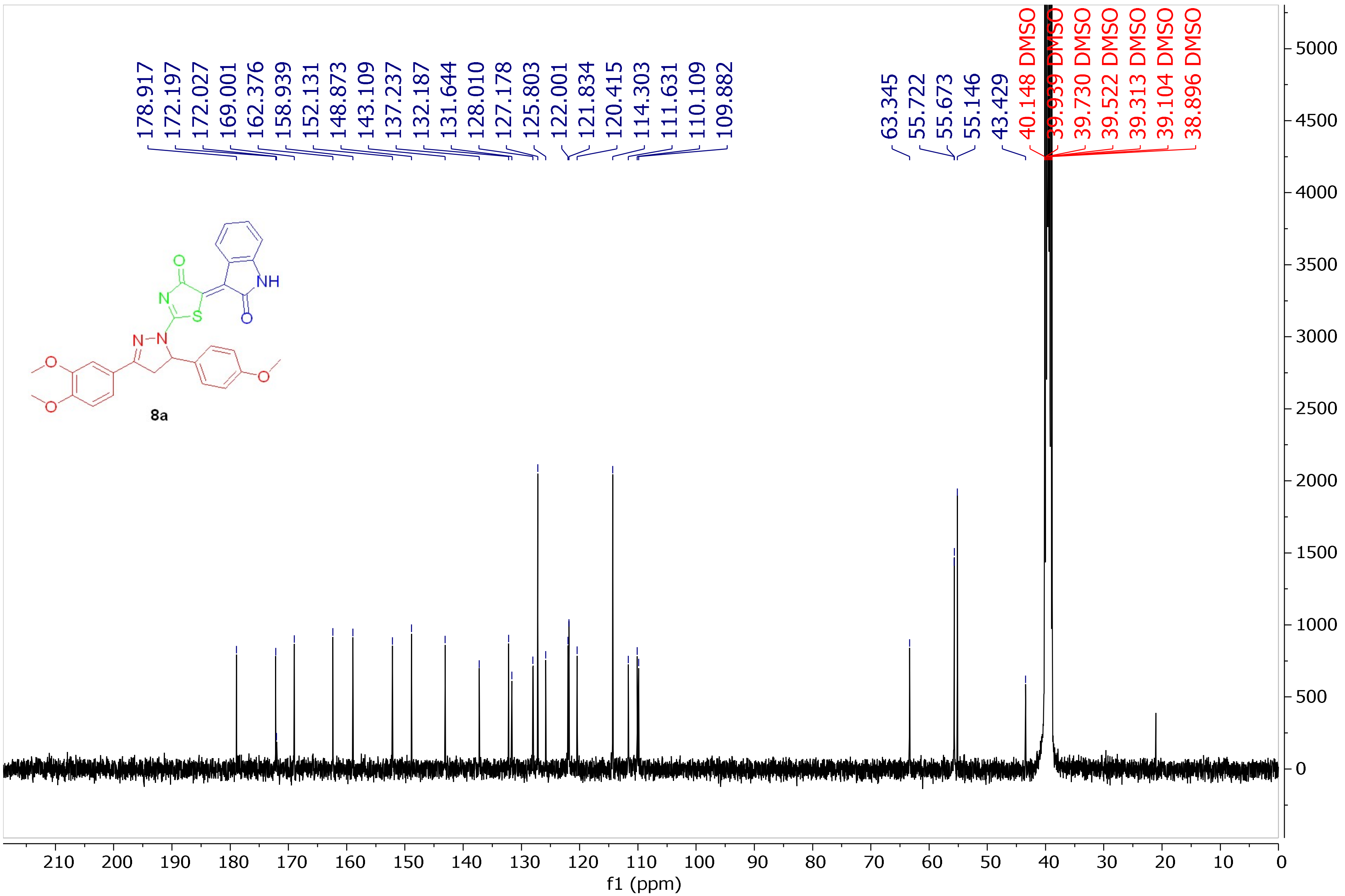

Figure S33: ^{13}C NMR of compound **8a**

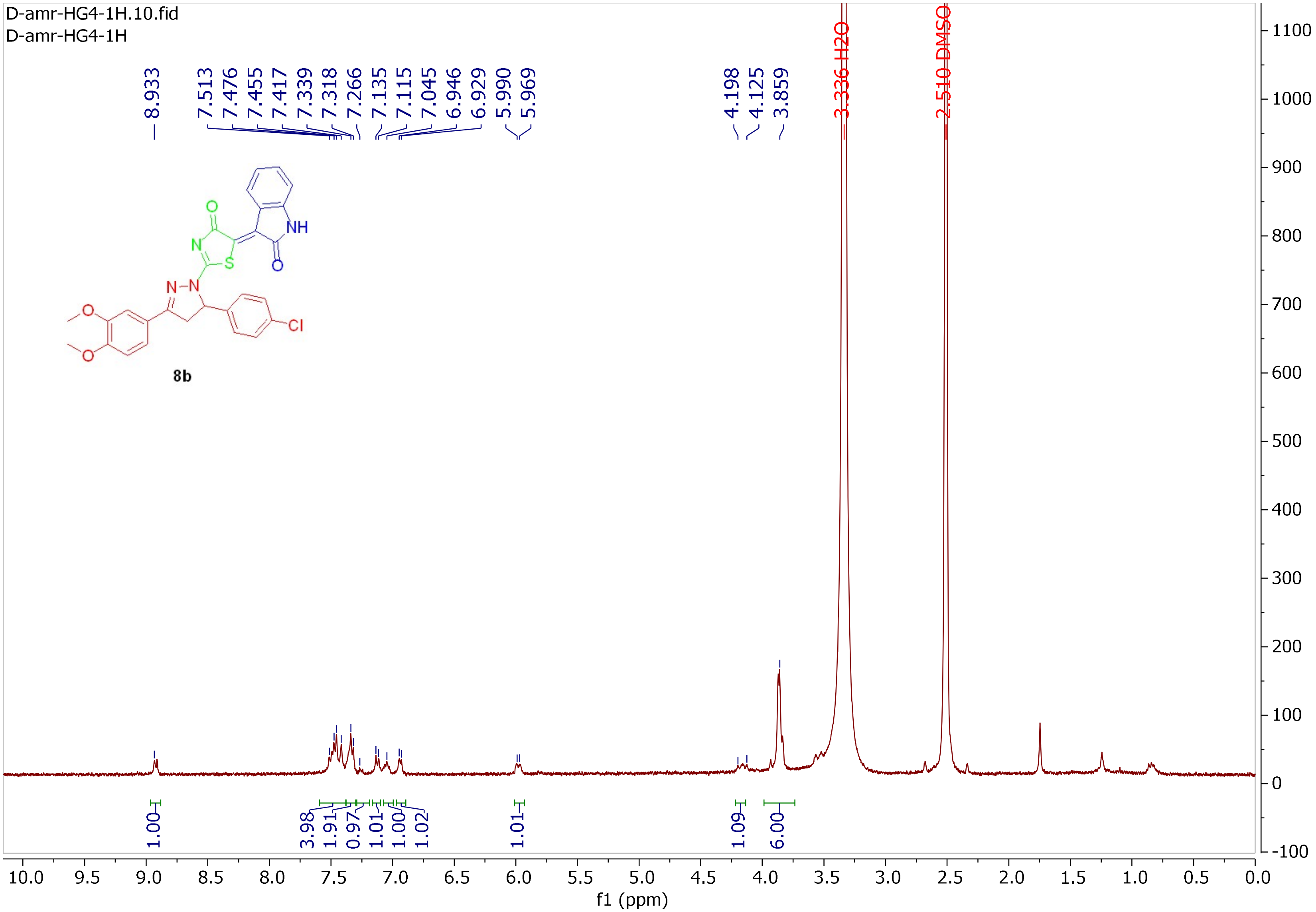

Figure S34: ^{1}H NMR of compound **8b**

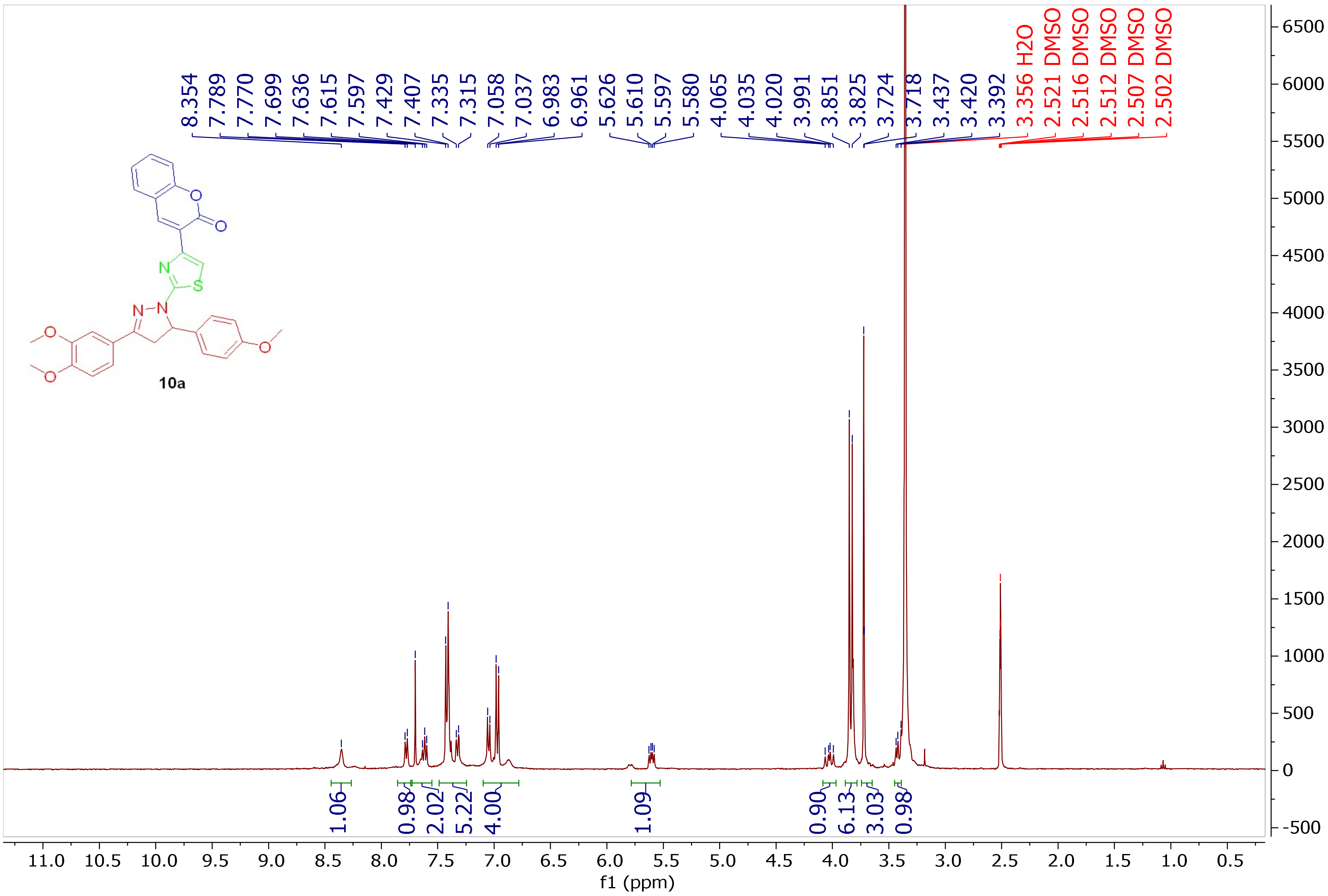

Figure S35: ^{1}H NMR of compound **10a**

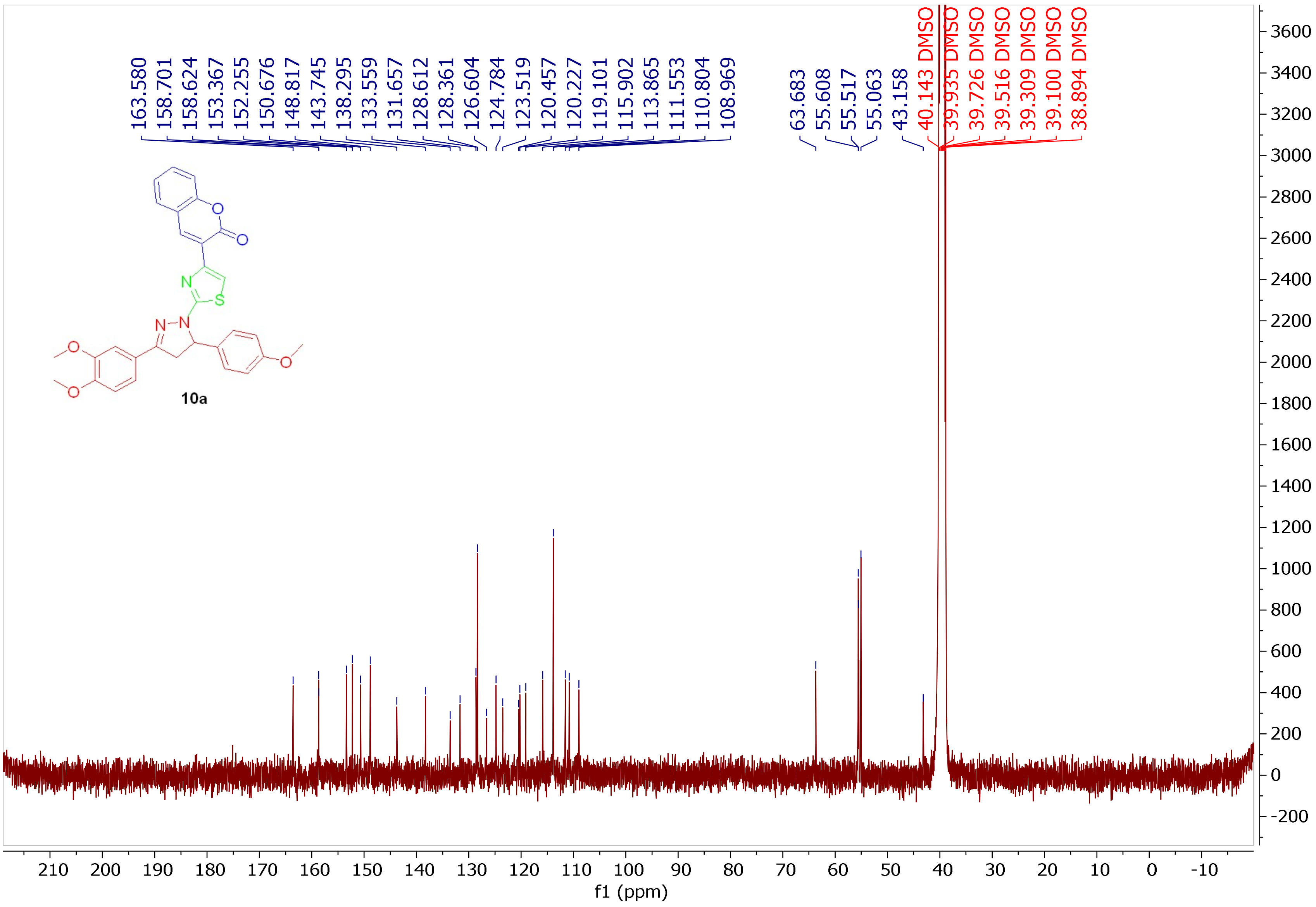

Figure S36: ^{13}C NMR of compound **10a**

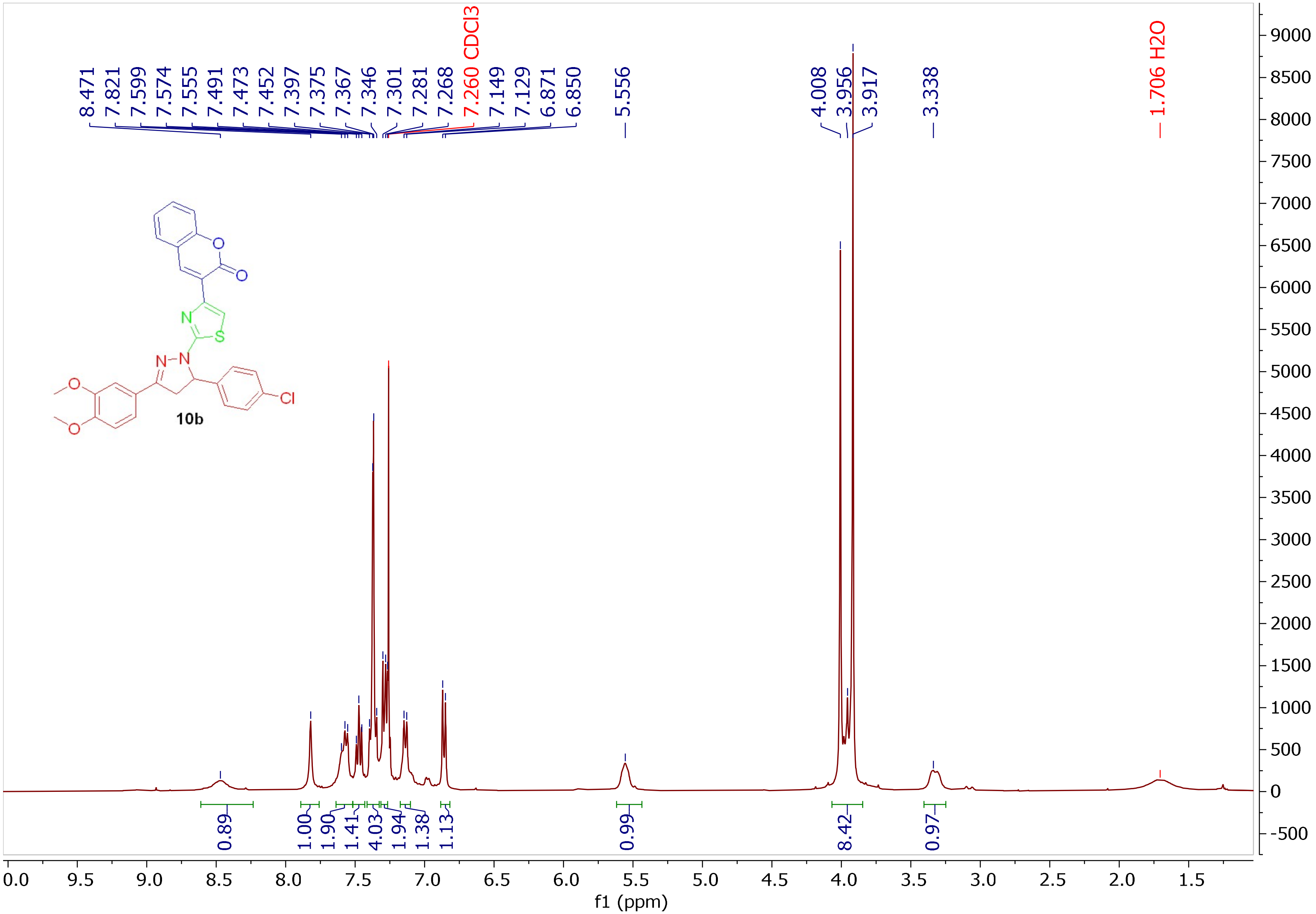

Figure S37: ^{1}H NMR of compound **10b**

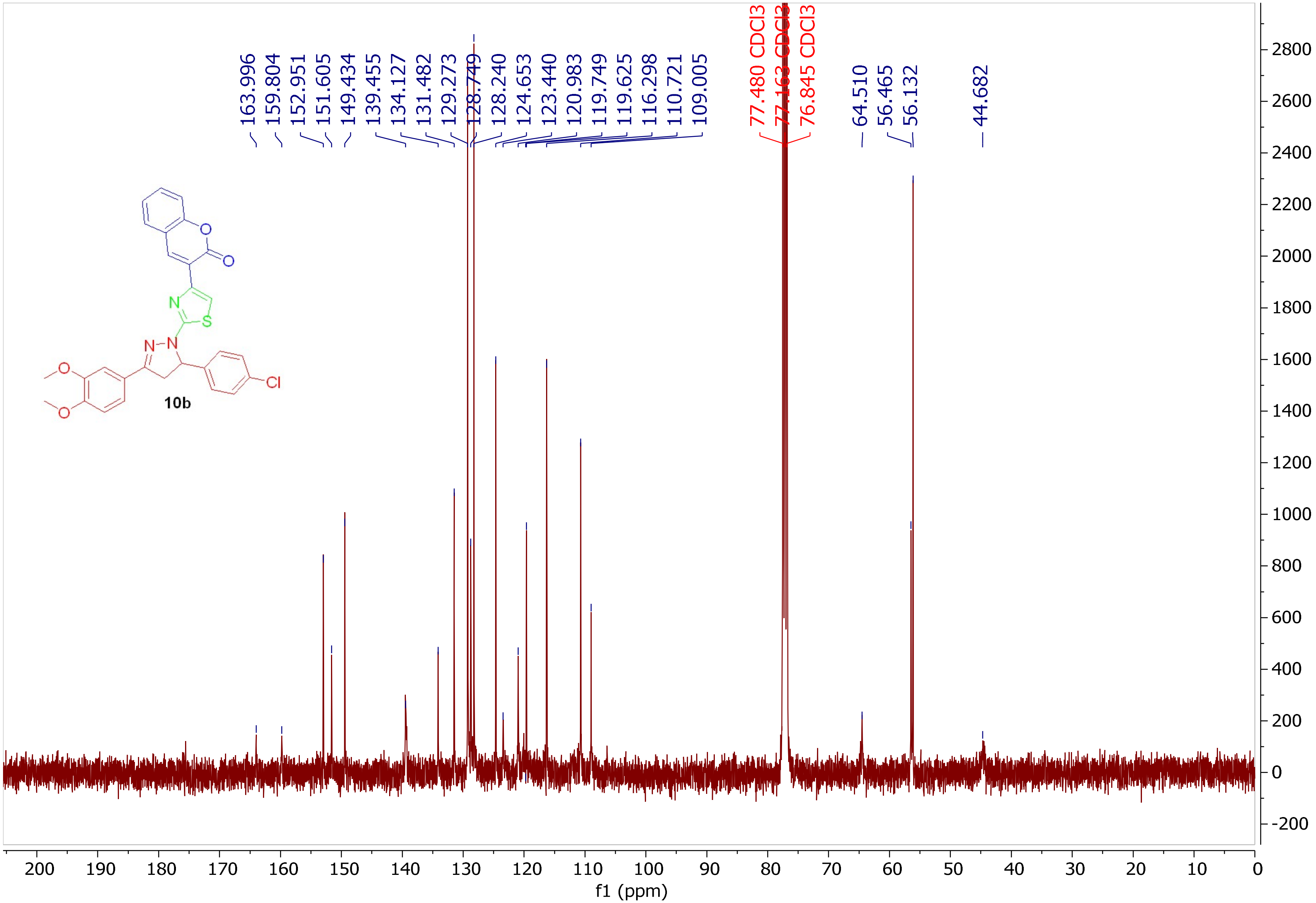

Figure S38: ^{13}C NMR of compound **10b**

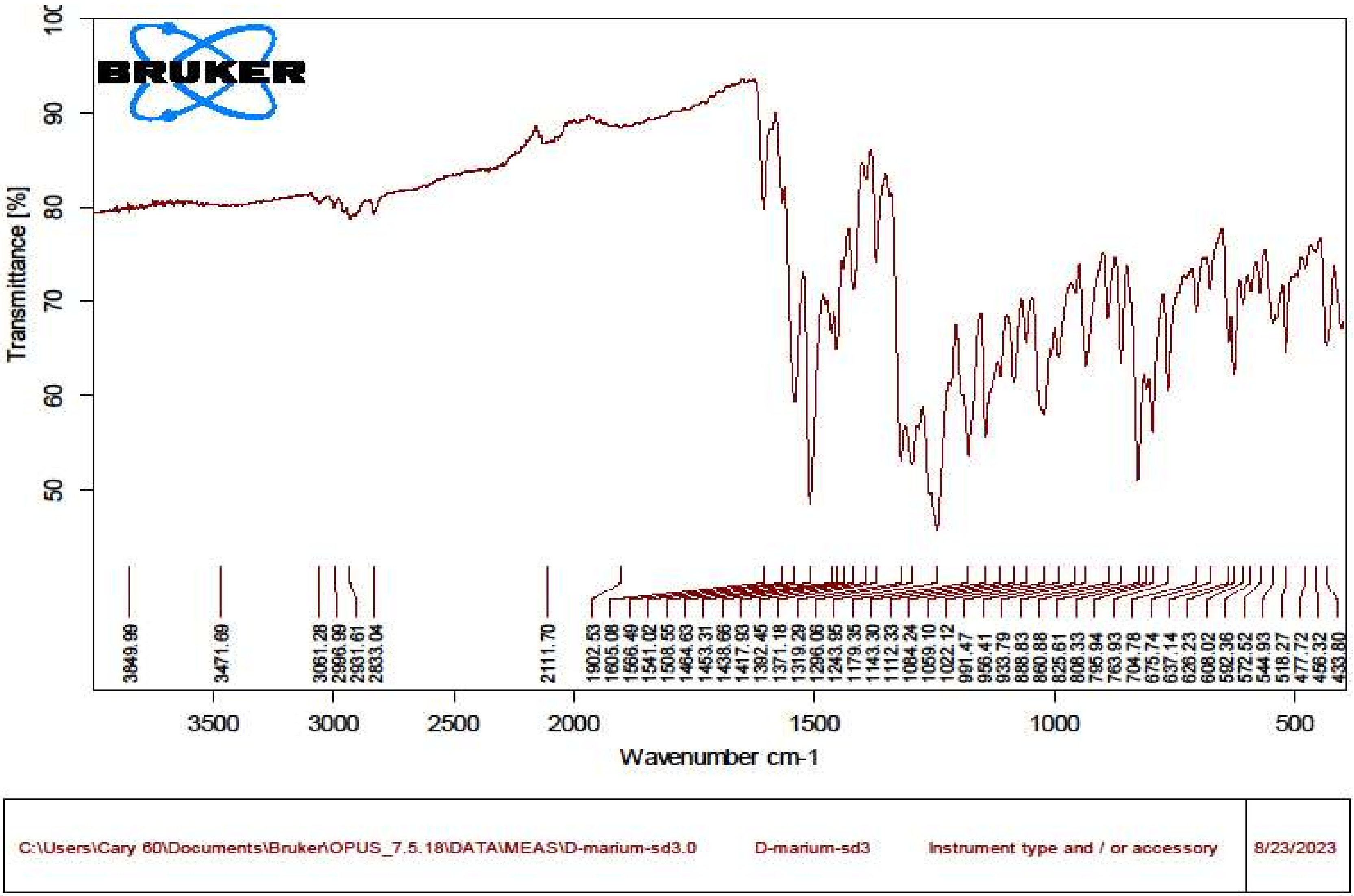

Figure S39: IR of compound **4a**

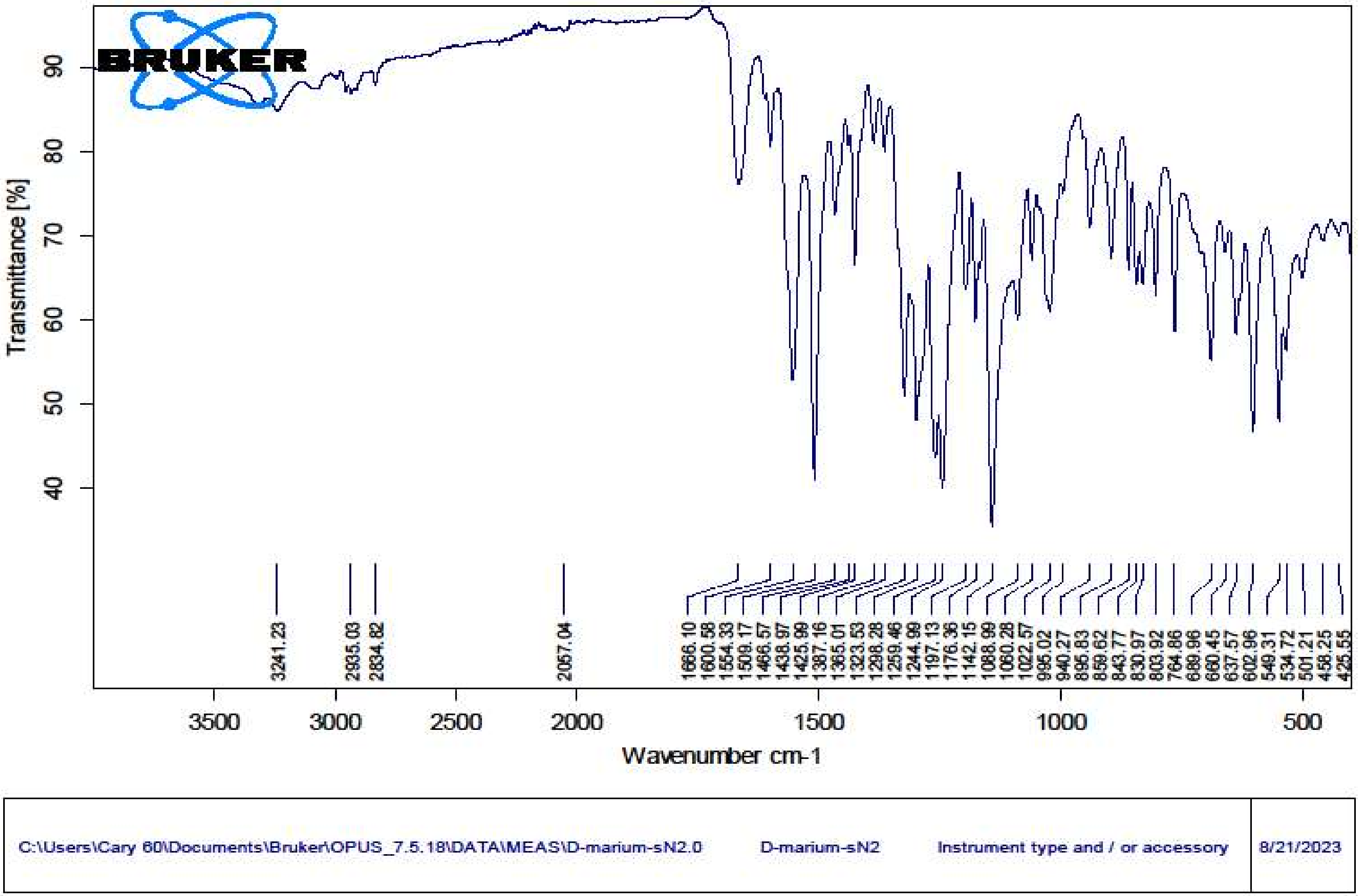

Figure S40: IR of compound **4b**

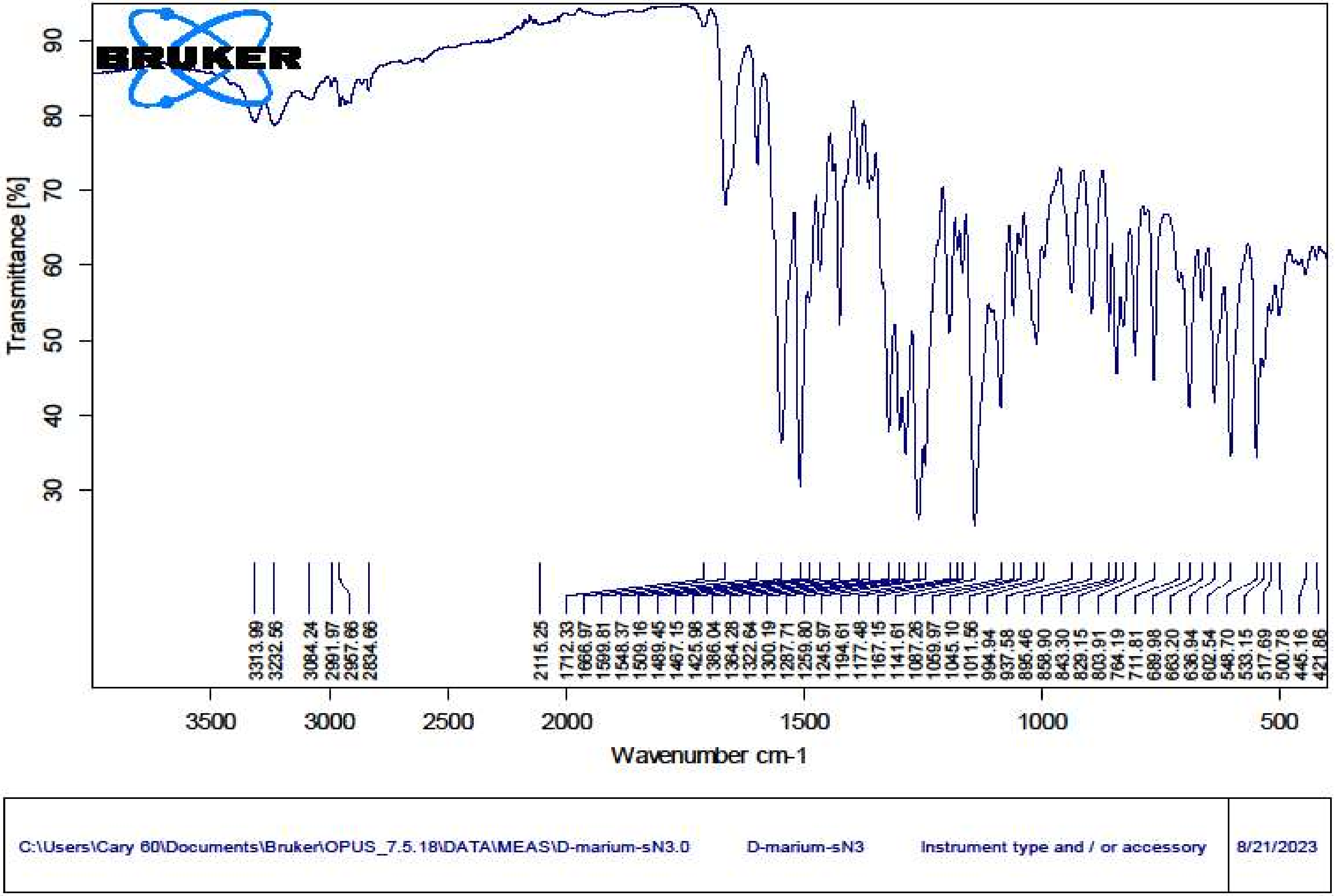

Figure S41: IR of compound **4d**

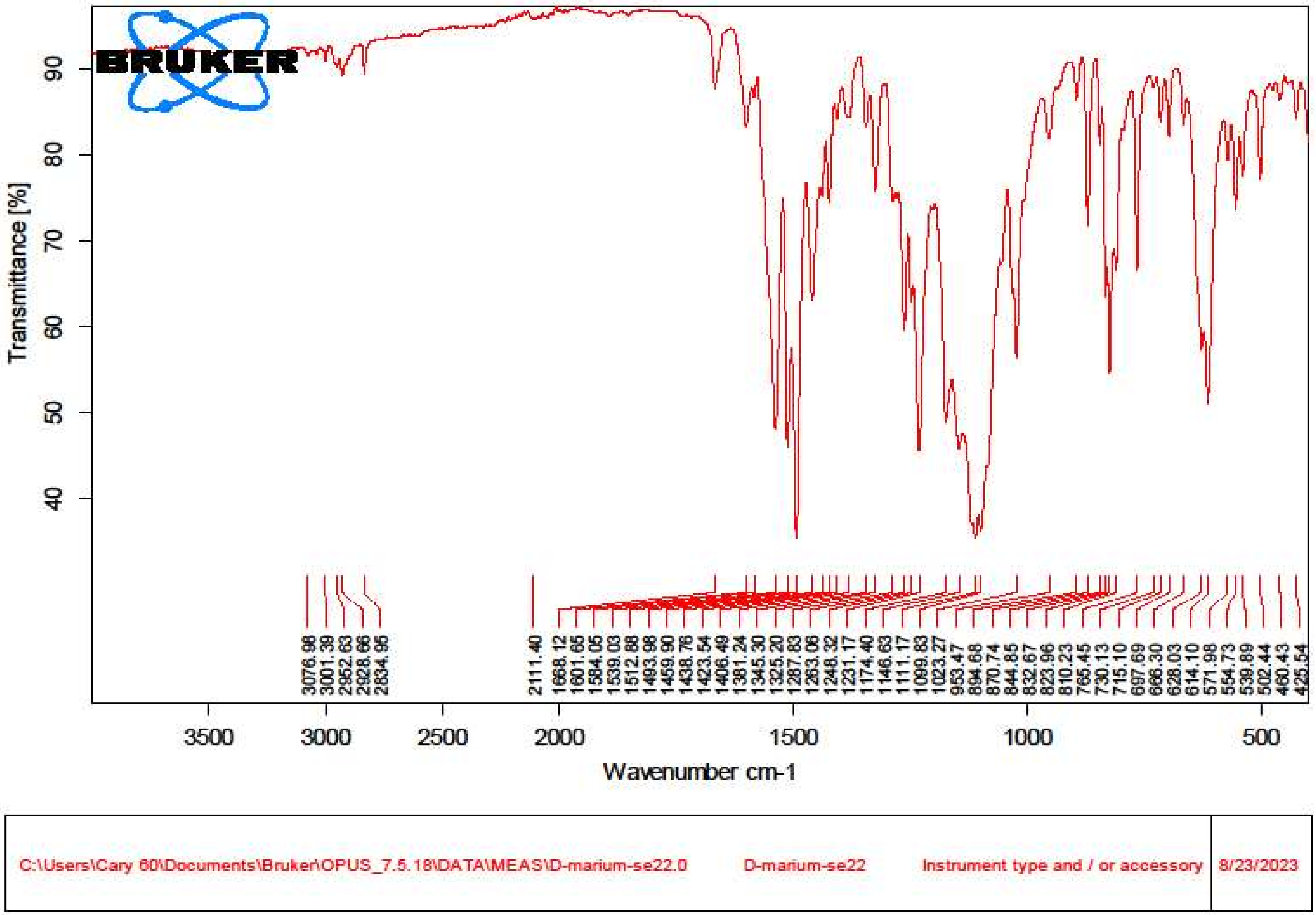

Figure S42: IR of compound **5a**

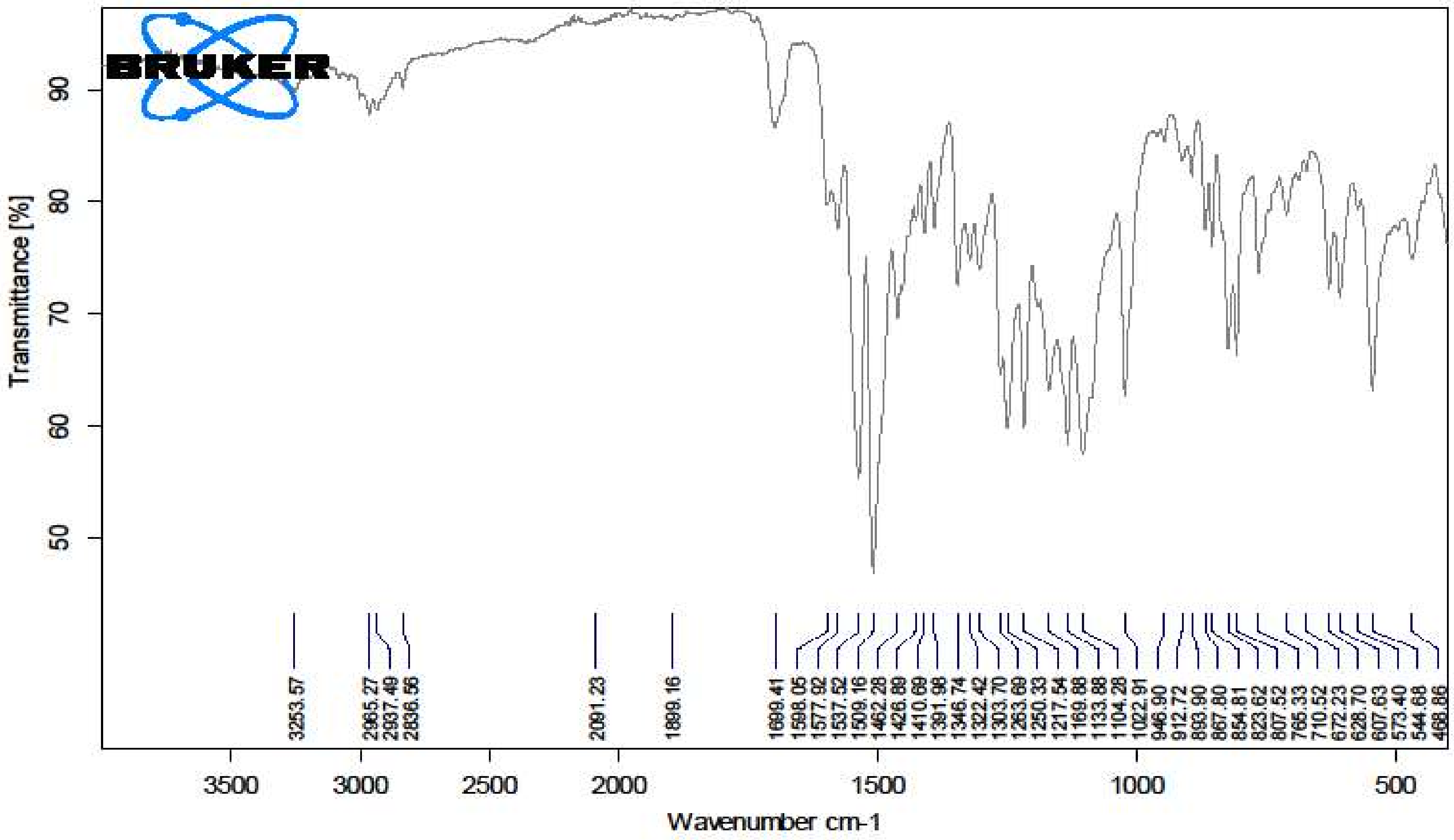

C:\Users\Cary 60\Documents\Bruker\OPUS_7.5.18\DATA\MEAS\D-marium-sc7.0 D-marium-sc7 Instrument type and / or accessory	8/17/2023

Figure S43: IR of compound **5b**

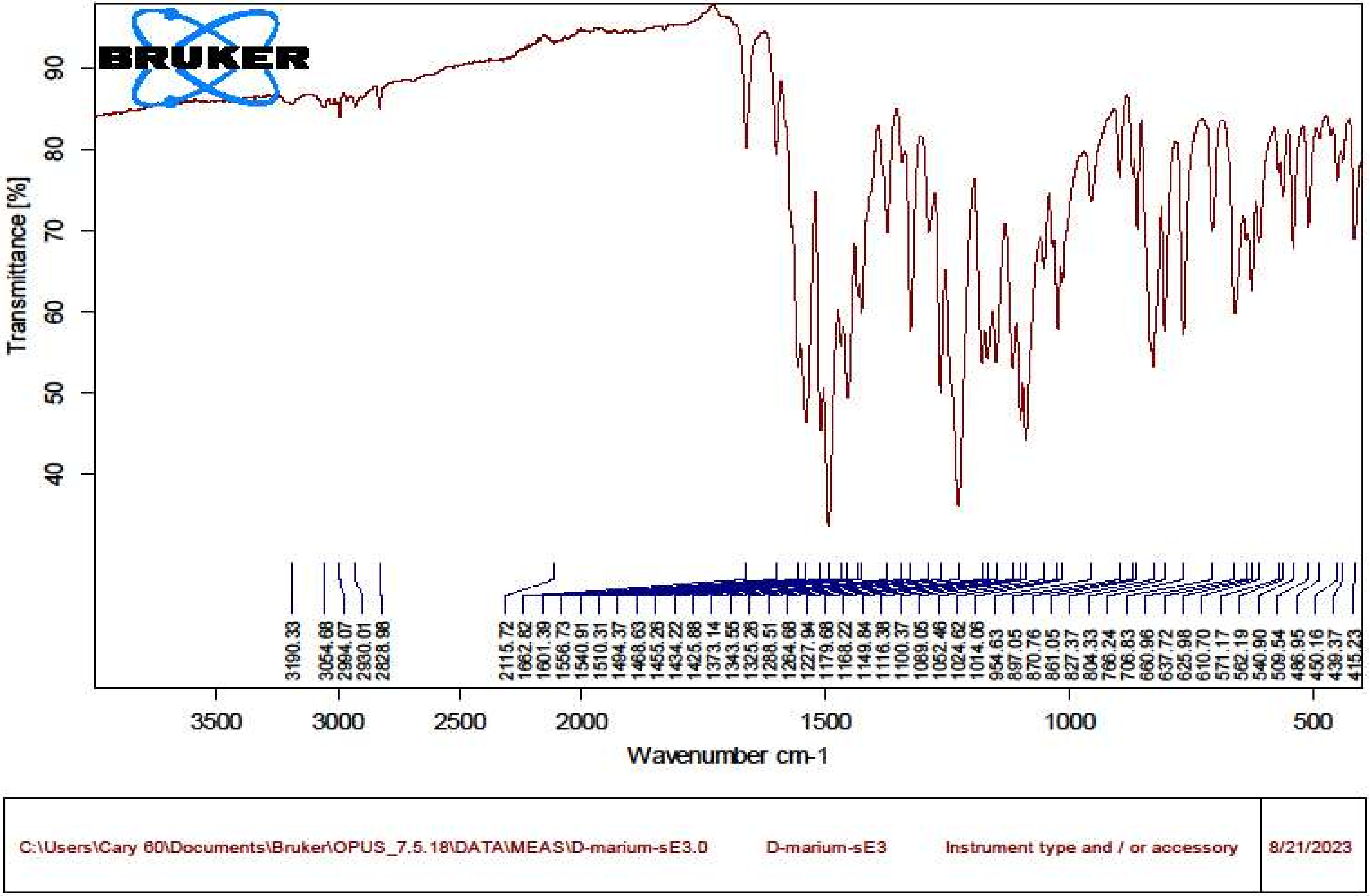

Figure S44: IR of compound **5c**

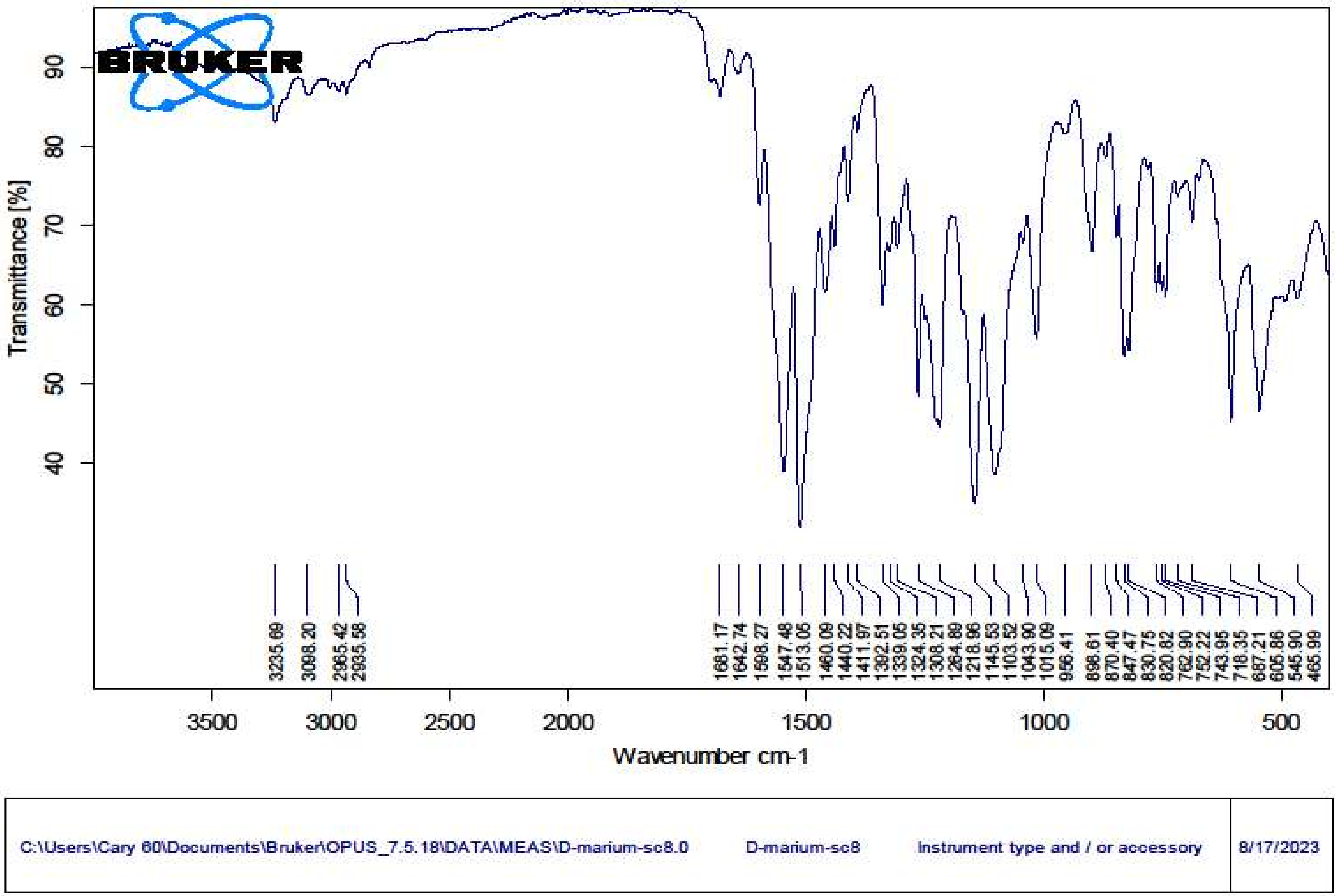

Figure S45: IR of compound **5d**

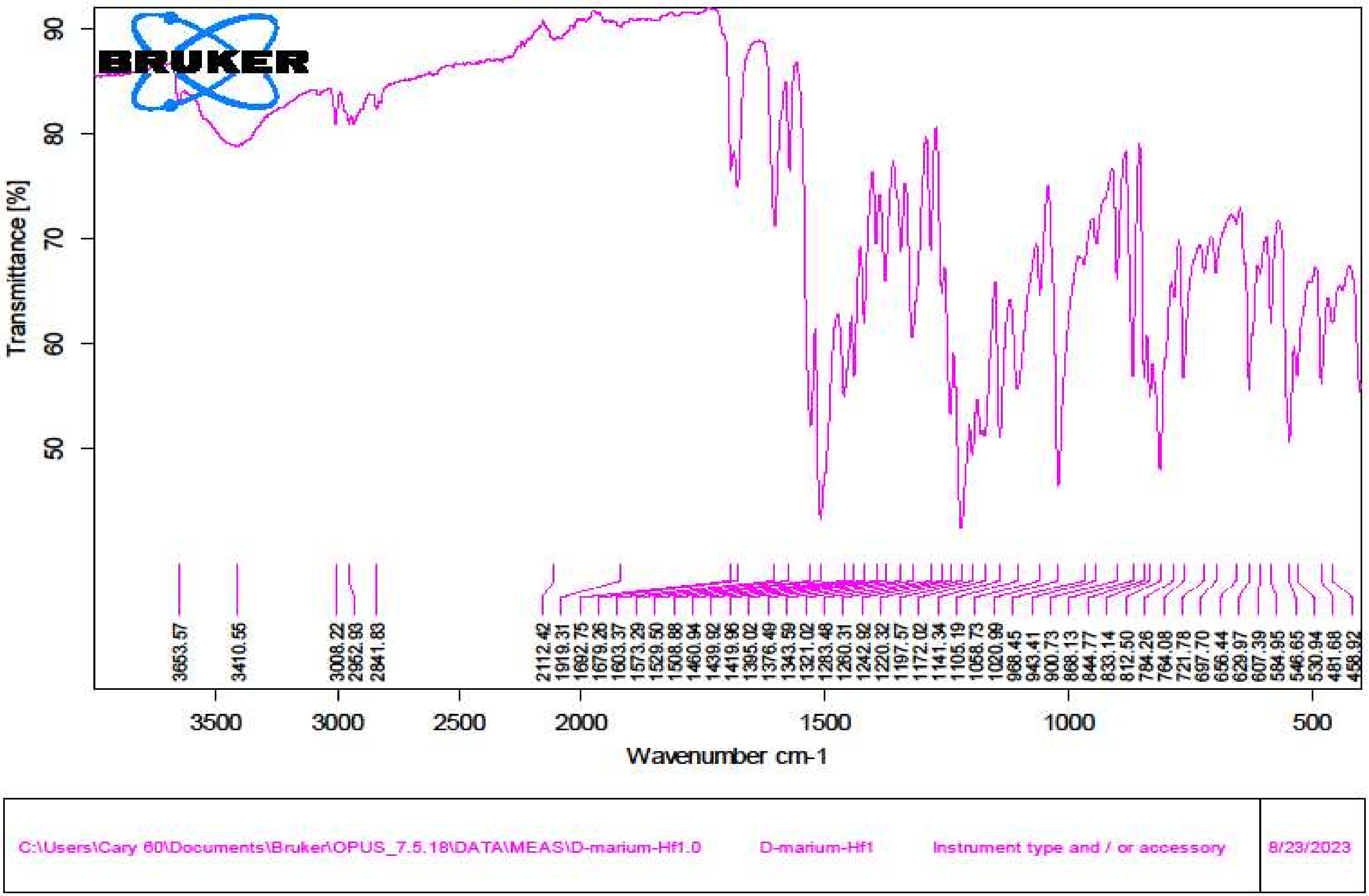

Figure S46: IR of compound **6a**

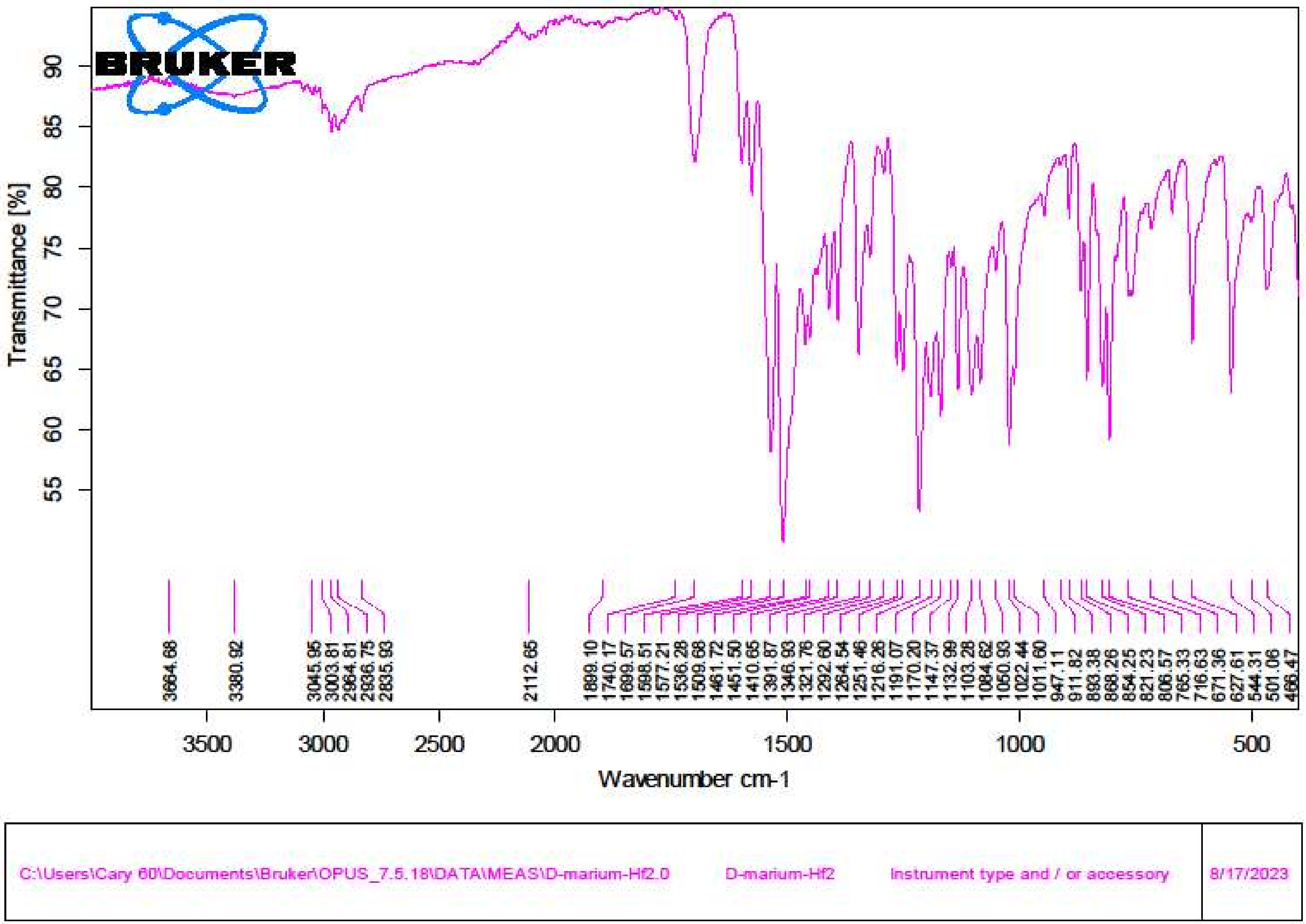

Figure S47: IR of compound **6b**

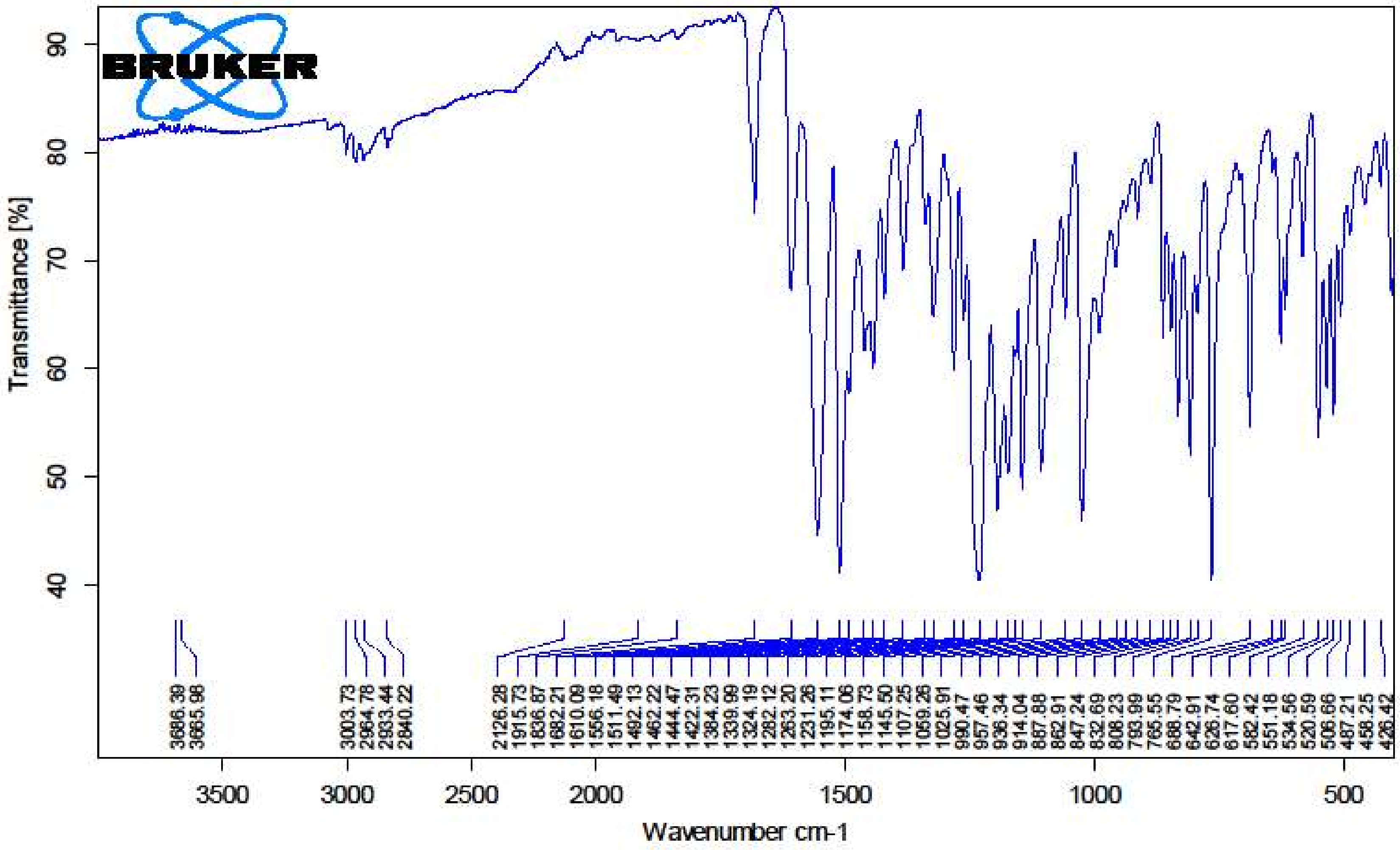

C:\Users\Cary 60\Documents\Bruker\OPUS_7.5.18\DATA\MEAS\D-marium-Nb12.0 D-marium-Nb12 Instrument type and / or accessory	8/17/2023

Figure S48: IR of compound **7a**

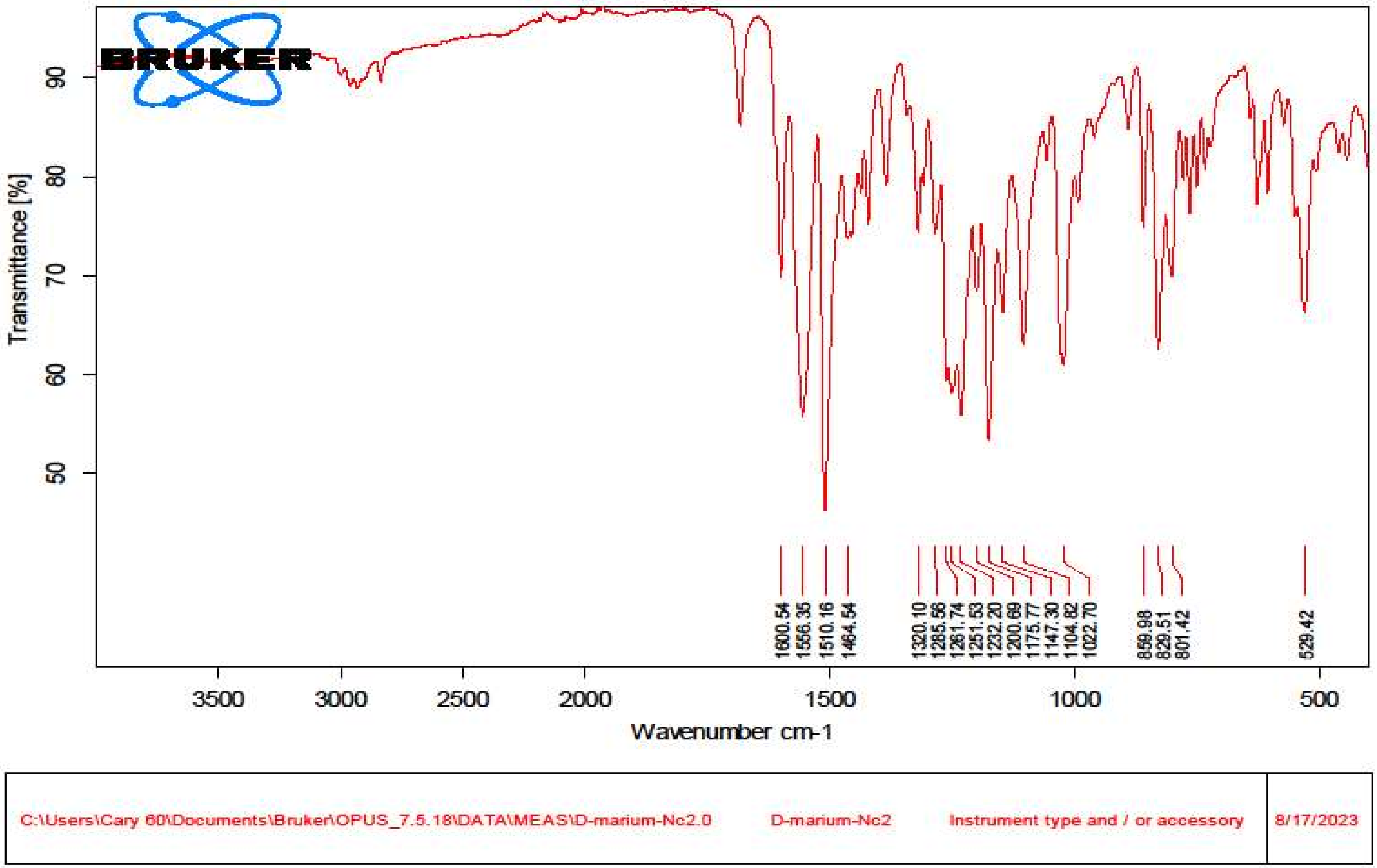

Figure S49: IR of compound **7b**

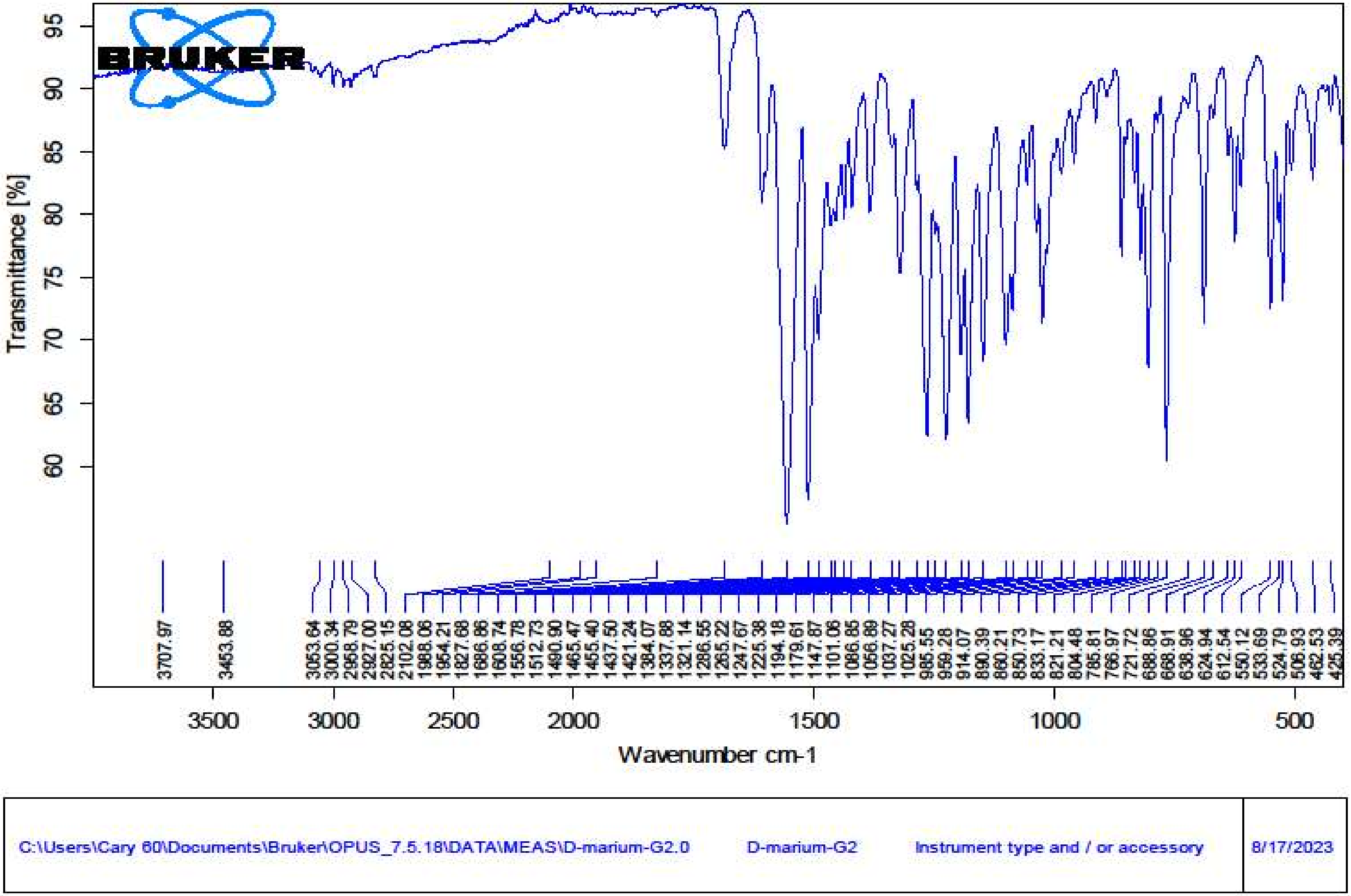

Figure S50: IR of compound **7c**

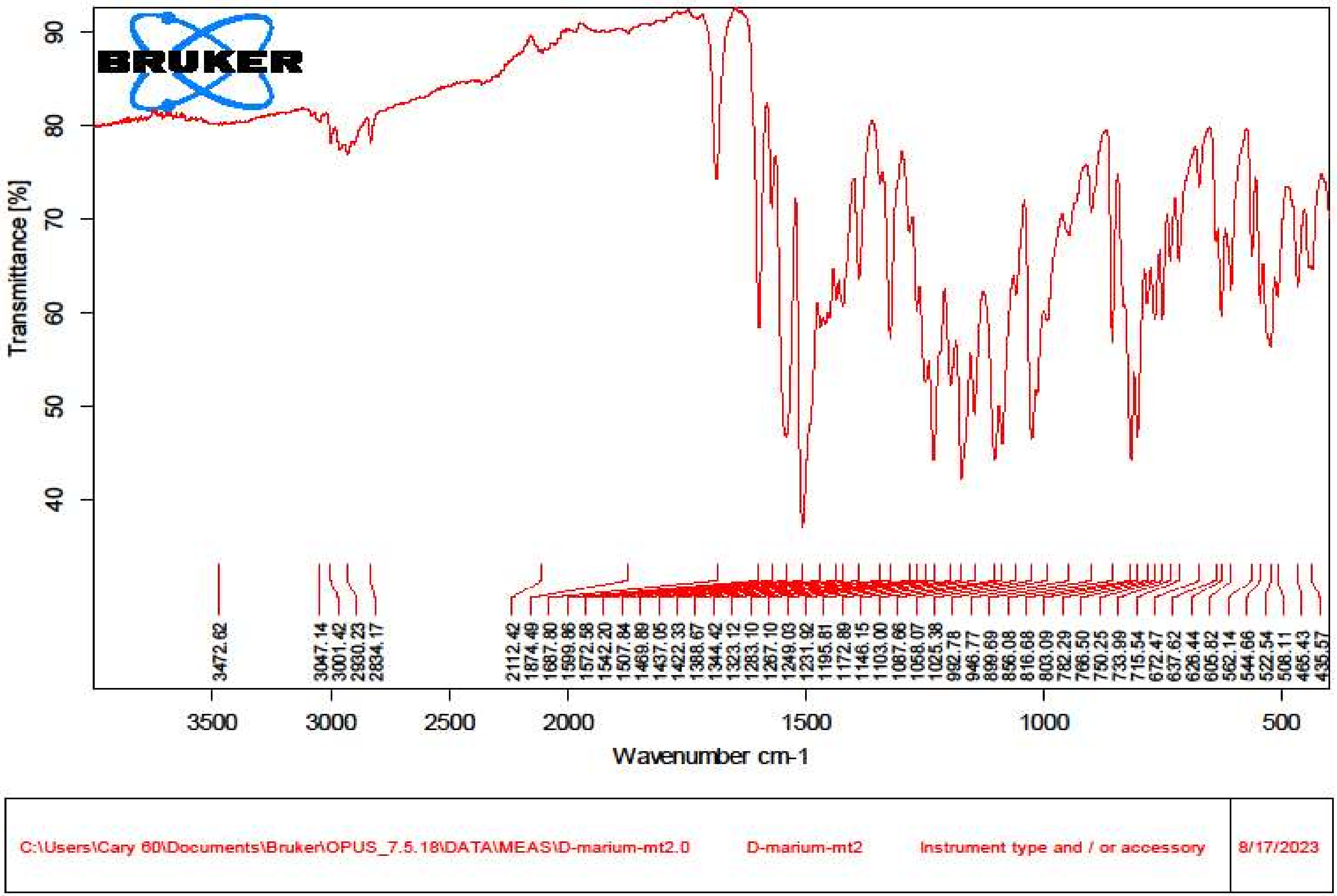

Figure S51: IR of compound **7d**

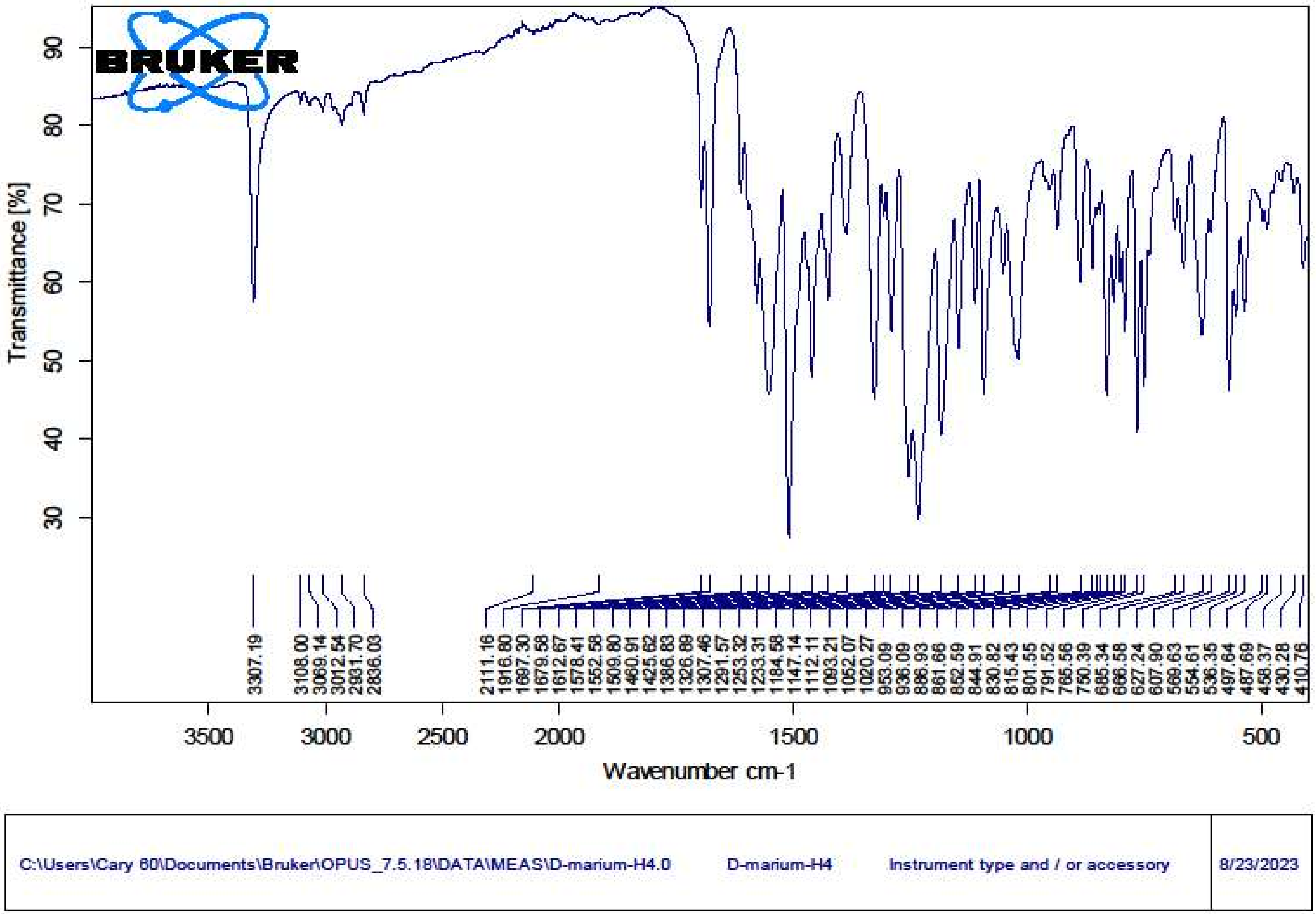

Figure S52: IR of compound **8a**

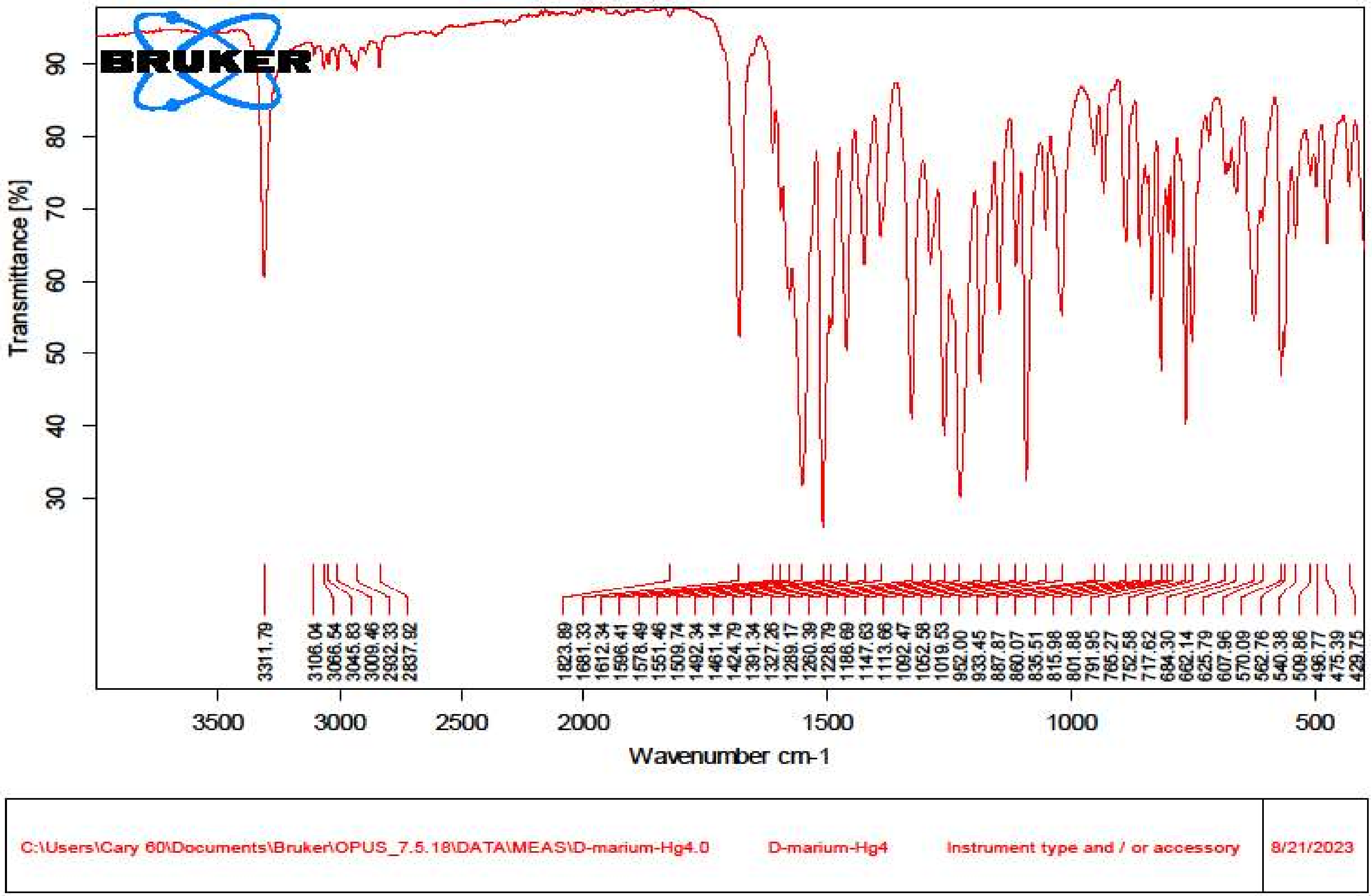

Figure S53: IR of compound **8b**

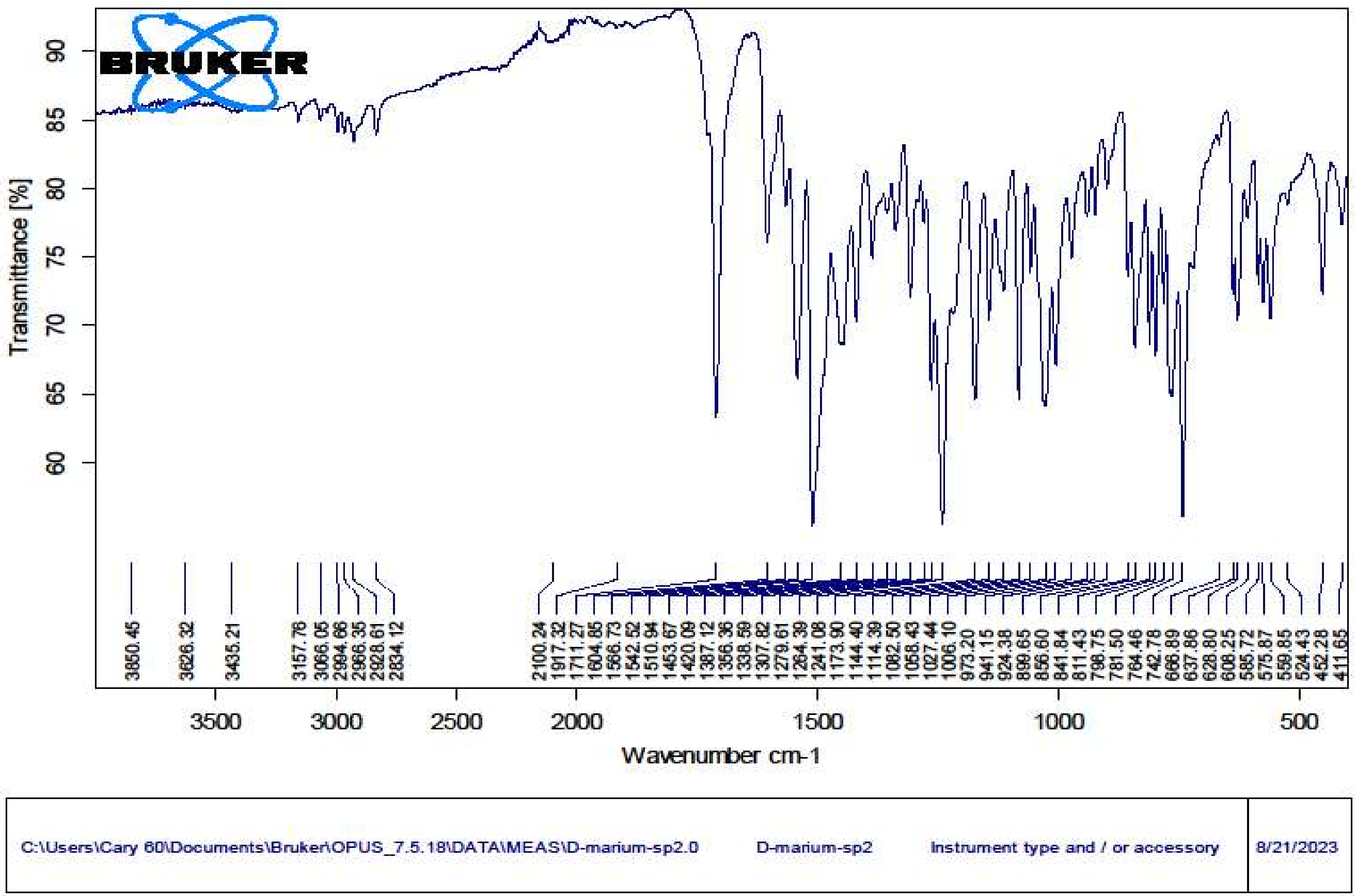

Figure S54: IR of compound **10a**

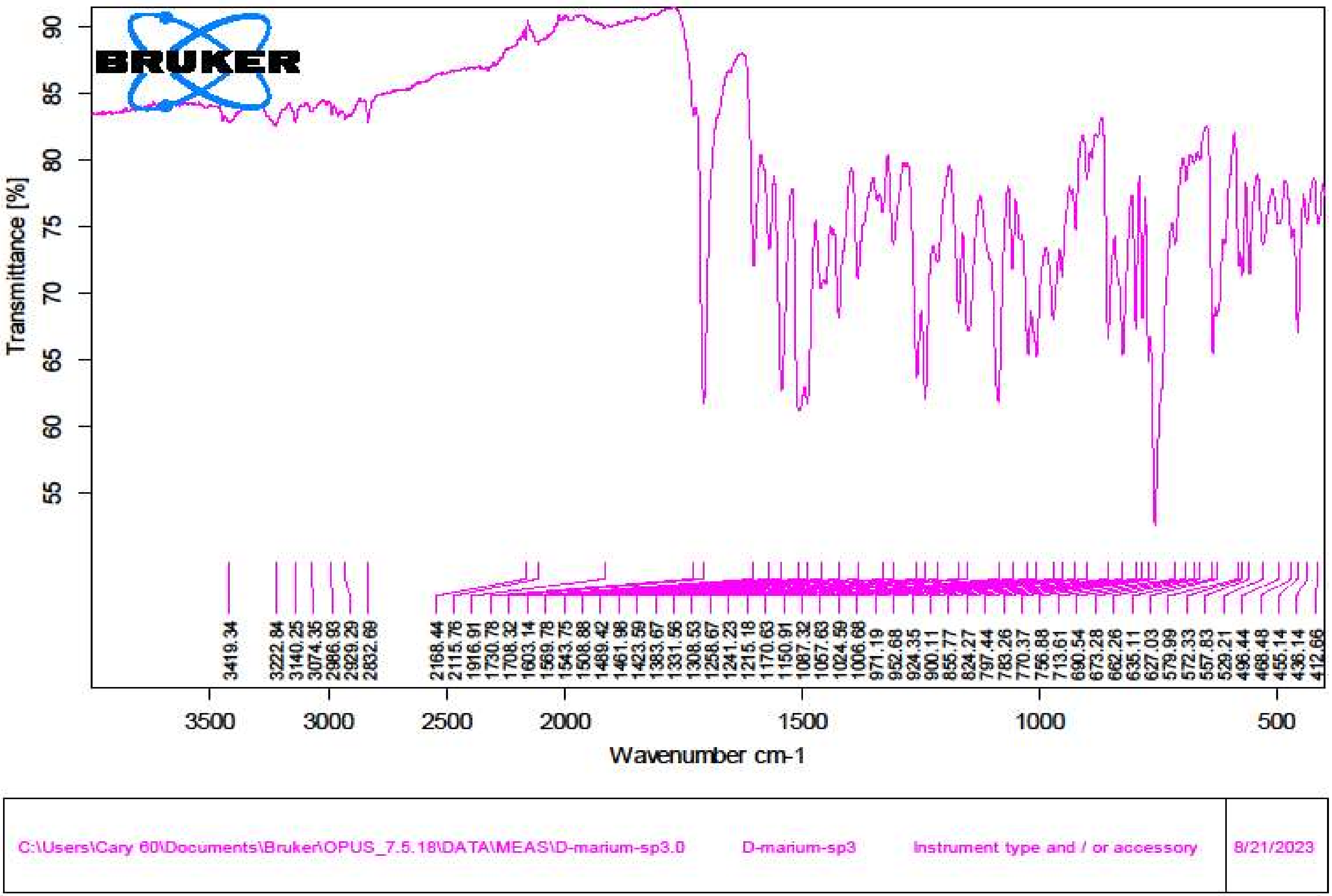

Figure S55: IR of compound **10b**

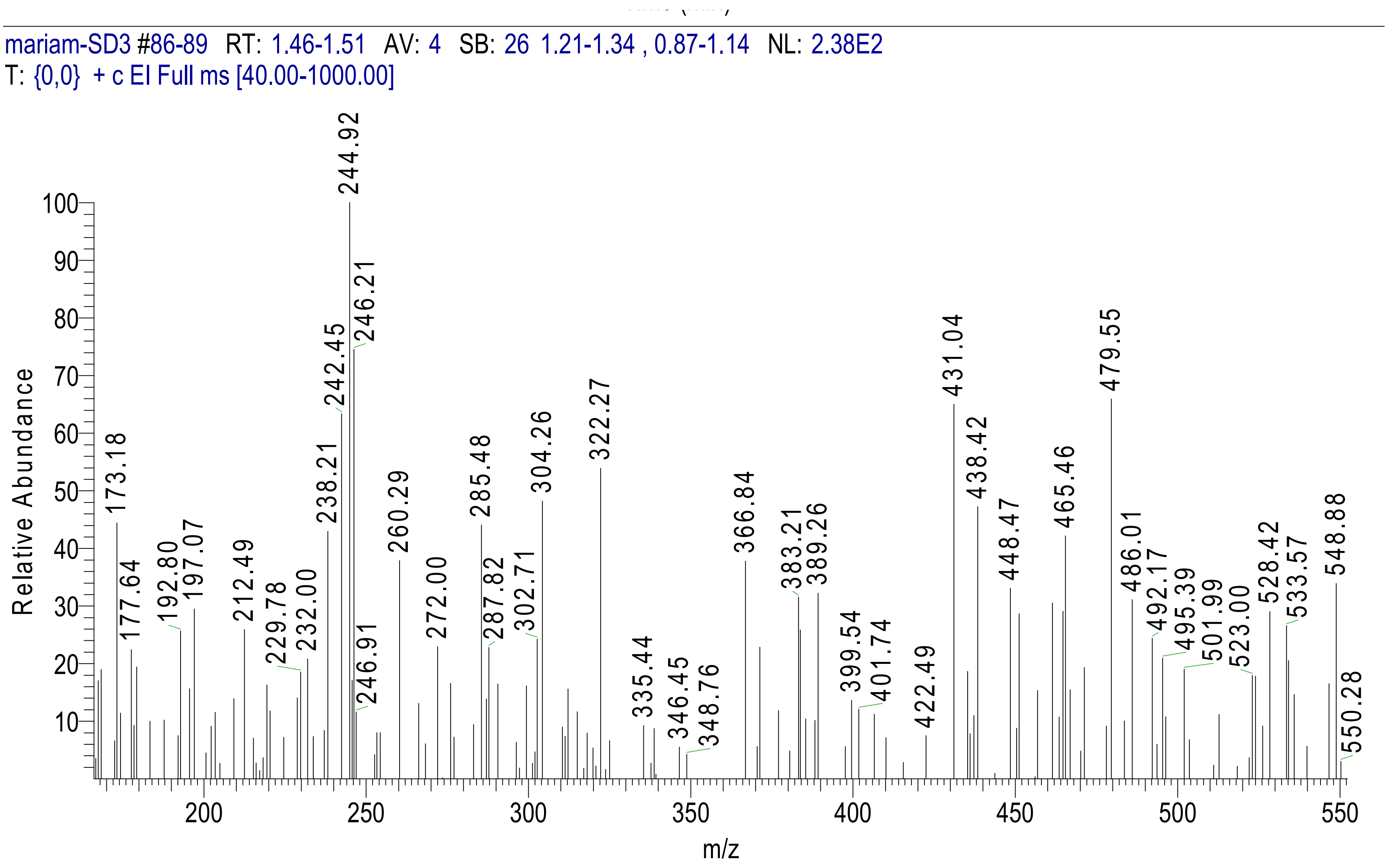

Figure S56: Mass of compound **4a**

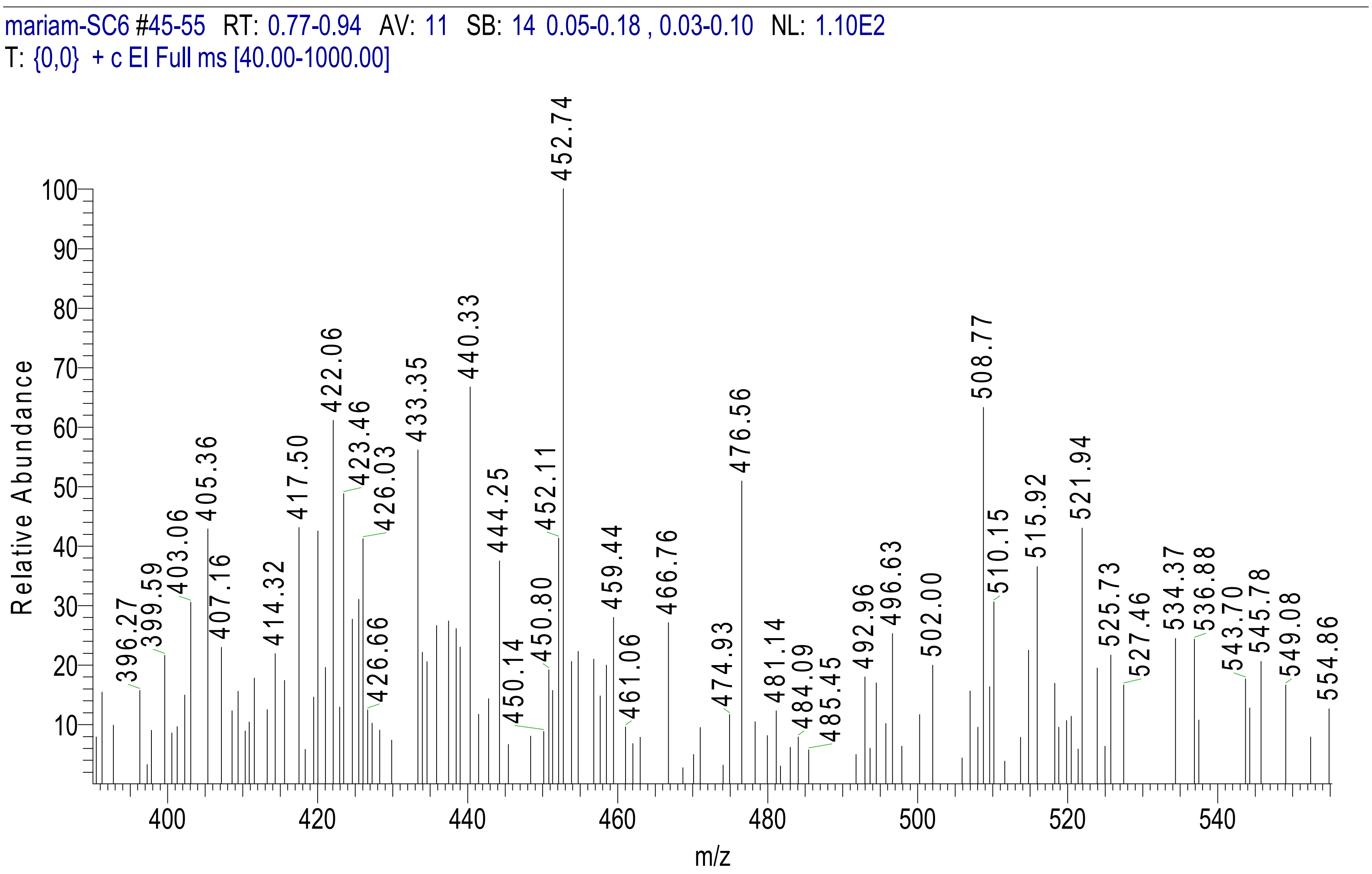

Figure S57: Mass of compound **4c**

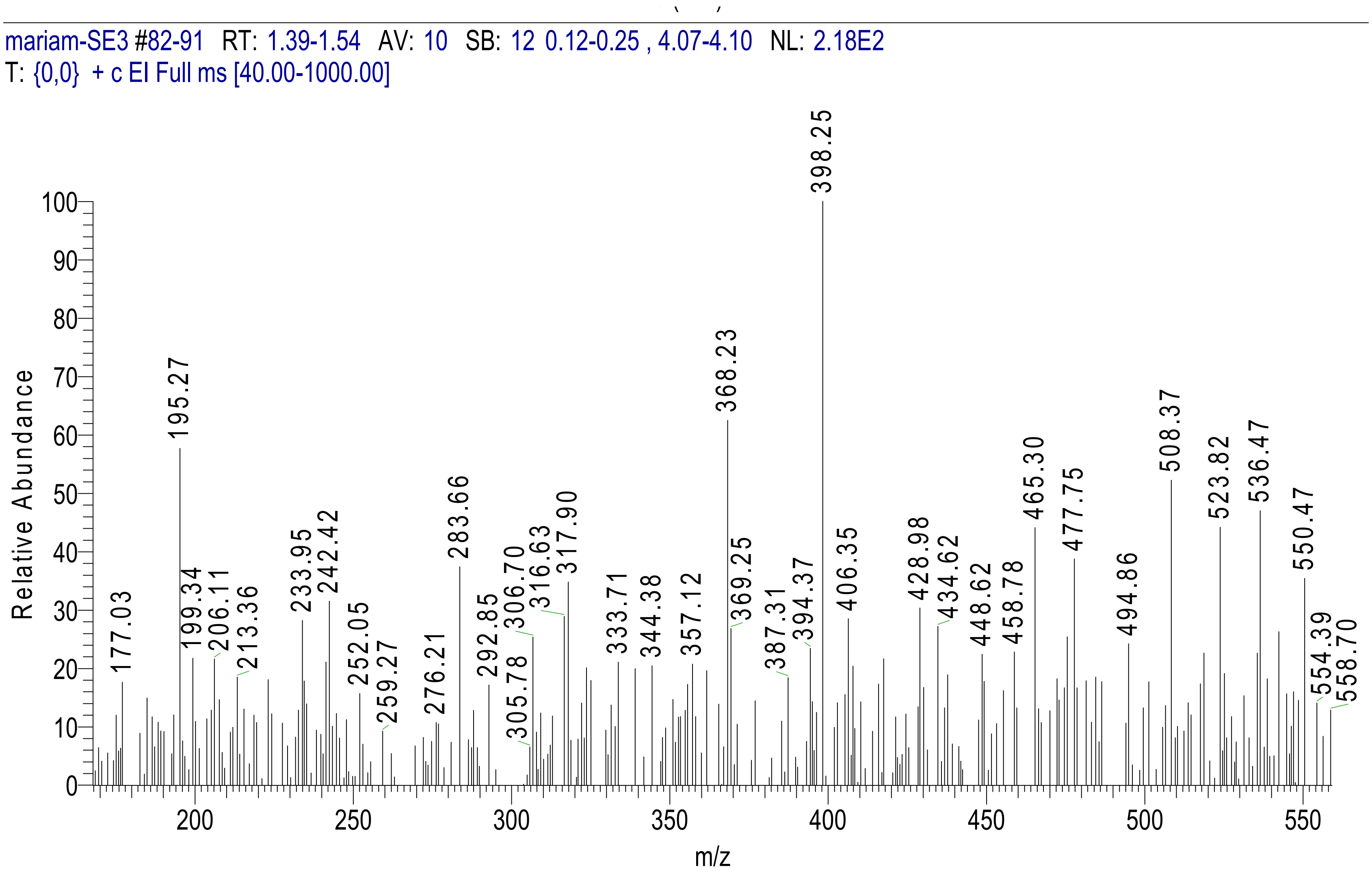

Figure S58: Mass of compound **5c**

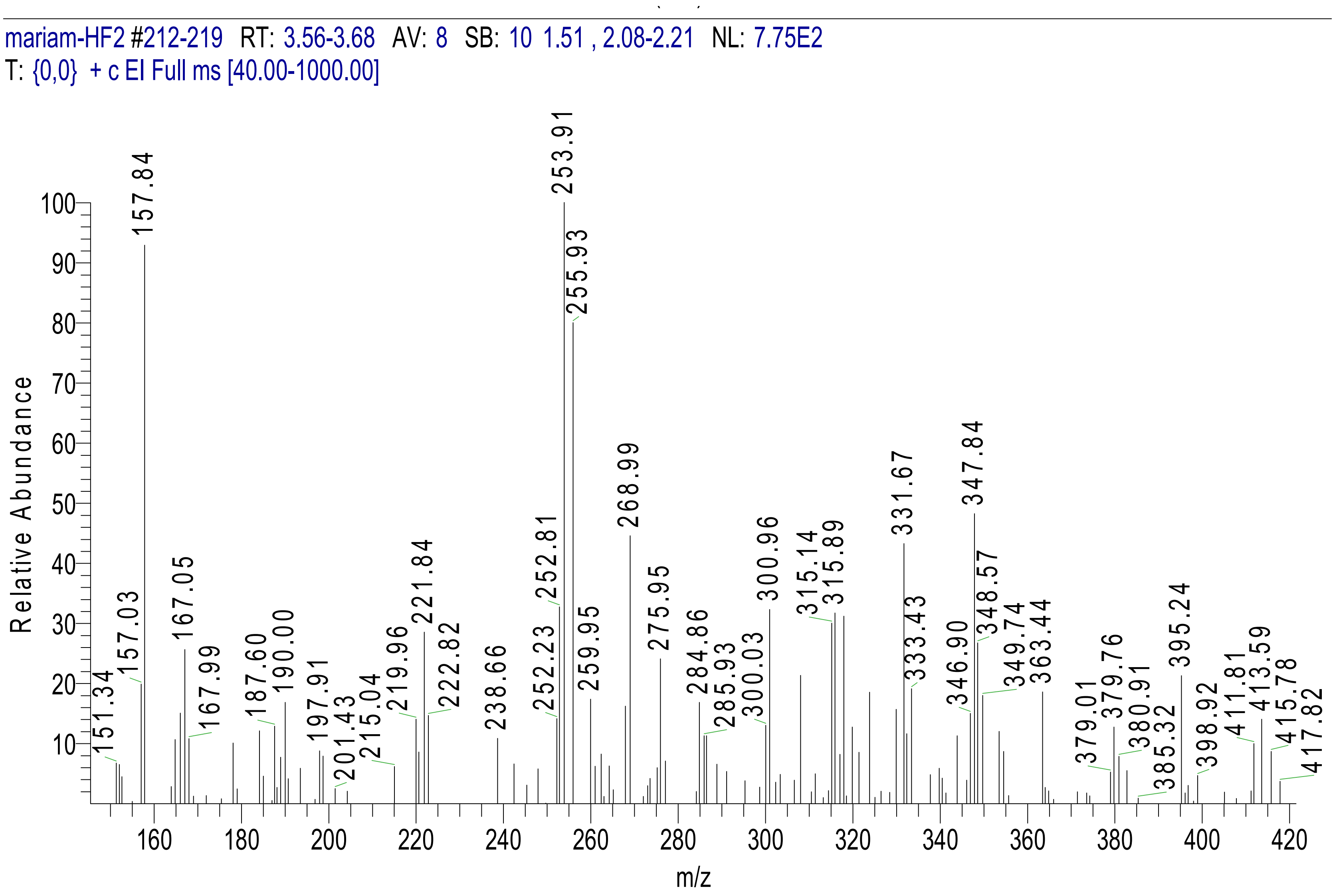

Figure S59: Mass of compound **6b**

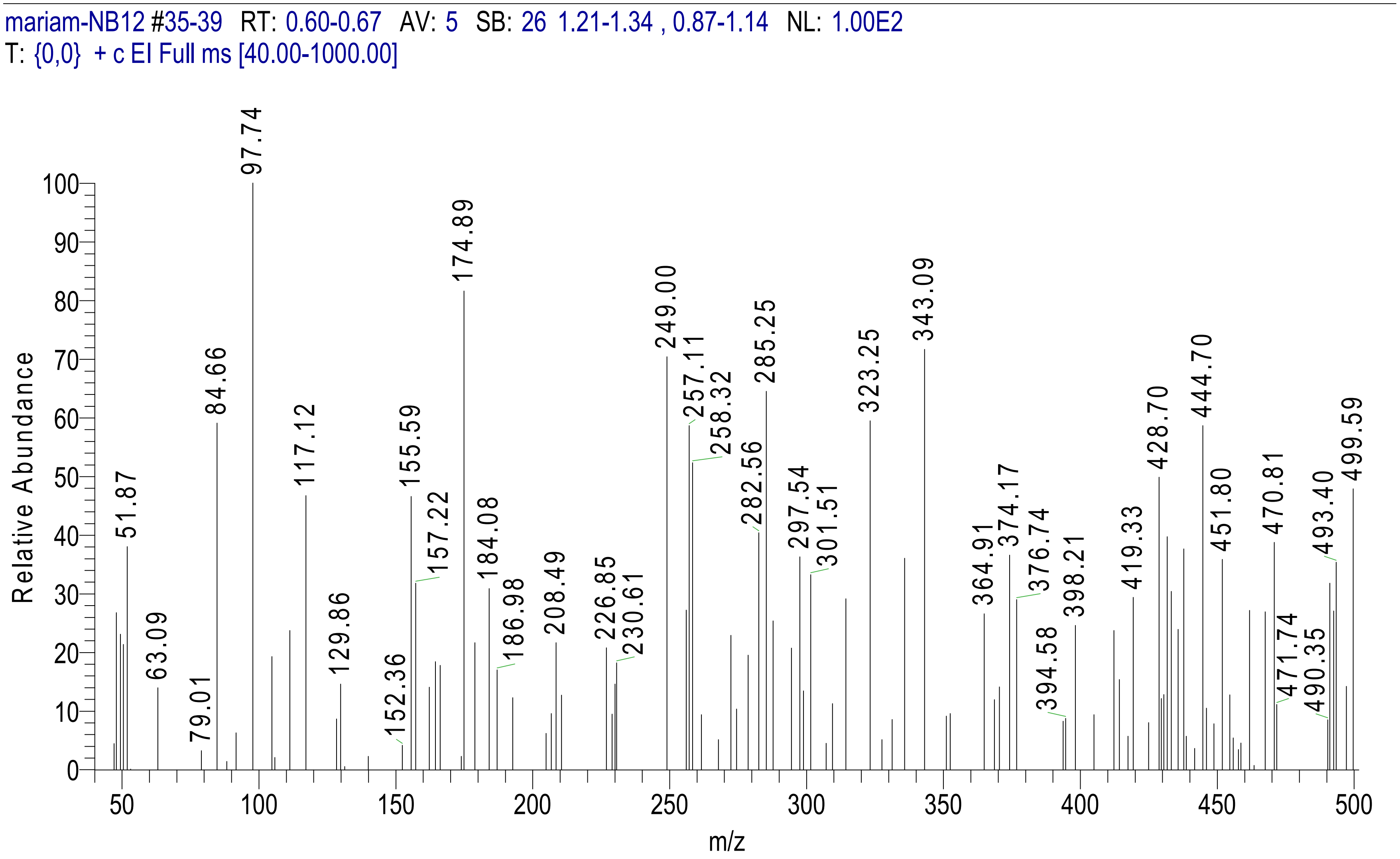

Figure S60: Mass of compound **7a**

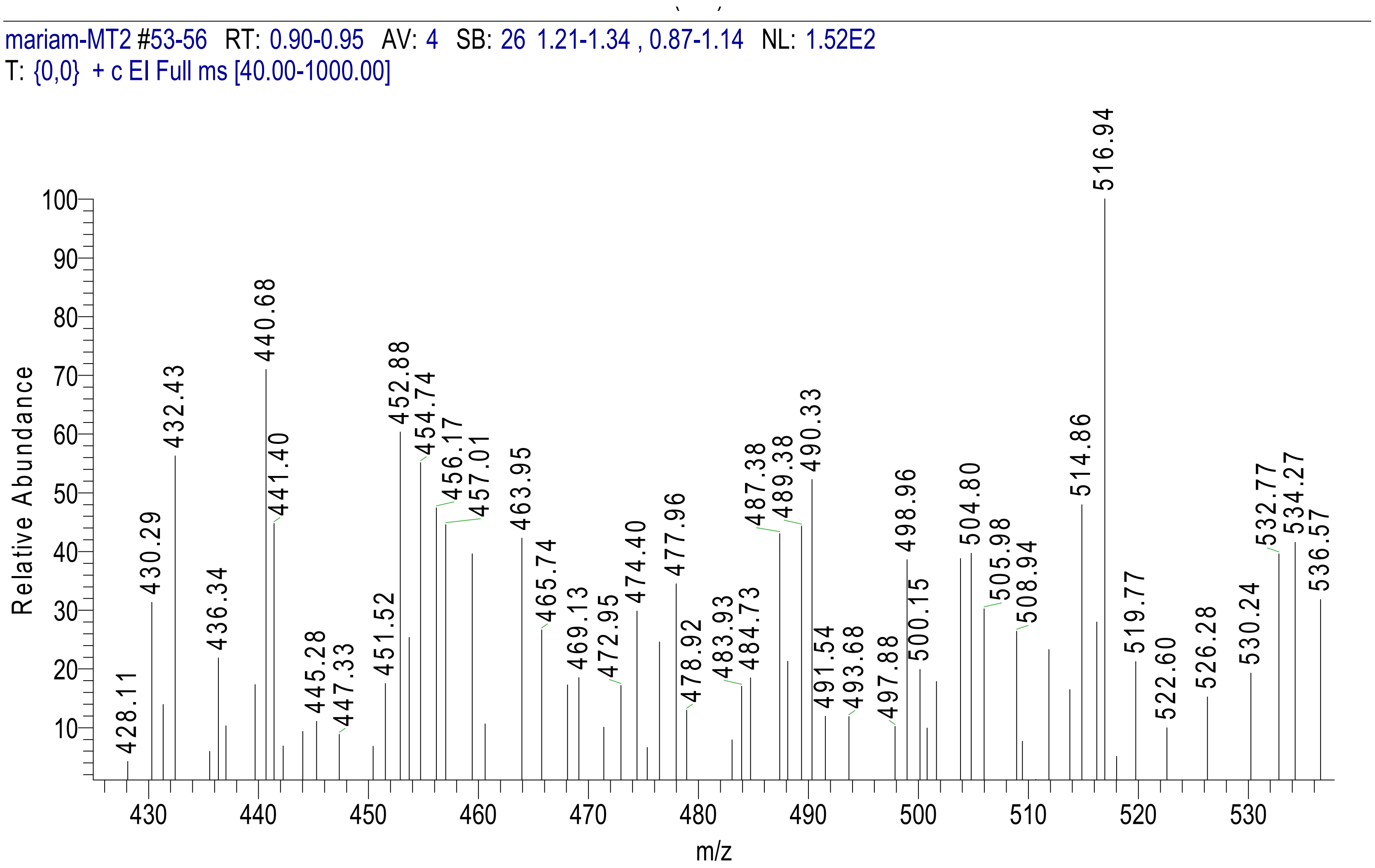

Figure S61: Mass of compound **7d**

Time (min)

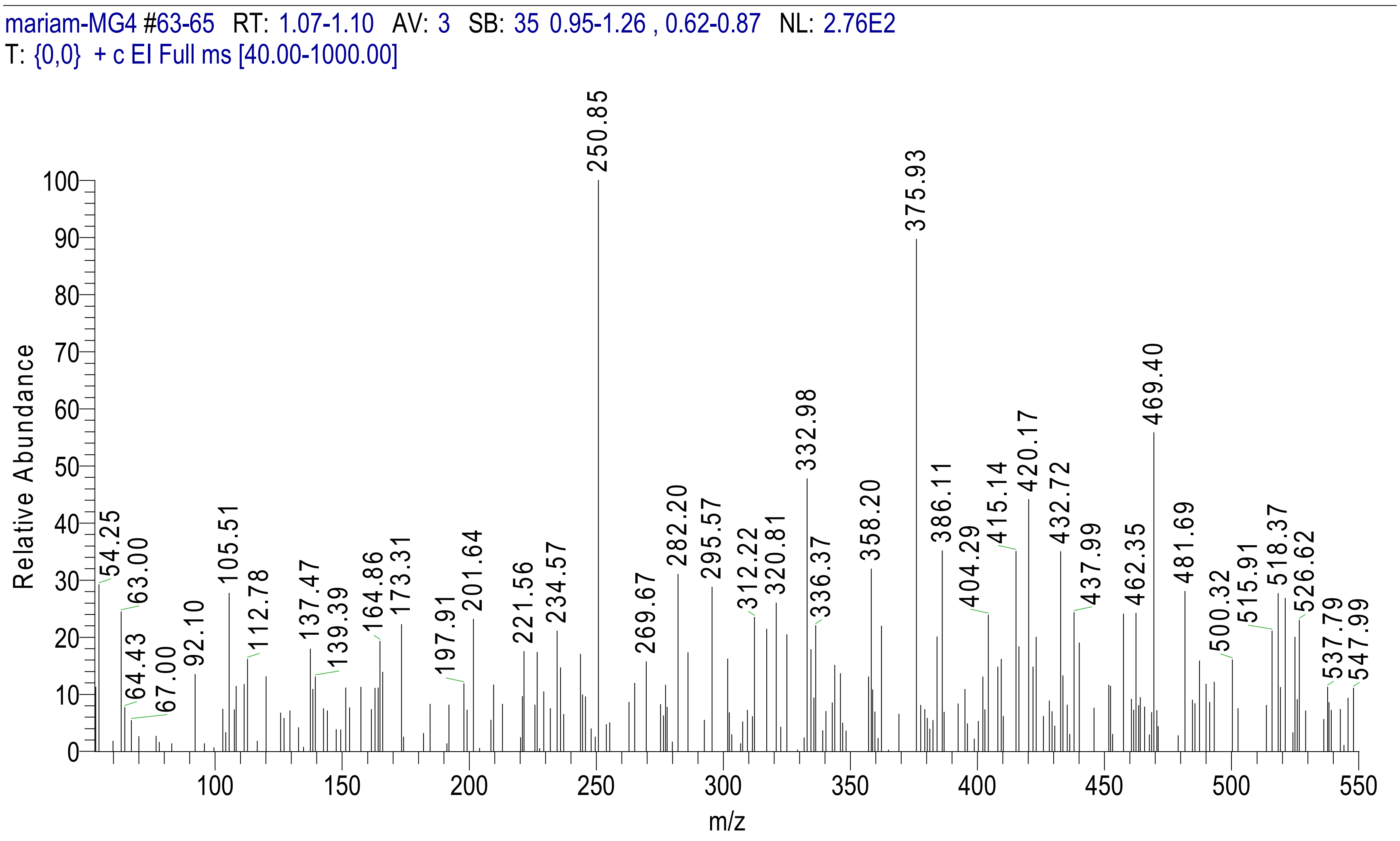

Figure S62: Mass of compound **8b**

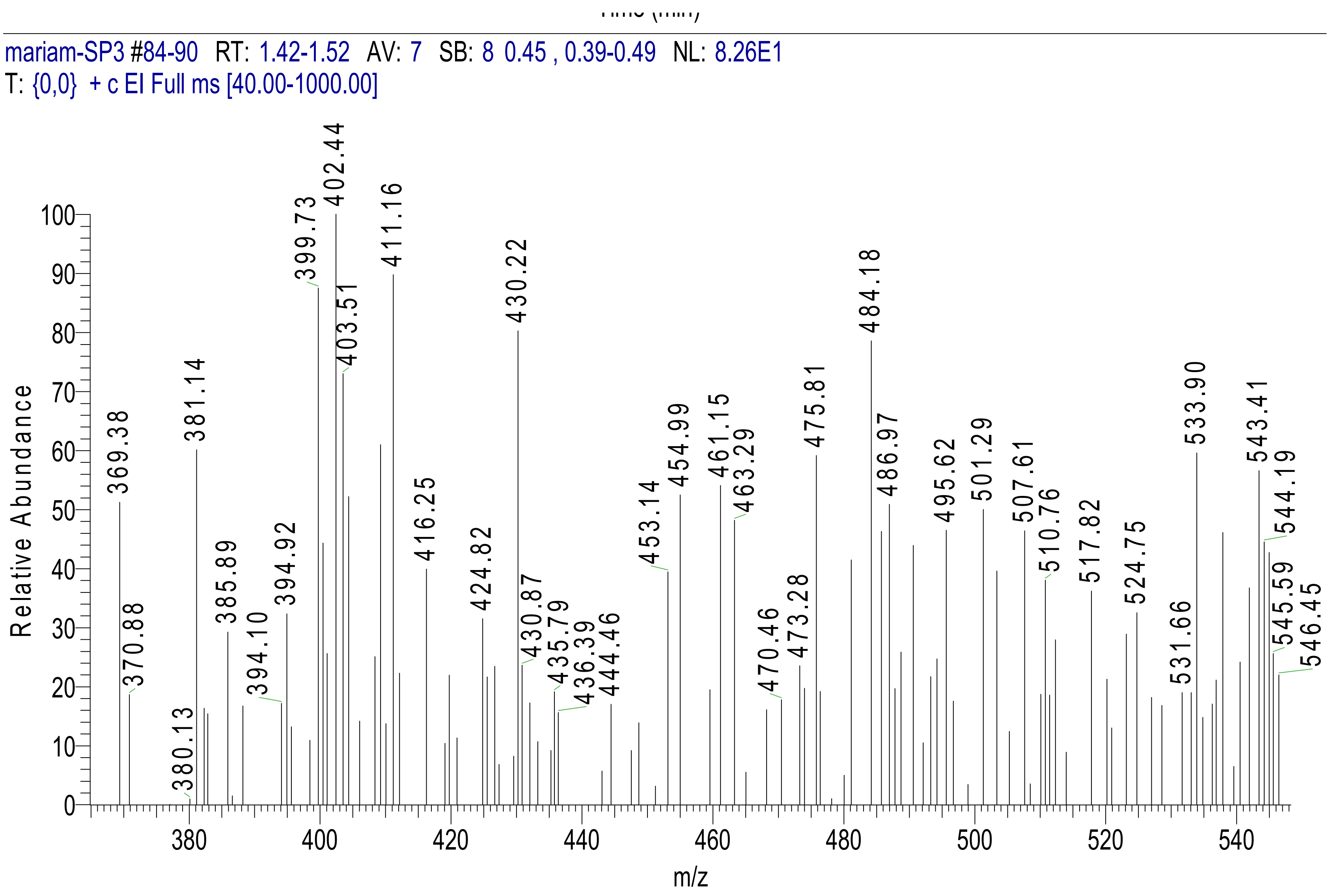

Figure S63: Mass of compound **10b**

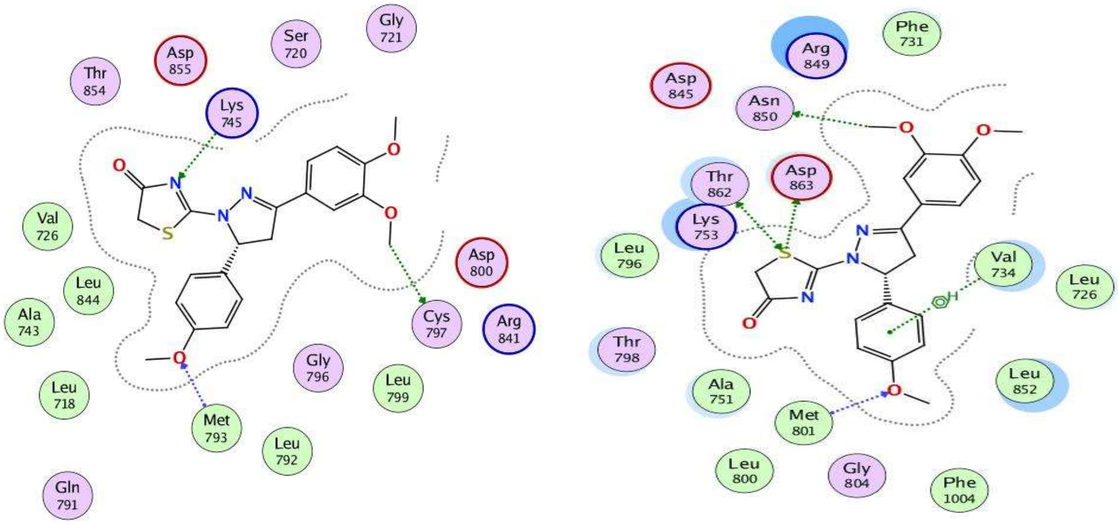

(A) 6a/EGFR **(B) 6a/HER2**

Figure S64: 2D binding interactions of **6a** in (A) EGFR and (B) HER2 binding pockets.

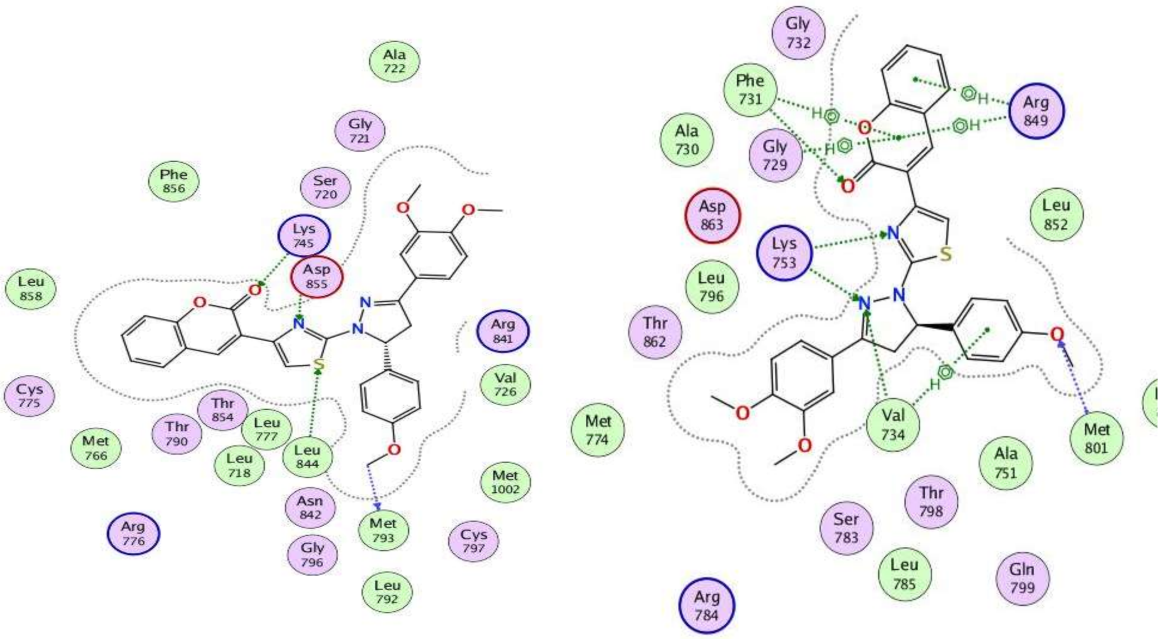

(A) 10a/EGFR **(B) 10a/HER2**

Figure S65: 2D binding interactions of **10a** in (A) EGFR and (B) HER2 binding pockets.

Table S1: SwissADME prediction of physicochemical properties and bioavailability of the target compounds.

Molecule	FCsp3	RB	HBA	HBD	MR	TPSA	CLog P	ESOL Class	GIA	BBB permeant	Pgp substrate	Lipinski violations	Veber violations	Bioavailability	PAINS #alerts	Brenk #alerts
**4a**	0.21	8	7	0	158	109.14	6.23	Poorly soluble	Low	No	Yes	1	0	0.55	1	1
**4b**	0.21	9	10	1	163.99	177.68	4.59	Poorly soluble	Low	No	No	2	1	0.17	1	1
**4c**	0.19	7	6	0	156.52	99.91	6.71	Poorly soluble	Low	No	Yes	2	0	0.17	1	1
**4d**	0.19	8	9	1	162.51	168.45	5.06	Poorly soluble	Low	No	No	1	1	0.55	1	1
**5a**	0.19	8	7	1	161.32	122.41	4.51	Poorly soluble	High	No	Yes	1	0	0.55	1	1
**5b**	0.19	9	10	2	167.31	190.95	2.92	Moderately soluble	Low	No	No	2	1	0.17	1	1
**5c**	0.15	7	6	1	159.84	113.18	5.09	Poorly soluble	High	No	No	1	0	0.55	1	1
**5d**	0.15	8	9	2	165.83	181.72	3.42	Poorly soluble	Low	No	No	2	1	0.17	1	1
**6a**	0.29	6	6	0	123.8	98.02	3.05	Moderately soluble	High	No	No	0	0	0.55	0	0
**6b**	0.25	5	5	0	122.32	88.79	3.55	Moderately soluble	High	No	No	0	0	0.55	0	0
**7a**	0.18	7	6	0	153.41	98.02	4.58	Poorly soluble	High	No	No	0	0	0.55	1	1
**7b**	0.21	8	7	0	159.91	107.25	4.54	Poorly soluble	High	No	Yes	1	0	0.55	1	1
**7c**	0.15	6	5	0	151.93	88.79	5.15	Poorly soluble	High	No	No	1	0	0.55	1	1
**7d**	0.18	7	6	0	158.42	98.02	5.16	Poorly soluble	High	No	No	1	0	0.55	1	1
**8a**	0.17	6	7	1	163.74	127.12	3.66	Moderately soluble	High	No	Yes	1	0	0.55	1	1
**8b**	0.14	5	6	1	162.26	117.89	4.27	Poorly soluble	High	No	Yes	1	0	0.55	1	1
**10a**	0.17	7	7	0	158.89	114.63	5.2	Poorly soluble	Low	No	No	1	0	0.55	0	1
**10b**	0.14	6	6	0	157.41	105.4	5.64	Poorly soluble	Low	No	No	1	0	0.55	0	1

Chemistry apparatus

1- Melting points are uncorrected and were determined using a Stuart melting point apparatus.

2- Infrared spectra (KBr) were recorded on Shimadzu FT-IR 8400S spectrophotometer.

3- ^{1}H NMR (400 MHz) and ^{13}C NMR (100 MHz) spectra were recorded by Bruker spectrometer using deuterated di-methyl sulfoxide (DMSO-d_6) or Chloroform ($CDCl_3$).

4- Mass spectroscopy analysis was recorded on GCMS-QP 1000EX Shimadzu Gas Chromatography MS Spectrometer

5- Elemental analyses were performed at the Regional Center for Microbiology and Biotechnology, Al-Azhar University.

6- The reaction progress was monitored using thin-layer chromatography (TLC)-precoated silica gel G plates, and visualized by irradiation with UV lamp (254 nm).

Experimental of biological evaluations

Cytotoxicity

Anti-proliferative activities were done using MTT colorimetric assay. MCF-7 and MCF-10A cells were maintained in RPMI-1640 medium L-Glutamine (Lonza Verviers SPRL, Belgium, cat#12-604F). Both cell lines were provided with 10% fetal bovine serum (FBS, Sigma-Aldrich, MO, USA) and 1% penicillin-streptomycin (Lonza, Belgium). Cells were incubated following standard tissue culture work. Cell viability was assessed after 48 h using MTT solution (Promega, USA). Absorbance was subsequently measured (at 570 nm) using ELISA microplate reader (BIO-RAD, model iMark, Japan). The viability was calculated relative to control.

EGFR and HER2 enzyme inhibition

Measurement of potential EGFR/ HER2 inhibitory activities was performed using EGFR Kinase Assay Kit (BPS Bioscience kit, Cat#40321) and HER2 Kinase Assay Kit (BPS Bioscience kit, Cat#40721), respectively. Four compounds **6a**, **6b**, **10a**, and **10b** with the highest cytotoxic activities were evaluated against the EGFR and HER2 using lapatinib as the reference drug. The following equation was used to determine the proportion of autophosphorylation inhibition by compounds: $100 - [\frac{A\ control}{A\ treated} - Control)]$ using the percentage inhibition curves of five concentrations for each compound.

Cell cycle analysis and apoptotic assay

MCF-7 cells were seeded into 6-well culture plates (3-5× 10^5 cells/well) and incubated overnight. Cells were then treated with compounds **6a** and **10a** at their respective IC_{50} values for 48 h. Subsequently, cells and medium supernatants were gathered and washed with ice-cold PBS. The cells were then suspended in 100 µL of the annexin binding buffer solution, which was described as "25 mM $CaCl_2$, 1.4 M NaCl, and 0.1 M Hepes/NaOH, pH 7.4". The cell incubation and addition of Annexin V-FITC solution, and propidium iodide (PI) were done according to the reported procedure. Next, the Cytoflex FACS equipment was used to obtain the stained cells. The program CytExpert was used to analyze the data.

Experimental of computational studies

Molecular modelling

The MOE (2019.0102) program was utilized for the molecular docking simulations. The two biological targets, crystal EGFR (PDB code: 1XKK) complexing with Lapatinib at 2.4 Å resolution and crystal HER2 (PDB code: 3RCD) complexing with TAK-285 at 3.21 Å resolution, were the sites where the active compounds 6a and 10a were docked. The above description covers the techniques for preparing proteins and molecules with the placement procedure.

In Silico SwissADME predictions

SwissADME is an online utility that is widely recognized for its reliability and availability for free. This tool is specifically designed to facilitate the calculation of physicochemical parameters of various compounds. The bioavailability and pharmacokinetic properties of the synthetic molecule can be established by uploading its structures to the website http://www.swissadme.ch/. The structures of the study compounds were imported into the SwissADME environment. Various physiological and pharmacokinetic parameters were obtained and exported in the form of a CSV file. Subsequently, the data was extracted and subjected to analysis.
